# Acylhydrobenzoquinones influence the susceptibility of *Staphylococcus aureus* towards abietanes and the speciation within *Plectranthus sensus lato*

**DOI:** 10.1007/s00044-026-03524-7

**Published:** 2026-01-13

**Authors:** Gabin Thierry M. Bitchagno, Sohini S. Bhatia, Scott Bintrim, Paula Coates, Debborah Mulligan, Monique S. J. Simmonds

**Affiliations:** 1 Royal Botanic Gardens Kew, Richmond, London, UK; 2 The Procter & Gamble Company, Mason Business Center, Mason, OH USA; 3 The Procter & Gamble Company, Reading, UK

**Keywords:** Metabolomic, Acylhydrobenzoquinones, *Plectranthus*, *Coleus*, Coleons, SAR

## Abstract

The genus *Plectranthus sensu lato* is known to produce abietanes, which exhibit significant potency against Gram-positive bacteria. It is hypothesized that abietanes exert their antibacterial effects by disrupting microbial cell walls. Recent studies have classified *Plectranthus s.l*. abietanes into six distinct groups. However, the impact of this chemical diversity on the response of abietanes against Gram-positive bacteria remains unexamined. Therefore, a structure-activity relationship study on abietanes from species of *Plectranthus sensu stricto* and *Coleus* was conducted to identify active fragments or pharmacophores in abietane-type diterpenoids that could be responsible for their activity against the Gram-positive bacteria *Staphylococcus aureus*. The study followed a bio-guided approach and included chemical profiling of the extracts, performed using NMR spectroscopy, and comparative analysis, using LC-MS in both positive and negative ionization modes. Results revealed all six classes of abietanes occurred as major constituents of the extracts, with distinctive chemical markers differentiating between *Plectranthus s.s*. and *Coleus* species. Oxidation of the B-ring in acylhydrobenzoquinones was identified as a key factor contributing to the strong antibacterial activity of *Plectranthus s.l*. derived compounds against *S. aureus*. Analysis of 28 species of *Plectranthus s.l*. revealed four main clusters based on the accumulation of various abietane classes. The active acylhydrobenzoquinones were a defining factor in the separation of one of the groups of plants while another cluster in both *Plectranthus s.s*. and *Coleus* separated based on flavonoid contents. It would be of interest to examine how this trend might be broadened to encompass all species within each genus.

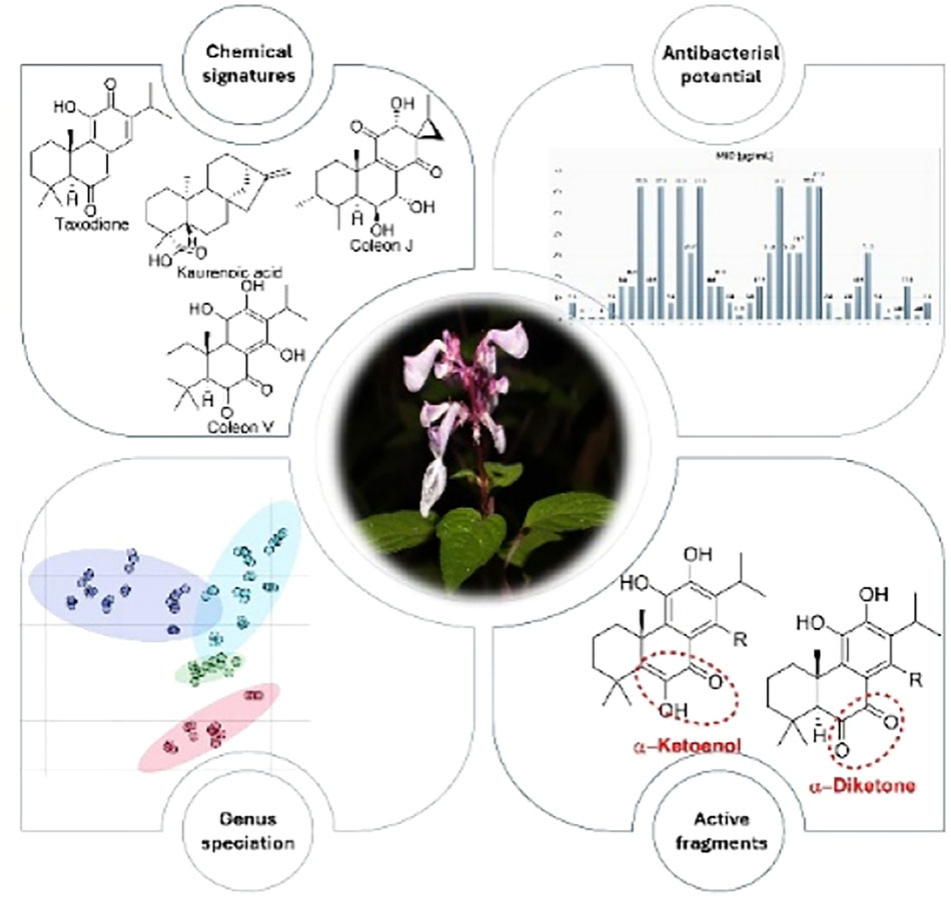

## Introduction

*Plectranthus* and *Coleus* (family Lamiaceae) are two genera of plants that have attracted considerable attention in recent decades because of their pivotal position in the systematic understanding of the Lamiaceae, their chemistry and traditional uses [1, 2]. Historically the allocation of plants to these genera has been unclear. Molecular based data have enabled greater clarity as to which species should be placed into which genera [3]. The molecular data has placed species from the larger genus *Plectranthus sensu lato* into three subsequent clades *Coleus*, *Equilabium* and *Plectranthus sensu stricto* [3]. The genus *Coleus* contains about 294 species while *Plectranthus s.s* (or simply *Plectranthus*) contains 72 species [3]. The latter is still part of the Plectranthinae clade together with *Aoellanthus*, *Tetradenia*, *Ocimum*, and *Alvesia* while *Coleus* constitutes a sister clade to Plectranthinae [4].

Species of *Plectranthus* and *Coleus* differ in the types of compounds they metabolize. Abietane diterpenoids are the marker molecules of both genera although they also produce other types of diterpenoids such as labdanes, kauranes or pimaranes. In the latest classification by Grayer et al., abietanes of *Plectranthus s.l* genus are clustered into 14-deoxy phenolic abietanes, acylhydrobenzoquinones, royleanones, spirocoleons, 14-deoxy-*p*-quinomethane and C-14-oxygenated *p*-quinomethane [1]. Coleus species tend to produce three of the five classes of abietanes, the exception being the 14-deoxy phenolic abietanes and 14-deoxy-*p*-quinomethanes which occur in *Plectranthus s.s*. [1]. It is, however, not surprising to observe some acylhydrobenzoquinones in *Plectranthus s.s*, as they might originate from non-enzymatic processes, but having the other classes in a *Plectranthus* species has not been reported [1, 2]. Thus, *Plectranthus s.s* distances itself from *Coleus* based on the oxidation of the C-14 position of their abietanes [1].

Species from both genera have many applications. Their leaves have economic value as ornamentals because of their vibrant colours and, leaves and flowers are used in decorative displays [5]. The pleasant aromas given off by their leaves and flowers are attributed to monoterpenes and sesquiterpenes in their fragrances [5]. In traditional medicine, species of *Plectranthus s.s* and *Coleus* are used to treat a range of ailments in different parts of the world [5]. Many of these species exhibit activity against bacteria [2]. This activity has been attributed in part to abietane diterpenoids as they have been shown to have antibacterial activity [2].

For instance, coleon U and its quinone, as well as coleon A and its lactone, have demonstrated minimal inhibitory concentration (MIC) values of 3.2 and 25 *µ*g/mL, respectively, against *Bacillus subtilis* [6]. Similarly, 7*β*,6*β*-dihydroxyroyleanone, 7*β*-acetoxy-6*β*-hydroxyroyleanone, and coleon U quinone have inhibited both *Staphylococcus aureus* and *Enterococcus faecalis* with MICs ranging from 0.5 to 32 *µ*g/mL [7]. This activity remains predominantly constrained to Gram-positive bacteria. This is the case even for other abietanes isolated from various other genera within the Lamiaceae family [8]. It is proposed that the antibacterial action of abietanes involves the disruption of microbial cell walls. However, they appear not to be active against Gram negative bacteria. This could be because they are not able to disrupt and/or penetrate the peptidoglycan cell wall and the outer lipopolysaccharide membrane that surround Gram negative bacteria cells.

The diversity of abietane-type compounds in *Plectranthus s.l*. that are active against Gram-positive bacteria remains poorly studied. Similarly, it is unclear whether the chemical differences between *Plectranthus s.s*. and *Coleus* significantly influence their antibacterial properties. The present study analyses a subset of species from *Plectranthus s.s*. and *Coleus* genera to reveal the weight of different classes of abietanes in the considerable antibacterial activity of some of their plant extracts.

## Results and discussion

### Extract profiling and identification of active compounds

None of the extracts made from the 10 species collected in 1994 (Table 1) exhibited activity against the Gram-negative (*E. coli*) or fungal strains. However, both the hexane and EtOAc extracts demonstrated mild to strong activity against *S. aureus*, with MIC values ranging from 500 *µ*g/mL to 7.8 *µ*g/mL (Table S1). In contrast, the MeOH extracts showed minimal to no activity against any of the three strains. Based on LC-MS comparison, hexane extracts often contained subsets of compounds present in the EtOAc extracts for each species. Consequently, the EtOAc extracts were further fractionated using flash chromatography to obtain fractions that contained fewer compounds. These fractions were collected, dried, and dissolved in CDCl₃ for NMR analysis, including both 1D and 2D experiments. The analysis revealed the presence, across the species, of a diverse range of compounds from all six classes of abietanes described in *Plectranthus s.l*. by Grayer et al. [1].

**Table 1:** Information on the aerial parts of species of *Coleus* (23 species) and *Plectranthus s.s*. (7 species) studied

BI Number	Accession Number	Most used name (synonym)	Accepted name	Date of collection
1671^a^	1970-2059	*Plectranthus crassus* N.E.Br.	*Coleus crassus* N.E.Br. Culham	9/1994
1829^a^	1970-4296	*Coleus kilimandschari* (Gürke) H.I.Maass	*Coleus barbatus var. grandis* (L.H.Cramer) A.J.Paton	9/1994
33218^a^	1990-1148	*Plectranthus fruticosus* (Wight ex Benth.) Hook.f.	*Coleus fruticosus* Wight ex Benth.	9/1994
33220^a^	1970-3770	*P. tetradenifolius* A.J.Paton	*Coleus tetradenifolius* (A.J.Paton) A.J.Paton	9/1994
33221^a^	1988-5028	*Plectranthus elegans* Britten	*Plectranthus elegans* Britten	9/1994
33222^a^		*Plectranthus ecklonii* Benth.	*Plectranthus ecklonii* Benth.	9/1994
33224^a^	1970-3783	*Plectranthus grandis* (L.H.Cramer) R.H.Willemse	*Coleus barbatus var grandis* (L.H.Cramer) A.J.Paton	9/1994
33225^a^	1977-3287	*Plectranthus hadiensis* (Forssk.) Schweinf. ex Sprenger	*Coleus hadiensis* (Forssk.) A.J.Paton	9/1994
33226^a^	1977-1638	*Plectranthus hyemalis* J.R.I.Wood	*Coleus hymalis* (J.R.I.Wood) A.J.Paton	9/1994
33227^a^	1959-79901	*Plectranthus coleoides* Benth.	*Coleus paniculatus* Benth.	9/1994
33903	1978-1972	*Plectranthus asirensis* J.R.I. Wood	*Coleus arabicus* Benth.	20/11/2023
33904	1992-1534	*Plectranthus barbatus var. barbatus* Andrews	*Coleus barbatus* (Andrews) Benth. ex G.Don	20/11/2023
33905	1980-2707	*Plectranthus cicatricosus* Chiov.	*Coleus burorum* Chiov.	20/11/2023
33906	1993-3100	*Plectranthus aegyptiacus* (Forssk.) C.Chr.	*Coleus aegyptiacus* (Forssk.) A.J.Paton	20/11/2023
33908	1998-2733	*Plectranthus ecklonii* Benth.	*Plectranthus ecklonii* Benth.	20/11/2023
33909	1989-2524	*Plectranthus ernstii* Codd	*Plectranthus ernstii* Codd	20/11/2023
33910	1977-406	*Plectranthus hadiensis* (Forssk.) Schweinf. ex Sprenger	*Coleus hadiensis* (Forssk.) A.J.Paton	20/11/2023
33911	1977-1639	*Plectranthus hyemalis* J.R.I.Wood	*Coleus hymalis* (J.R.I.Wood) A.J.Paton	20/11/2023
33912	1973-1207	*Plectranthus lanuginosus* (Hochst. ex Benth.) Agnew	*Coleus lanuginosus* Hochst. ex Benth.	20/11/2023
33913	1996-912	*Plectranthus montanus* Benth.	*Coleus cylindraceus* (Hochst. ex Benth.) A.J.Paton	20/11/2023
33914	1955-47101	*Plectranthus mutabilis* Codd	*Coleus mutabilis* (Codd) A.J.Paton	20/11/2023
33917	1989-1322	*Plectranthus xerophilus* Codd	*Coleus xerophilus* (Codd) A.J.Paton	20/11/2023
33918	1993-3205	*Plectranthus tenuiflorus* (Vatke) Agnew	*Coleus aegyptiacus* (Forssk.) A.J.Paton	20/11/2023
33919	1970-3550	*Plectranthus shirensis* (Gürke) A.J.Paton	*Coleus shirensis* Gürke	20/11/2023
33920	1999-13	*Plectranthus saccatus var. longitubus* Codd	*Plectranthus saccatus var. longitubus* Codd	20/11/2023
33922	1993-3264	*Plectranthus puberulentus* J.K.Morton	*Coleus gracilis* Gürke	20/11/2023
33923	1970-4305	*Plectranthus pseudomarrubioides* R.H.Willemse	*Coleus succulentus* Pax	20/11/2023
33924	1996-2730	*Plectranthus saccatus subsp. saccatus*	*Plectranthus saccatus subsp. saccatus*	20/11/2023

^a^Plants used for the chemical profiling

**Fig. 1:**
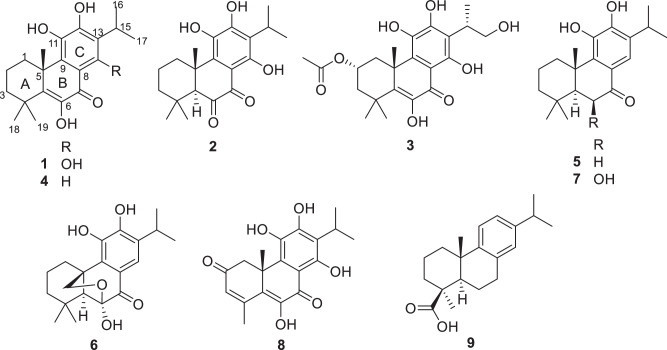
Major phenolic abietanes (**1**-**3,** **8**) and acylhydrobenzoquinones (**4**-**7,** **9**) of the studied species

Phenolic abietanes were the most common type of abietane identified in *Coleus hadiensis*, *C. tetradenifolius*, *C. paniculatus*, *C. crassus*, *C. kilimandschari*, *C. fruticosus*, *C. barbatus var. grandis*, *C. hymalis*, and *Plectranthus elegans*. In contrast, acylhydrobenzoquinone abietanes were only detected in *P. elegans*, *C. barbatus var. grandis*, *C. hadiensis*, *C. hymalis*, *C. paniculatus*, and *C. crassus*. Coleon U (**1**) emerged as the most widespread acylhydrobenzoquinone abietane, identified in *C. hadiensis*, *C. paniculatus*, *C. crassus*, and *C. tetradenifolius*, followed by coleon V (**2**), which was found in *C. paniculatus* and *C. crassus*. *C. barbatus var. grandis* and *C. hymalis* shared a representative of this group, namely 2-acetyloxy-16-hydroxycoleon U (**3**) (Fig. 1). Among the phenolic abietanes, 14-deoxycoleon U (**4**) and demethylcryptojaponol (**5**) were the most frequently encountered compounds, appearing in *C. hadiensis* and *P. elegans*. Other notable phenolic abietanes included carnosolon (**6**), detected in *C. hadiensis* and *C. tetradenifolius*, and 6*α*-hydroxydemethylcryptojaponol (**7**), present in *C. hadiensis* and *P. elegans*. Interestingly, all representatives of both phenolic and acylhydrobenzoquinone abietanes contained a carbonyl group at C-7, including coleon B (**8**) and a novel analogue found in *C. kilimandschari*. Some species, such as *C. hadiensis*, displayed both 14-oxy and 14-deoxy phenolic abietanes. Additionally, some phenolic abietanes in *C. tetradenifolius* featured a carboxylic group at C-18, such as callitrisic acid (**9**) (Fig. 1).

**Fig. 2:**
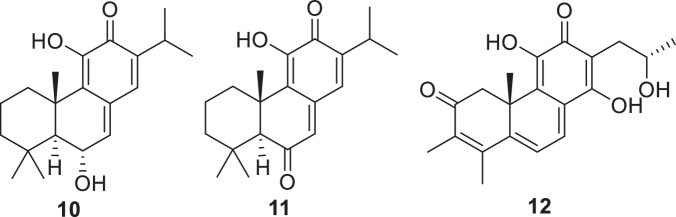
Major 14-deoxy-*p*-quinomethane (**10**-**11**) and C-14-oxygenated *p*-quinomethane (**12**) found in both *P. elegans* and *C. barbatus var. grandis*, respectively

Moreover, *p*-quinomethanes or *p*-methylenequinones, another group of abietanes, (Fig. 2) were identified in *P. elegans*, including taxodone (**10**) and taxodione (**11**), and in *C. barbatus var. grandis*, represented by coleon E (**12**). The first two compounds, characterized by a 14-deoxy skeleton, were found in a *Plectranthus* species, while compound **12**, which features a hydroxyl group at C-14 on the abietane backbone, was reported in a *Coleus* species, as highlighted by Grayer et al. [1].

**Fig. 3:**
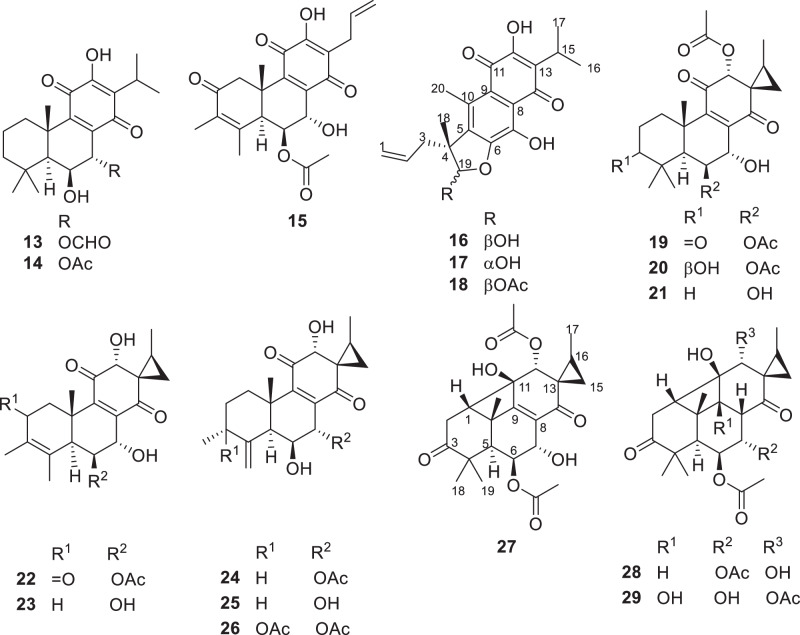
Major royleanones (**13**-**18**) and spirocoleons (**19**-**29**) identified

Royleanones, one of the principal compounds found in *Plectranthus* and *Coleus*, were identified in *C. paniculatus*, *C. fruticosus*, *C. kilimandschari*, *C. crassus*, *C. barbatus var. grandis*, and *C. hymalis*. *C. paniculatus* and *C. crassus* contained only two representatives of this group: 7*α*-formyloxy-6*β*-hydroxyroyleanone (**13**) and 7*α*-acetoxy-6*β*-hydroxyroyleanone (**14**) (Fig. 3). In *C. fruticosus*, three analogues were identified with structural features including a rearranged ring A forming a dimethylcyclohex-2-enone and an allylic side chain on the quinonoid ring C, similar to allylroyleanone (**15**) in *C. barbatus var grandis* or a second novel compound found in *C. hymalis* (Fig. 3). In *C. kilimandschari*, ring A was rearranged into a methylallyldihydrofuran fused with a phenyl group, represented by both epimers at C-19 of coleon A, (4*R*,19*R*)- (**16**) and (4*R*,19*S*)-coleon A (**17**), along with the 19-*O*-acetyl derivative of (4*R*,19*R*)-coleon A (**18**), new to science (Fig. 3).

The core structure of royleanones was found in both *C. barbatus var grandis* and *C. hymalis*, comprising mainly spirocoleons or 13,16-cycloabietanes, which were mono-, di-, or triacetylated and classified into three subclasses (Fig. 3). The first subclass included spirocoleons *sensu stricto*, such as barbatusin (**19**), 3*β*-hydroxy-3-deoxybarbatusin (**20**) and coleon P (**21**). The second subclass featured analogues with a spiroquinonoid ring C and a rearranged ring A, forming dimethylcyclohex-2-enone or dimethylcyclohexene structures, as seen in plectrin (**22**) from *C. barbatus var grandis* and coleon T (**23**) from *C. hymalis*. In some cases, the *endo*-olefin in the rearranged ring A underwent further rearrangement to form an *exo*-olefin group at C-4, as observed in coleons G (**24**), J (**25**), and 3-acetyloxycoleon G (**26**) from *C. hymalis*. This latter spirocoleon-type was exclusive to *C. hymalis*, absent from *C. barbatus var grandis* or any other species examined. The third subclass consisted of analogues with an unusual four-membered ring formed by a C-1/C-11 coupling from barbatusin (**19**). This type of abietane was omitted in the latest classification by Grayer et al.^1^. Several examples of this class were identified in fractions of *C. hymalis*, including dehydroxycyclobutatusin (**27**), 7*β*-acetyl-12-desacetoxycyclobutatusin (**28**), cyclobutatusin (**29**) and two derivatives of compound **29**, yet to be isolated and fully characterized.

**Fig. 4:**
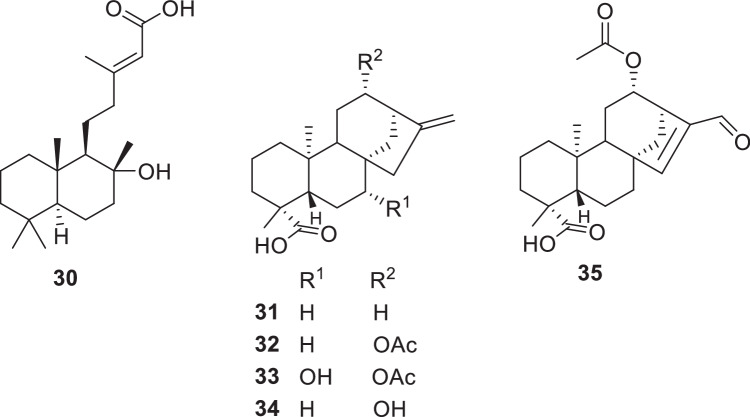
Major labdane (**30**) and *n*-kaurane (**31**-**35**) derivatives in *C. barbatus var grandis* and *C. fruticosus*

Besides abietanes, two other groups of diterpenes (Fig. 4) were identified across the species. Two labdanes derivatives were identified in *C. barbatus var grandis*, labd-13-en-8-ol-15-oic acid (**30**) together with its 12-acetyloxy derivative not reported in the literature. Similar labdanes have been previously documented in *P. ornatus*, now classified as *C. comosus* [9, 10]. On the other hand, *C. fruticosus* contained a significant number of kauren-18-oic acid derivatives including kaurenoic acid (**31**), 12-acetyloxykaurenoic acid (**32**), 12-acetyloxy-7-hydroxykaurenoic acid (**33**), 12-hydroxykaurenoic acid (**34**), 12-acetoxy-17-oxokaur-15-en-19-oic acid (**35**) and some derivatives not yet reported in the literature. Kauranes have already been reported in the literature from *P. ambiguous* [11] and *P. saccatus* [6]. Gaspar-Marques et al. also documented approximately 15 kaurenoic acid derivatives from *P. fruticosus* (now *C. fruticosus*) [12].

### Antimicrobial activity and structure-activity relationship

**Fig. 5:**
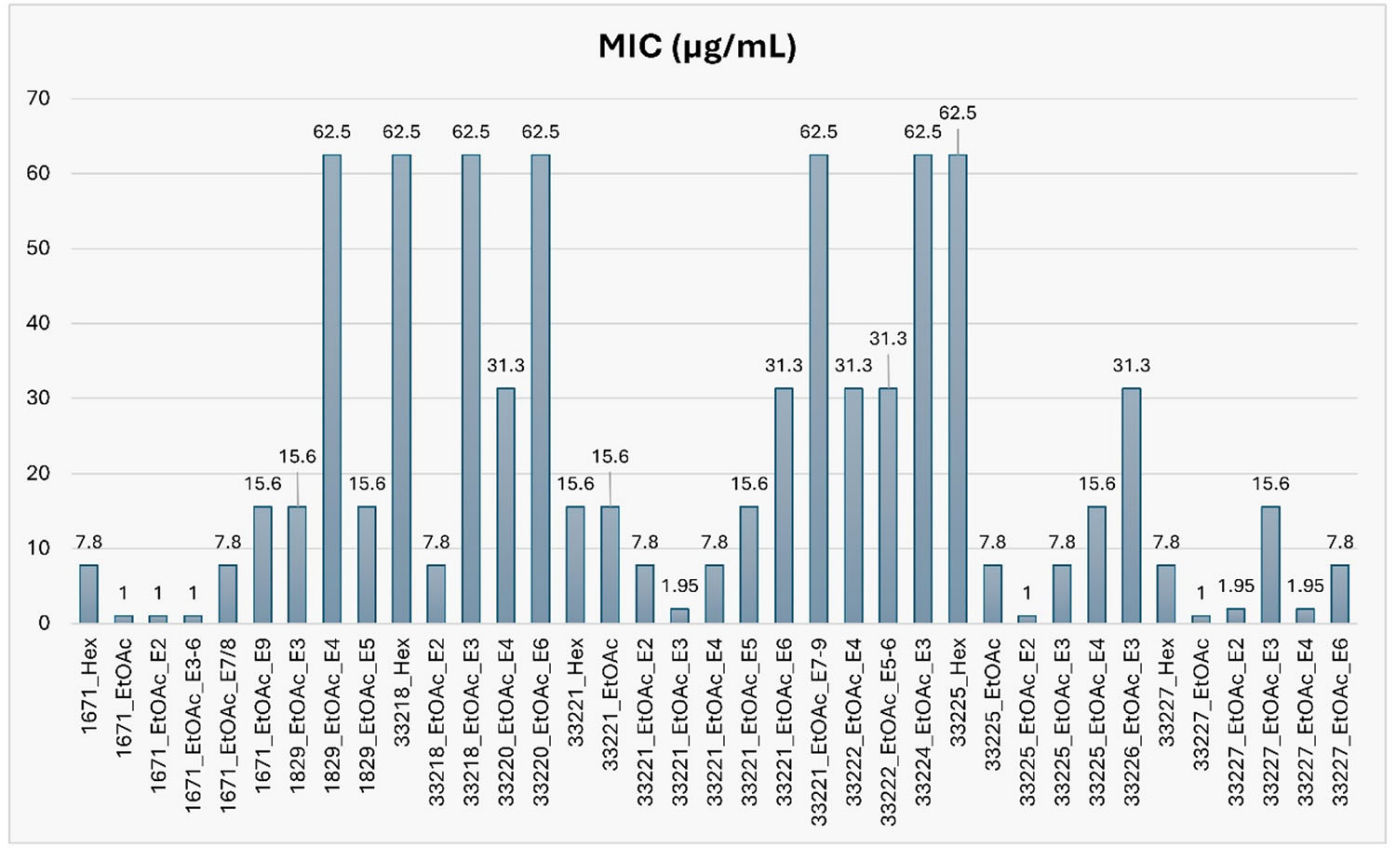
Antibacterial potency of extracts and fractions of the ten species against *S. aureus*. Samples are labelled with accession numbers of each plant as stated in Table 1, followed by the extract type (Hex for hexane and EtOAc for ethyl acetate extracts) and fraction numbers as obtained from the flash chromatography fractionation of the EtOAc extracts. MIC of piroctone olamine (positive control) = 15.6 *µ*g/mL. N = 3

The flash chromatography of the 10 EtOAc extracts from the 10 species generated 59 fractions. All were tested for activity against the three microbial strains. Again, none of them showed activity to *E. coli* and *A. brasilliensis*. Instead, they demonstrated activity against *S. aureus* with MIC values ranging from <1.95 to 500 *µ*g/mL. Among them, 32 fractions (54%) gave MIC values ≤ 62.5 *µ*g/mL (Fig. 5). Some of the fractions exhibited very potent activity, MIC ≤ 7.8 *µ*g/mL. The potency of the extracts, mainly *C. crassus* (MIC < 1.95 *µ*g/mL), *C. hadiensis* (MIC 7.8 *µ*g/mL) and *C. paniculatus* (MIC < 1.95 *µ*g/mL), was, when fractionated, successfully concentrated in five of the fractions of *C. crassus* (1671_EtOAc_E2-6), two of *C. paniculatus* (33227_EtOAc_E2 and E4) and one of either *P. elegans* (33221_EtOAc_E3) or *C. hadiensis* (33225_EtOAc_E2), each of these fractions had a MIC 1.95 *µ*g/mL or lower.

The NMR chemical profiling of the primary constituents in these fractions revealed a clear correlation between specific coleons and their antibacterial activity. For instance, fraction 1671_EtOAc_E2 was found to contain a mixture of at least coleon U (**1**), and 7*α*-acetyloxy-6*β*-hydroxyroyleanone (**14**), with coleon U (**1**) being the dominant compound. Subsequent fractions, 1671_EtOAc_E3 and 1671_EtOAc_E4, consisted almost exclusively of coleon V (**2**). Meanwhile, fractions 1671_EtOAc_E5 and 1671_EtOAc_E6 were composed of various proportions of coleons U (**1**) and V (**2**). Similarly, fraction 33221_EtOAc_E3 contained a mixture of 14-deoxycoleon U (**4**), demethylcryptojaponol (**5**), 6*α*-hydroxydemethylcryptojaponol (**7**) and taxodone (**10**), with 14-deoxycoleon U (**4**) identified as the major component. Furthermore, fraction 33225_EtOAc_E2, which exhibited a MIC value of <1.95 *µ*g/mL, was composed solely of coleon U (**1**). Fractions 33227_EtOAc_E4 and 33227_EtOAc_E2 demonstrated varied compositions. The former was primarily made up of coleon V (**2**) with smaller amounts of coleon U (**1**), while the latter had compound **1** as the major constituent alongside 7*α*-formyloxy-6*β*-hydroxyroyleanone (**13**) and 7*α*-acetoxy-6*β*-hydroxyroyleanone (**14**).

As a result, the fractions of *Coleus sp*. with the most activity contained coleons U (**1**) and V (**2**), whereas with *Plectranthus s.s. sp*. the most active fractions contained 14-deoxycoleon U (**4**). These three compounds share a distinguishing feature: an *α*-diketone or *α*-ketoenol group located in the B-ring, setting them apart from other abietanes. This structural motif was also observed in 2-acetyloxy-16-hydroxycoleon U (**3**) and coleon B (**8**). Fraction 1829_EtOAc_E5, which contained coleon B (**8**) as its sole component, and fraction 33224_EtOAc_E3, where 2-acetyloxy-16-hydroxycoleon U (**3**) was a minor component, exhibited considerable antibacterial activity with MIC values of 15.6 *µ*g/mL and 62.5 *µ*g/mL, respectively. These results suggest that oxidation on the A-ring and the isopropyl side chain negatively impact the activity of compounds containing either of the active *α*-diketone or *α*-ketoenol fragments. These fragments may account for the relatively strong activity observed for Coleon U and its quinone [6], coleon V [13] and 14-deoxycoleon U [14] particularly against Gram-positive bacteria. This is the first report on the antibacterial activity of coleon B.

### Distribution of coleons across *Plectranthus s.l*

A broader study of species of *Plectranthus* and *Coleus* growing at the Royal Botanic Gardens, Kew was then undertaken to evaluate whether other species in these genera could be sources of coleons with potency against *S. aureus* (Table 1). Eighteen additional plants were added to the analysis, from both *Coleus* and *Plectranthus s.s* genera. The 18 fresh samples of plants were collected in September 2023, whereas the first ten samples were collected 30 years ago in 1993-1994. This provided a unique opportunity to assess the stability of coleons over time by comparing the chemical profiles of plants from the same species but collected decades apart. In addition to the diterpenoids, flavonoids were consistently detected in nearly all of the ten previously collected species, as confirmed by NMR and LC-MS data. The distribution of these flavonoids in the additional species of *Plectranthus* and *Coleus* was also studied to see if they help in distinguishing these genera from other members of the Plectranthinae clade.

Peak area data obtained from both positive and negative ion modes of LC-MS analysis were used to evaluate key compounds or molecular markers that contribute the most to differentiating each species from the rest. A total of 232 peaks associated with different compounds were used in a Partial Least Squares Discriminant Analysis (PLS-DA). The heatmap (Fig. 5) of the top 200 most significant of them based on the analysis of their variance categorized the species into four groups: plants with a higher accumulation of active coleons (Group A); plants containing royleanones and analogues (Group B); plants containing a few other diterpene classes (Group C) and plants with a predominant accumulation of flavonoids (Group D).

**Fig. 6:**
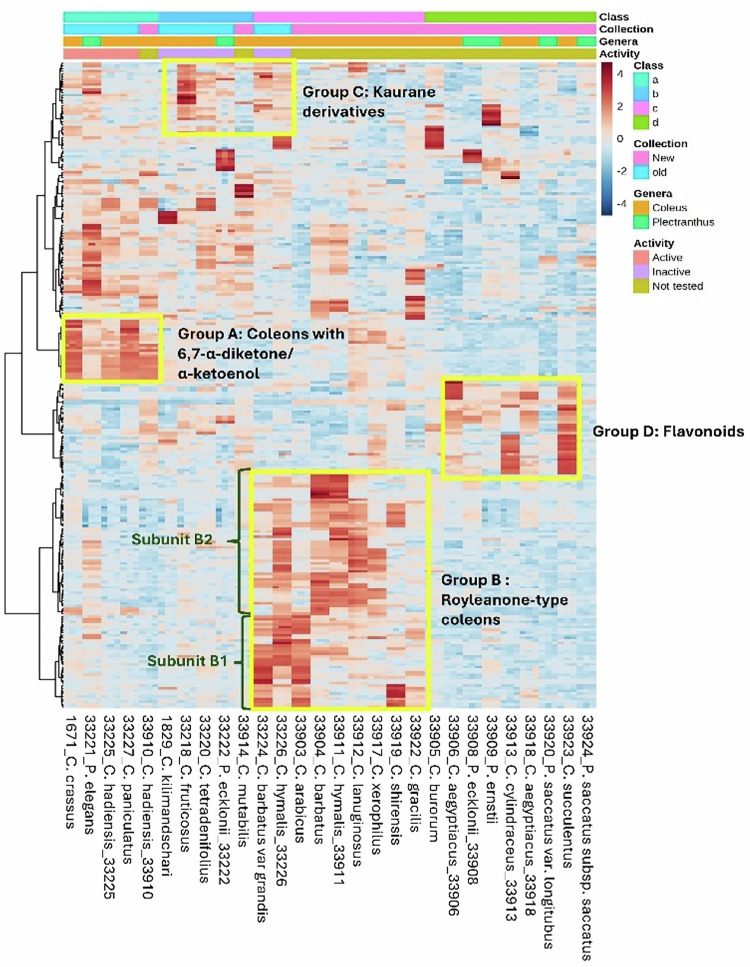
Heatmap constructed from a multifactorial analysis: class (a, b, c and d representing the four clusters defined from the principal component analysis (Fig. S1); collection (old or newly collected); genera (*Plectranthus s.s* or *Coleus*) and activity against *S. aureus* (active (MIC ≤ 62.5 *µ*g/mL), not active and not tested, the bioassay outcomes)

**Fig. 7:**
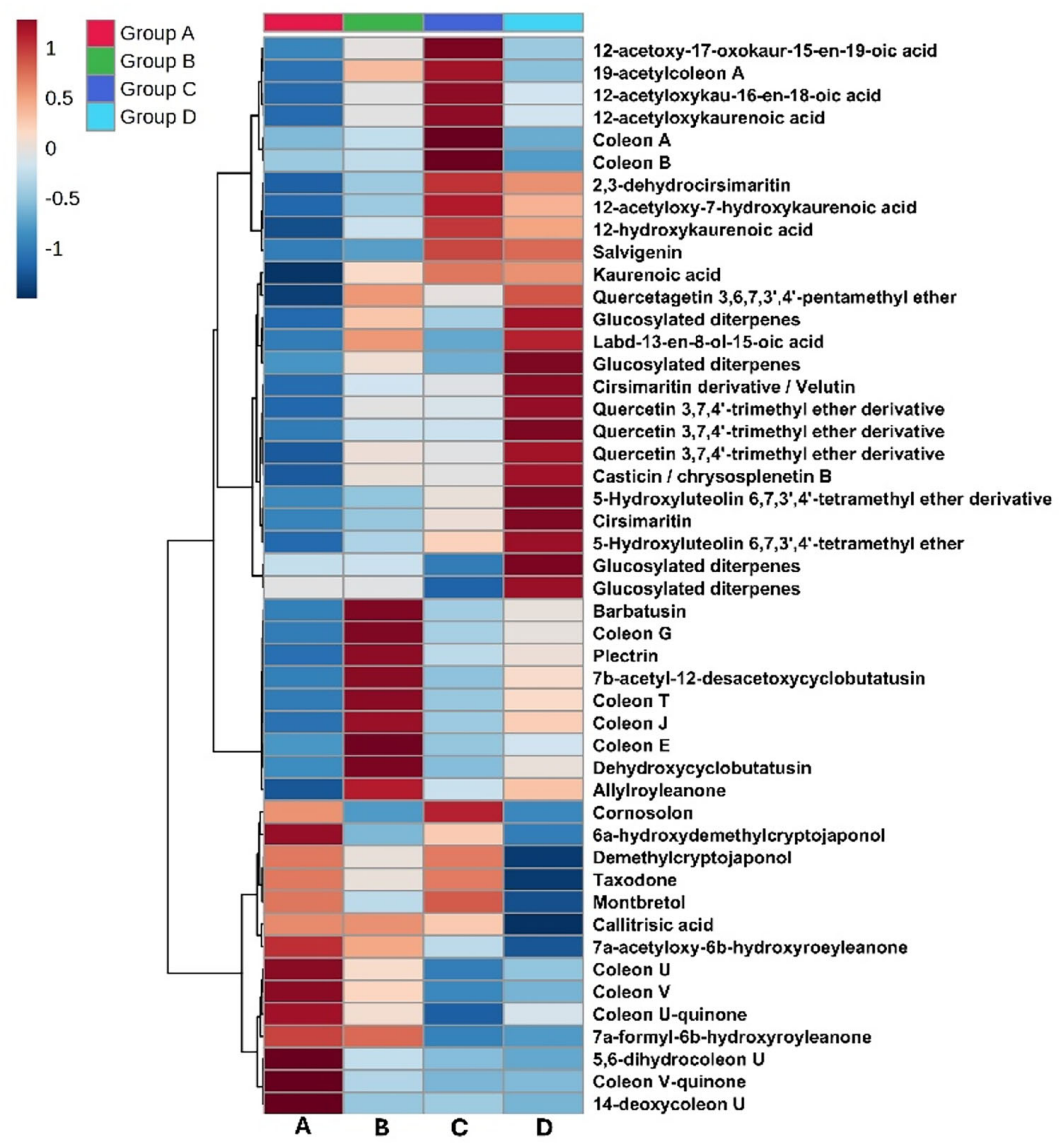
Distribution averaged of compounds identified during chemical profiling in Groups A-D

Group A (Figs. 6 and 7) consisted exclusively of species exhibiting the highest potency against *S. aureus*, as previously detailed. The recently collected *C. hadiensis_*33910 joined the group thus demonstrating its chemical similarity to the same species collected 30 years earlier. Data from the positive ionization mode also revealed the presence of quinone derivatives, specifically coleons U-/V-quinones (Table 2), in species that also contained their benzoquinone analogues. These species included *C. crassus*, *C. hadiensis*, *C. paniculatus*, and *C. hadiensis_33910*.

**Table 2:** LC-MS characteristics of selected compounds from the studied plants

Compounds	RT (min)	MS	Ion	Δ (ppm)	Formula	MS2	Ref^a^
Glucosylated diterpene	9.47	685.3071	[M + HCOOH-H]^−^	−0.9	C_33_H_49_O_15_	373.2009, **331.1915**, 313.1808, 287.2021, 205.0719, 163.0612, 143.0350, 115.0401, 101.0244, 89.0245	–
Glucosylated diterpene	9.72	671.3282	[M + HCOOH-H]^−^	−0.3	C_33_H_51_O_14_	317.2121, 205.0718, 163.0614, 143.0350, 115.0401, **101.0244**, 89.0244	–
Glucosylated diterpene	10.31	539.2490	[M + HCOOH-H]^−^	−1.5	C_27_H_39_O_11_	**331.1915**, 287.2021, 89.0244	–
Glucosylated diterpene	10.44	525.2699	[M + HCOOH-H]^−^	−1.2	C_27_H_41_O_10_	317.2117, 127.1120, **101.0246**, 71.0139, 59.0139	–
12-Acetoxy-17-oxokaur-15-en-19-oic acid (**35**)	11.75	373.2013	[M-H]^−^	−2.0	C_22_H_29_O_5_	373.2016, 313.1806, **59.0138**	[12]
2,3-Dihydrocirsimaritin	12.18	317.1017	[M + H]^+^	−0.8	C_17_H_17_O_6_	317.2112, 275.1642, 221.1172, 197.0445, 179.0703, **123.1169**	[20]
Plectrin (**22**)	12.23	401.1598	[M-H]^−^	−2.0	C_22_H_25_O_7_	340.1313, 323.1289, **308.1054**, 295.1340, 293.0820, 59.0138	[21]
Coleon J (**25**)	12.39	345.1701	[M-H]^−^	−2.0	C_20_H_25_O_5_	**327.1601**, 309.1495, 294.1260, 281.1183,	[22]
Cirsimaritin	12.50	313.0715	[M-H]^−^	−3.0	C_17_H_13_O_6_	298.0481, **283.0247**	[20]
Barbatusin (**19**)	12.68	445.1858	[M-H]^−^	−2.1	C_24_H_29_O_8_	343.1544, **325.1443**, 310.1209, 295.0974, 59.0138	[23]
Casticin/ chrysosplenetin B	13.10	375.1072	[M + H]^+^	−0.6	C_19_H_19_O_8_	**375.1076**, 360.0841, 359.0764, 345.0608, 342.0736, 327.0503, 325.0710, 317.0658, 314.0786, 311.0552, 299.0553	[24]
12-Hydroxykaurenoic acid **(34**)	13.16	317.2118	[M-H]^−^	−1.2	C_20_H_29_O_3_	**317.2118**	[12]
Casticin/ chrysosplenetin B	13.23	375.1071	[M + H]^+^	−0.9	C_19_H_19_O_8_	**375.1076**, 360.0841, 359.0764, 345.0608, 342.0736, 327.0503, 325.0710, 317.0658, 314.0786, 311.0552, 299.0553	[24]
Dehydroxycyclobutatusin (**27**)	13.30	445.1859	[M-H]^−^	−2.1	C_24_H_29_O_8_	367.1543, **325.1442**, 310.1208, 59.0138	[25]
7*β*-Acetyl-12-desacetoxycyclobutatusin (**28**)	13.30	447.2014	[M-H]^−^	−2.3	C_24_H_31_O_8_	**59.0138**	[23]
Allylroyleanone (**15**)	13.52	399.1458	[M-H]^−^	2.1	C_22_H_23_O_7_	**339.0811**, 234.0861	[26]
Unknown	13.89	359.1492	[M-H]^−^	−0.7	C_20_H_23_O_6_	359.1494, **344.1259**, 313.1445	–
Cyclobutatusin (**29**)	13.95	463.1964	[M-H]^−^	−2.1	C_24_H_31_O_9_	349.1435, 292.1103, **59.0138**	[23]
12-Acetyloxy-7-hydroxykaurenoic acid (**33**)	13.96	375.2167	[M-H]^−^	−2.6	C_22_H_31_O_5_	375.2171, **345.2066**, 315.1961, 285.1858	[12]
5-Hydroxyluteolin 6,7,3′,4′-tetramethyl ether derivative	13.98	359.1126	[M + H]^+^	0.1	C_19_H_19_O_7_	359.1128, 344.0891, **326.0787**, 315.0862, 298.0839	–
Cirsimaritin derivative / Velutin	13.98	315.0865	[M + H]^+^	0.7	C_17_H_15_O_6_	**315.0866**, 300.063, 272.0683	–
Quercetin 3,7,4′-trimethyl ether derivative	14.33	345.0967	[M + H]^+^	−0.5	C_18_H_17_O_7_	345.0971, **330.0737**, 315.0502	–
Quercetin 3,7,4′-trimethyl ether	14.47	345.0968	[M + H]^+^	−0.2	C_18_H_17_O_7_	345.0971, **330.0735**, 315.0501	–
Quercetagetin 3,6,7,3′,4′-pentamethyl ether	14.59	389.1229	[M + H]^+^	−0.4	C_20_H_21_O_8_	**389.1234**, 373.0921, 359.0765, 356.0893, 341.0659, 331.0817, 325.0710, 313.0710	–
Coleon E (**12**)	15.01	341.1399	[M-H]^−^	1.2	C_20_H_21_O_5_	**326.1360**	[27]
Salvigenin	15.13	329.1019	[M + H]^+^	−0.3	C_18_H_17_O_6_	329.1022, 314.0787, **296.068**, 268.0731	[28]
Unknown	15.19	419.1703	[M-H]^−^	−2.0	C_22_H_27_O_8_	419.1708, **359.1495**, 344.1261, 316.1315,	–
Coleon B (**8**)	15.43	343.1177	[M-H]^−^	−1.4	C_19_H_19_O_6_	343.1182, **328.0950**	[29]
Coleon G (**24**)	15.46	387.1807	[M-H]^−^	−1.5	C_22_H_27_O_6_	**59.0138**	[22]
Coleon T (**23**)	15.56	345.1698	[M-H]^−^	−2.6	C_20_H_25_O_5_	**327.1597**, 297.1493	[30]
5-Hydroxyluteolin 6,7,3′,4′-tetramethyl ether	15.82	359.1124	[M + H]^+^	−0.2	C_19_H_19_O_7_	359.1128, **344.0891**, 329.0658, 326.0787, 301.0709, 295.0602	–
12-Acetyloxykaurenoic acid (**32**)	15.83	359.2218	[M-H]^−^	−2.7	C_22_H_31_O_4_	**359.2219**, 299.2013	[12]
Montbretol or taxodione (**11**)	16.18	313.1807	[M-H]^−^	−0.8	C_20_H_25_O_3_	**313.1808**, 298.1573, 270.126, 255.1026, 217.0870,	[31]
Carnosolon (**6**)	16.39	345.1698	[M-H]^−^	−2.7	C_20_H_25_O_5_	345.1703, 327.1599, 302.1841, **283.1702**, 271.1703	[32]
2-Acetyloxy-16-hydrocoleon U	17.19	419.1703	[M-H]^−^	−2.0	C_22_H_27_O_8_	419.1708, **359.1495**, 344.1261, 316.1315,	–
Taxodone (**10**)	17.46	315.1960	[M-H]^−^	−1.9	C_20_H_27_O_3_	**315.1601**, 297.1495, 271.1705, 269.1548, 253.1597, 161.0609	[31]
5,6-Dihydrocoleon U	17.60	347.1854	[M-H]^−^	−3.0	C_20_H_27_O_5_	**347.1858**, 329.1757, 303.1599, 275.1651	[33]
Coleon U-quinone	17.72	345.1696	[M + H]^+^	−0.2	C_20_H_25_O_5_	317.1747, **299.1642**, 281.1537, 209.0809, 135.0805, 109.1012	–
6*α*-Hydroxydemethylcryptojaponol (**7**)	17.88	331.1910	[M-H]^−^	−1.5	C_20_H_27_O_4_	**331.1915**, 313.1809, 285.1861, 270.1627	[34]
Callitrisic acid (**9**)	17.92	301.2164	[M + H]^+^	0.6	C_20_H_29_O_2_	301.2161, **255.2108**, 199.1482, 147.1169, 133.1012	[35]
Coleon A (**16**/**17**)	18.17	357.1335	[M-H]^−^	−0.9	C_20_H_21_O_6_	357.1339, 316.0959299.0923, **288.1003**, 260.1053, 245.0818	[6]
7*α*-Formyloxy-6*β*-hydroxyroeyleanone (**13**)	18.27	375.1805	[M-H]^−^	−2.2	C_21_H_27_O_6_	347.1861, **329.1759**, 319.1915, 301.1809, 153.0558	[36]
Coleon U (**1**)	18.30	345.1697	[M-H]^−^	−3.4	C_20_H_25_O_5_	**345.1703**, 317.1757	[33]
14-Deoxycoleon U (**4**)	18.59	329.1754	[M-H]^−^	−1.4	C_20_H_25_O_4_	**329.1757**, 311.1652, 296.1417, 314.1523, 301.1808	[33]
Kaurenoic acid (**31**)	18.60	301.2171	[M-H]^−^	−0.8	C_20_H_29_O_2_	**301.2170**	[12]
7*α*-Acetyloxy-6*β*-hydroxyroeyleanone (**14**)	18.75	389.1960	[M-H]^−^	−2.4	C_22_H_29_O_6_	389.1964, **329.1758**, 301.1807, 285.1858	[37]
Labd-13-en-8-ol-15-oic acid (**30**)	19.17	321.2436	[M-H]^−^	0.2	C_20_H_21_O_5_	**277.2220**	[9, 10]
19-Acetylcoleon A (**18**)	19.74	399.1441	[M-H]^−^	−0.7	C_22_H_23_O_7_	**339.1233**, 324.1002, 311.1288	–
Coleon V (**2**)	20.32	345.1697	[M-H]^−^	−3.1	C_20_H_25_O_5_	**345.1703**, 317.1757	[33]
Coleon V-quinone	20.32	345.1698	[M + H]^+^	0.5	C_20_H_25_O_5_	317.1747, **299.1642**, 281.1537, 209.0809, 135.0805, 109.1012	–

Compounds identified during NMR profiling have disclosed identities

^a^NMR data of compounds were compared with data available in the lliterature as per the references

Base peak in MS2 spectrum is in bold

The list of plants from Group B (Figs. 6 and 7) shared some similarities with *C. barbatus var. grandis* and *C. hymalis*, mainly their profiles of royleanones. The fractionation of the EtOAc extracts of *C. barbatus var. grandis* and *C. hymalis*, done during chemical profiling, allows to track down some of the royleanones in the extracts by aligning the LC-MS of the fractions with that of the extracts. As shown in the heatmap (Figs. 6 and 7), the profiles in this group can be divided into two subunits (B1 and B2, Fig. 6). Subunit B1 (Fig. 6) contains royleanones like coleon E (**12**), 7*α*-acetoxy-6*β*-hydroxyroyleanone (**14**), allylroyleanone (**15**), barbatusin (**19**), plectrin (**22**) and coleon G (**24**) while coleons T (**23**) and J (**25**) were located in subunit B2 (Fig. 6). Some of the species in Group B share more of the compounds present in subunit B1 with *C. barbatus var. grandis*, such as *C. arabicus*, *C. shirensis*, *C. gracilis* and *C. hymalis* whereas the rest relates more to *C. hymalis* like *C. barbatus*, *C. hymalis*_33911, *C. lanuginosus*, *C. xerophilus* and *C. shirensis*. Therefore, *C. hymalis* and *C. shirensis* are the only species within group B to share similarities with both subunits. Indeed, the royleanones with modified A rings were only found in *C. hymalis* and not in *C. barbatus var. grandis*. These might constitute the core of the royleanones in *C. barbatus*, *C. hymalis*_33911, *C. lanuginosus* and *C. xerophilus* as they are only related to *C. hymalis*. As expected, the occurrence of royleanones was exclusive to *Coleus sp*.

The species of Group C like *C. fruticosus*, *C. kilimandschari*, *C. tetradenifolius*, *P. ecklonii*_33222 and *C. mutabilis* were characterized by the presence of either diterpene acids derived from kauranes and abietanes or abietanes with rearranged ring A in their phenolic and quinonoid forms. As shown on the heatmaps (Figs. 6 and 7), these diterpene acids seem to spread across Groups A and B of plants making them the second most abundant group of compounds after abietanes in *Plectranthus* and *Coleus*. Interestingly, based on current knowledge, plants within the Plectranthinae clade tend to produce diterpenes in pairs. *Aeollanthus* and *Tetradenia* for instance produce both isopimaranes and abietanes [15, 16]. Further investigations on the types of diterpenoids in these genera might identify compounds that could clearly distinguish between species of *Plectranthus* and *Coleus*.

Group D (Figs. 6 and 7) correlated strongly with flavonoid content with some species like *C. succulentus*, *C. cylindraceus*_33913 and *C. aegyptiacus*_33906. The occurrence of specific flavonoids was not restricted to a genus, although the concentration of flavonoids was higher in species of *Coleus* compared to species of *Plectranthus* (Fig. 5). Three flavonoids, salvigenin, cirsimaritin and cirsiliol were noticed to occur in all of the ten species profiled by NMR. They were also detected in the other 18 species chemically profiled by LC-MS (Table 1). They could be one of the markers of the Plectranthinae clade as the same compounds have already been reported to occur in species of *Aeollanthus* which also belong to the same clade [15]. Both *P. saccatus var. longitubus* and *P. saccatus subsp. saccatus* also clustered with Group D but the profiles, although quite similar to each other, did not show a clear affiliation to any of the Groups A-C. Other species in Group D also expressed the occurrences of glycosylated diterpenoids like *P. ernstii* where the negative mode HR-ESI-MS of the extract exhibited formate adduct ions [M + HCOOH-H]^–^ at *m/z* 685.3071 (C_33_H_49_O_15_), 671.3281 (C_33_H_51_O_14_), 539.2490 (C_27_H_39_O_11_) and 525.2699 (C_27_H_41_O_10_) (Table 2 and Fig. S11). This is quite important as no glucosylated diterpenoids were detected in any of the ten extracts initially studied, even from their 60% MeOH extracts, examined during the chemical profiling by NMR. The diterpene classes in these glycosylated derivatives are possibly with pimarane and labdane backbones as some analogues have been reported from *P. ernstii* (33909) [17, 18].

## Conclusions

Overall, the study reveals that *α*-ketoenol and *α*-diketone fragments of abietanes confer significant activity against *S. aureus*. Interestingly, these fragments were primarily found in acylhydrobenzoquinone and phenolic abietanes and barely in royleanones. Consequently, the difference in the oxidation of the C-14 carbon, which distinguishes the abietanes of *Plectranthus s.s*. from those of *Coleus*, does not influence the activity of these pharmacophores. This was clearly shown by the activity of coleon U (**1**), found in *Coleus*, compared to that of 14-deoxycoleon U (**3**), encountered in *Plectranthus*. These compounds appear to be chemically stable over time, as evidenced by the clustering of the new and old samples of *C. hadiensis* within the same Group A. Moreover, abietane classes in *Plectranthus* could constitute a key factor in the genera speciation. Species that produce acylhydrobenzoqionones and phenolic abietanes were distinct from those eliciting royleanones. Interestingly, the results also enforce flavonoids as another key factor in the classification of species within either *Coleus* or *Plectranthus s.s*. It would be intriguing to explore how this trend could be extended to encompass the entire species within each of the genera.

## Materials and methods

### General experimental procedure

LC–MS grade solvents (acetonitrile, methanol) and formic acid were obtained from Fisher Scientific (Loughborough, UK) and milliQ water was used for HPLC and LC-MS analysis. NMR spectra were acquired on a Bruker Avance-III (^1^H NMR: 400 MHz and ^13^C NMR: 100.1 MHz) spectrometer equipped with a 5 mm cryoprobe. Chemical shifts were referenced to residual solvent signals and reported in parts per million (ppm). Spectra were processed using Bruker NMR academic Topspin software. Mass spectra were collected on a Orbitrap Exploris mass spectrometer, equipped with a Vanquish diode array detector (VH-D10) coupled to an Orbitrap Exploris 120 with a heated ESI source (Thermo Scientific, Germany), acquired in both negative and positive modes with a resolution of 60,000 over *m/z* 125–1800 under various acquisition parameters like source voltages, sheath gas, auxiliary gas, sweep gas and capillary temperature set to 2.5 kV (negative mode) and 3.5 kV (positive mode), 50 (arbitrary units), 10 (arbitrary units), 1 (arbitrary units) and 350 °C, respectively. Automatic MS–MS fragmentation was performed on top four ions of the TIC using an isolation width of *m/z* 2. High-energy C-trap dissociation with a normalized collision energy of 40 and an activation time of 0.1 ms was served to fragment ions. The MS unit is interfaced with a Vanquish Core UHPLC system, which includes a Vanquish diode array detector (VH-D10) operating at four wavelengths: 210 nm, 254 nm, 300 nm, and 366 nm. Samples are injected using an autosampler maintained at 30 °C, while the analytical column ACQUITY Premier CSH C18 1.7 µm, 2.1 × 150 mm (Waters Corporation, Milford, MA, USA) is kept at a constant temperature of 35 °C. The injection volume for each sample was 1 *µ*L at a flowrate of 200 *µ*L/min and the gradient was an increase of acetonitrile (B) in water (A) (0–5 min, 10% B; 5–20 min, 10 to 100% B, 20 to 30 min, 100% B and 30 to 35 min, 10% B). Collected data were inspected using Xcalibur v. 4.2.47 (Thermo Fisher Scientific). Chemical profiling of extracts was conducted on a Biotage® Isolera One system for splitting extracts into small fractions.

### Sample preparation

The leaves of 28 species of *Plectranthus* and *Coleus* species (Table 1) were collected from the living collection at RBG Kew and attributed a BI accession number when collected. Their identifications were confirmed by Kew botanist supervised by the Lamiaceae specialist Dr Alan Paton. They were collected fresh at the given date, freeze-dried, milled to fine powders, and kept in dark for further use. When the time of work arrived, a single sample per species was prepared, serially extracted in *n*-hexane and EtOAc, and analysed by LC-MS in in tree nonconsecutive runs. The extraction in each solvent was repeated twice on the same plant material starting with the less polar solvent, *n*-hexane. The same protocol was repeated when following up with EtOAc as the solvent. For each species, the milled plant material (100 mg) was suspended in 2 mL of the solvent, vortexed vigorously for 10 s, heated in a water bath kept at 50 °C for 10 min and centrifuged at 15,600 rpm for 10 min. Part of the supernatant (1.5 mL) was transferred to a clean flask and dried using a GeneVac concentrator (Genevac, Suffolk, UK). Resulted extracts were dissolved in 2 mL ACN, sonicated and spined at 15,600 rpm for 10 min, then 300 *µ*L were transferred into a glass autosampler vial for LC-MS analysis. The remaining solution of 1.2 mL each was dried out using a GeneVac concentrator (Genevac, Suffolk, UK) and resulted extracts were dissolved in CDCl_3_ for NMR analysis.

### Data processing

The LC-MS data generated were deconvoluted, aligned and extracted as peak areas using MS-Dial (https://systemsomicslab.github.io/compms/msdial/main.html). The processing of data was done using the mass tolerance, rt tolerance and the minimum peak height of 0.01 Da, 0.04 min and 10^6^, respectively. The other parameters remained the same as default settings and no database was added for peak annotation. Both negative and positive modes were retrieved separately. On the other hand, ^1^H NMR spectra of all samples, including replicates, were stacked together, aligned to Gottlieb et al. reference of chloroform, and binned using a bin width of 0.04 ppm under the average sum method [19]. The NMR spectra were processed using Mnova V. 14 software from Mestrelab (https://mestrelab.com/). The statistical analysis was carried out online, using available packages on MetaboAnalyst (https://www.metaboanalyst.ca/) platform. Each species of the dataset was considered as a unique class with three technical replicates from the three different LC-MS runs of the sole sample per species. For preprocessing steps, the data collected were not filtered but were normalized after a log transformed and scaling using Pareto function. The data were analyzed both by one factor and metadata statistical analysis tools of MetaboAnalyst. Four categorical factors constituted the metadata: the class, made from the clustering; the collection (old or newly collected); the genus (*Plectranthus s.s* or *Coleus*) and the bioassay outcomes (active, not active, not tested).

Partial Least Squares Discriminant Analysis (PLS-DA) was carried out in MetaboAnalyst as a refinement step to retain features of high-confidence discriminatory power across all species groups. Following the preprocessing steps, features with the most significant variable importance in projection (VIP) score, set here as VIP ≥ 5, among ions detected in negative then positive modes were retained. Thereafter, both feature pools were combined to form the working dataset. Principal Component Analysis (PCA) was applied to assess differences across species using PERMANOVA based on Euclidean distance calculations. Different clusters of samples observed after the PCA was defined as groups. Hierarchical clustering heatmaps were generated in MetaboAnalyst using Euclidean distance and Ward’s linkage method to identify chemical classes of compounds (from the profiling of extracts based on NMR metabolomics) that contribute to group separation. They can be generated with all replicates visible, as in Fig. 6, or with the tree replicates per sample averaged, as in Fig. 7. PLS-DA was finally used to confirm the identities of the most important compounds that differentiate a group.

### Large scale extraction and fractionation

The leaves of each of the ten plant species collected in 1993 and kept in bags (50-200 g in weight) were milled to fine powders and serially extracted in solvents with increasing-polarities starting with *n*-hexane, then EtOAc and 60% MeOH. Resulting extracts were screened for antimicrobial activity. Following results, active extracts were each split into small fractions on the Biotage Isolera One system using a stepwise gradient of EtOAc (B) in hexane (A) (3CV, 100% A; 3CV, 10% B, 3CV, 20% B, 3CV, 30% B; 3CV, 40% B, 3CV, 50% B and 3CV, 100% B). For hexane extracts fractioned, the gradient applied, on the same system, was a stepwise increase of isopropanol (B) in MeOH (A) (3CV, 5% B; 3CV, 10% B, 3CV, 20% B, 3CV, 30% B; 3CV, 40% B, 3CV, 50% B and 3CV, 100% B). None of the 60% MeOH extracts was fractioned. The hexane extracts fractionation were done on a SNAP Ultra C18 60 g cartridge from Biotage with a flowrate of 30 mL/min, whereas the EtOAc extract fractionation was achieved on various silica cartridge, also from Biotage in a ratio of 1 g of extract for 10 g of cartridge silica mass. The flowrates were usually defined automatically by the system. The elutions were collected into tubes of 20 mL each and into small fractions (BI Number_E1, 2, 3, …, n) reflecting the separation showed on the peak chromatogram of each run. The fractions were dried down on a Rotary evaporator, dissolved in CDCl_3_ and submitted to ^1^H NMR then to 2D NMR analysis for chemical profiling. Each NMR dataset was carefully inspected to identify patterns leading to the profiling of major, and sometimes minor constituents of the fractions as listed in Table 2. A solution of 1 mg/mL of each fraction was also prepared and analyzed by LC-MS for confirmation of the structures and annotation of the extracts.

### Antimicrobial assays

Minimum Inhibitory Concentration (MIC) assays were used to determine the minimum concentration of an active required to inhibit the growth of microorganisms. Compounds were evaluated against a range of organisms including the Gram-positive bacterium, *Staphylococcus aureus* ATCC 6538, and the fungus- *Aspergillus brasiliensis* ATCC 16404. Inocula were prepared in saline (bacteria) or saline with tween (fungi) and adjusted turbidometrically to a target concentration of 10^7^ -10^8^ CFU/mL. This inoculum solution was further diluted in Tryptone Soy broth (*S. aureus*) or Soya Dextrose broth (*A. brasiliensis*) to achieve a final inoculum level of approximately 10^5^ CFU/mL assay use. A stock 96-well plate of the extracts were prepared in dimethyl sulfoxide (DMSO) at a concentration of 20 mg/mL and serially diluted in DMSO. Piroctone olamine and DMSO were also tested as positive and negative controls, respectively. For each test plate, 5 *µ*L of each dilution (each well from the stock plate) was transferred to a new test plate and 195 *µ*L of inoculum in broth was added to each well. The *S. aureus* plates were incubated on an orbital sharker for 18 ± 2 h at 32.5 °C. The *A. brasiliensis* plates were inoculated at 20-25 °C for 5 days. After incubation, the plates were visually assessed and the MICs were determined as the most dilute well with reduced growth (~50%) compared to a growth control. To determine the Minimum Biocidal Concentration (MBC), 10 *µ*L from each well was pipetted onto neutralizing agar (Modified Letheen Agar with Tween) and incubated for 24 h at 32.5 °C (*S. aureus*) or 5 days at 20-25 °C for 5 days (*A. brasiliensis)*. The MBC was determined as the most dilute concentration with no growth visible growth (Table S1). Extracts and fractions with MIC values exceeding 62.5 *µ*g/mL against *S. aureus* were selected to plot Fig. 5.

## Supplementary information

Supplementary information

## Data Availability

The NMR and LC–MS raw data generated and/or analysed during the current study are available from the corresponding author (Gabin Bitchagno) on request.
