# Supplementary material for: Acylhydrobenzoquinones influence the susceptibility of Staphylococcus aureus towards abietanes and the speciation within Plectranthus sensus lato
Source: Med Chem Res. 2026 Jan 13;35(3):548–62. doi: 10.1007/s00044-026-03524-7 (PMC13368947; doi:10.1007/s00044-026-03524-7)
Supplement: Supplementary file 1 — Supplementary information [file 44_2026_3524_MOESM1_ESM.docx]

**Acylhydrobenzoquinones influence the susceptibility of *Staphylococcus aureus* towards abietanes and the speciation within *Plectranthus* *sensus lato***

Gabin Thierry M. Bitchagno^1^ *, Sohini S. Bhatia^2^, Scott Bintrim^2^, Paula Coates^3^, Debbie Mulligan^2^, Monique S.J. Simmonds^1^*

^1^Royal Botanic Gardens Kew, Richmond, London, TW9 3AE

^2^The Procter & Gamble Company, Mason Business Center, Mason, Ohio, USA

^3^The Procter & Gamble Company, 452 Basingstoke Rd, Reading RG2 0RX

***Corresponding Authors**

Gabin T.M. Bitchagno

Royal Botanic Gardens Kew

Jodrell Laboratory

TW9 3AE Richmond

London

email: [g.bitchagnombahbou@kew.org](mailto:g.bitchagnombahbou@kew.org),

Phone: +442083325374

Monique S.J. Simmonds

Royal Botanic Gardens Kew

Jodrell Laboratory

TW9 3AE Richmond

London

email: [m.simmonds@kew.org](mailto:m.simmonds@kew.org)

Phone: +442083325328

Table S1. Results of antimicrobial activity and chemical composition of the ten initial plant extracts and fractions

|  | *E. coli* | | *S. aureus* | | *A. brasilensis* | | Chemical composition |
| --- | --- | --- | --- | --- | --- | --- | --- |
|  | MIC | MBC | MIC | MBC | MIC | MBC |  |
| *Coleus fruticosus* |  |  |  |  |  |  |  |
| 33218_Hex | >1000 | - | 62.5 | 125 | >1000 | - |  |
| 33218_EtOAc | >1000 | - | 500 | >1000 | >1000 | - |  |
| 33218_E1 | >1000 | - | 7.8 | 15.6 | >1000 | - | Fatty acid |
| 33218_E2 | >1000 | - | 62.5 | 125 | >1000 | - | **31** |
| 33218_E3 | >1000 | - | 250 | 1000 | >1000 | - | **32** |
| 33218_E4 | >1000 | - | 125 | >1000 | >1000 | - | Complex mixture |
| 33218_E5 | >1000 | - | 125 | >1000 | >1000 | - | Complex mixture |
| 33218_E6 | >1000 | - | 250 | >1000 | >1000 | - | **33**-**35**, salvigenin |
| 33218_E7 | >1000 | - | 7.8 | 15.6 | >1000 | - | Complex mixture containing **33** |
| *Coleus tetradenifolius* |  |  |  |  |  |  |  |
| 33220_Hex | >1000 | - | 500 | 1000 | >1000 | - |  |
| 33220_EtOAc | >1000 | - | 250 | >1000 | >1000 | - |  |
| 33220_E1 | >1000 | - | >1000 | >1000 | >1000 | - | Fatty acid |
| 33220_E2-3 | >1000 | - | 125 | 250 | >1000 | - | Complex mixture containing **9** |
| 33220_E4 | >1000 | - | 31.3 | 62.5 | >1000 | - | **6** |
| 33220_E5 | >1000 | - | >1000 | >1000 | >1000 | - | Salvigenin and complex mixture |
| 33220_E6 | >1000 | - | 62.5 | 62.5 | >1000 | - | Complex mixture |
| *Plectranthus elegans* |  |  |  |  |  |  |  |
| 33221_Hex | >1000 | - | 15.6 | 125 | >1000 | - |  |
| 33221_EtOAc | >1000 | - | 15.6 | 125 | >1000 | - |  |
| 33221_E1 |  |  |  |  |  |  | **11** |
| 33221_E2 | >1000 | - | 7.8 | 62.5 | >1000 | - | **4**, **5**, **7**, **10** |
| 33221_E3 | >1000 | - | 1.95 | 15.6 | >1000 | - | **4**, **5**, **7**, **10** |
| 33221_E4 | >1000 | - | 7.8 | 15.6 | >1000 | - | **5**, **7**, **10** |
| 33221_E5 | >1000 | - | 15.6 | 31.3 | >1000 | - | Complex mixture |
| 33221_E6 | >1000 | - | 31.3 | 62.5 | >1000 | - | Complex mixture |
| 33221_E7 | >1000 | - | 62.5 | 125 | >1000 | - | Complex mixture |
| 33221_E8 | >1000 | - | 62.5 | 500 | >1000 | - | Complex mixture |
| 33221_E9 | >1000 | - | 62.5 | 1000 | >1000 | - | Complex mixture |
| *Plectranthus ecklonii* |  |  |  |  |  |  |  |
| 33222_Hex | >1000 | - | >1000 | >1000 | >1000 | - |  |
| 33222_EtOAc | >1000 | - | 125 | 250 | >1000 | - |  |
| 33222_E1 | >1000 | - | >1000 | >1000 | >1000 | - | Fatty acid |
| 33222_E2 | >1000 | - | >1000 | >1000 | >1000 | - | Eckloquinones A and B |
| 33222_E3 | >1000 | - | 125 | 1000 | >1000 | - | Complex mixture |
| 33222_E4 | >1000 | - | 31.3 | 62.5 | >1000 | - | 2,3-dihydrocirsimaritin |
| 33222_E5-6 | >1000 | - | 31.3 | 125 | >1000 | - | **5**, cirsimaritin, (Z)-2-(2,4-dihydroxy-2,6,6-trimethylcyclohexylidene) acetic acid |
| 33222_E7 | >1000 | - | 250 | >1000 | >1000 | - | Complex mixture |
| *Coleus hadiensis* |  |  |  |  |  |  |  |
| 33225_Hex | >1000 | - | 62.5 | 125 | >1000 | - |  |
| 33225_EtOAc | >1000 | - | 7.8 | 15.6 | >1000 | - |  |
| 33225_E1 |  |  |  |  |  |  | **1** |
| 33225_E2 | >1000 | - | <1.95 | 3.9 | >1000 | - | **1** |
| 33225_E3 | >1000 | - | 7.8 | 31.3 | >1000 | - | **1**, **4**, **5**, **6** |
| 33225_E4 | >1000 | - | 15.6 | 62.5 | >1000 | - | **6** and alkyl ferulate |
| 33225_E5 | >1000 | - | 125 | 500 | >1000 | - | Complex mixture |
| *Coleus barbatus var grandis* |  |  |  |  |  |  |  |
| 33224_Hex | >1000 | - | >1000 | >1000 | >1000 | - |  |
| 33224_EtOAc | >1000 | - | 500 | >1000 | >1000 | - |  |
| 33224_E1 | >1000 | - | >1000 | >1000 | >1000 | - | Fatty acid |
| 33224_E2 | >1000 | - | >1000 | >1000 | >1000 | - | Complex mixture |
| 33224_E3 | >1000 | - | 62.5 | 500 | >1000 | - | **3**, **29** and a labdane |
| 33224_E4 | >1000 | - | 500 | 1000 | >1000 | - | **12**, **15**, **19**, **27**, **28**, **30** |
| 33224_E5 | >1000 | - | 250 | 1000 | >1000 | - | **19**, **20**, **22**, **27** |
| 33224_E6 | >1000 | - | 250 | 1000 | >1000 | - | Complex mixture |
| *Coleus hymalis* |  |  |  |  |  |  |  |
| 33226_Hex | >1000 | - | 500 | >1000 | >1000 | - |  |
| 33226_EtOAc | >1000 | - | 125 | 250 | >1000 | - |  |
| 33226_E1 | >1000 | - | >1000 |  | >1000 | - | Fatty acid |
| 33226_E2 | >1000 | - | 250 | >1000 | >1000 | - | Complex mixture |
| 33226_E3 | >1000 | - | 31.3 | 250 | >1000 | - | **24** |
| 33226_E4 | >1000 | - | 250 | 1000 | >1000 | - | **19**, **23**, **25**, **26**, **27**, |
| 33226_E5 | >1000 | - | 500 | 1000 | >1000 | - | Complex mixture including **3** and **21** |
| 33226_E6 | >1000 | - | 1000 | >1000 | >1000 | - | Complex mixture |
| *Coleus paniculatus* |  |  |  |  |  |  |  |
| 33227_Hex | >1000 | - | 7.8 | 250 | >1000 | - |  |
| 33227_EtOAc | >1000 | - | >1.95 | 7.8 | >1000 | - |  |
| 33227_E1 | >1000 | - | 500 | 500 | >1000 | - | **13** |
| 33227_E2 | >1000 | - | 1.95 | 1.95 | >1000 | - | **1**, **13**, **14** |
| 33227_E3 | >1000 | - | 15.6 | 15.6 | >1000 | - | Fatty acid |
| 33227_E4 | >1000 | - | 1.95 | 1.95 | >1000 | - | **2** |
| 33227_E5 | >1000 | - | 125 | 125 | >1000 | - | Complex mixture |
| 33227_E6 | >1000 | - | 7.8 | 62.5 | >1000 | - | Derivatives of **1** and **2** |
| 33227_E7 | >1000 | - | 125 | 500 | >1000 | - | Complex mixture |
| *Coleus sp.* |  |  |  |  |  |  |  |
| 1829_Hex | >1000 | - | 125 | 1000 | >1000 | - |  |
| 1829_EtOAc | >1000 | - | 125 | 1000 | >1000 | - |  |
| 1829_E1 | >1000 | - | 125 | 1000 | >1000 | - | **16**-**18** |
| 1829_E2 | >1000 | - | 125 | 1000 | >1000 | - | **16**-**18** |
| 1829_E3 | >1000 | - | 15.6 | 62.5 | >1000 | - | **8** |
| 1829_E4 | >1000 | - | 62.5 | 250 | >1000 | - | Complex mixture containing **8** |
| 1829_E5 | >1000 | - | 15.6 | 62.5 | >1000 | - | Complex mixture containing **8** |
| *Coleus crassus* |  |  |  |  |  |  |  |
| 1671_Hex | >1000 | - | 7.8 | 15.6 | >1000 | - |  |
| 1671_EtOAc | >1000 | - | <1.95 | 15.6 | >1000 | - |  |
| 1671_E1 |  |  |  |  |  |  | Fatty acid |
| 1671_E2 | >1000 | - | <1.95 | 3.9 | >1000 | - | **1**, **14**, coleon V-quinone |
| 1671_E3 | >1000 | - | <1.95 | 31.3 | >1000 | - | **2** |
| 1671_E4 | >1000 | - | <1.95 | 7.8 | >1000 | - | **2** |
| 1671_E5 | >1000 | - | <1.95 | 7.8 | >1000 | - | **1**, **2** |
| 1671_E6 | >1000 | - | <1.95 | 7.8 | >1000 | - | **1**, **2**, Coleon U-quinone |
| 1671_E7/8 | >1000 | - | 7.8 | 62.5 | >1000 | - | Complex mixture |
| 1671_E9 | >1000 | - | 15.6 | 15.6 | >1000 | - | Complex mixture |

Positive control: Zinc pyrithione (MIC/MBC 0.98 *µ*g/mL / 125 *µ*g/mL) for antifungal activity and piroctone olamine (MIC/MBC 15.6 *µ*g/mL / 250 *µ*g/mL) for antibacterial activity.

Samples are labelled with BI number of each plant as stated in Table 1, followed by the extract type (Hex for hexane and EtOAc for ethyl acetate extracts) and fraction numbers as obtained from the flash chromatography fractionation of the EtOAc extracts


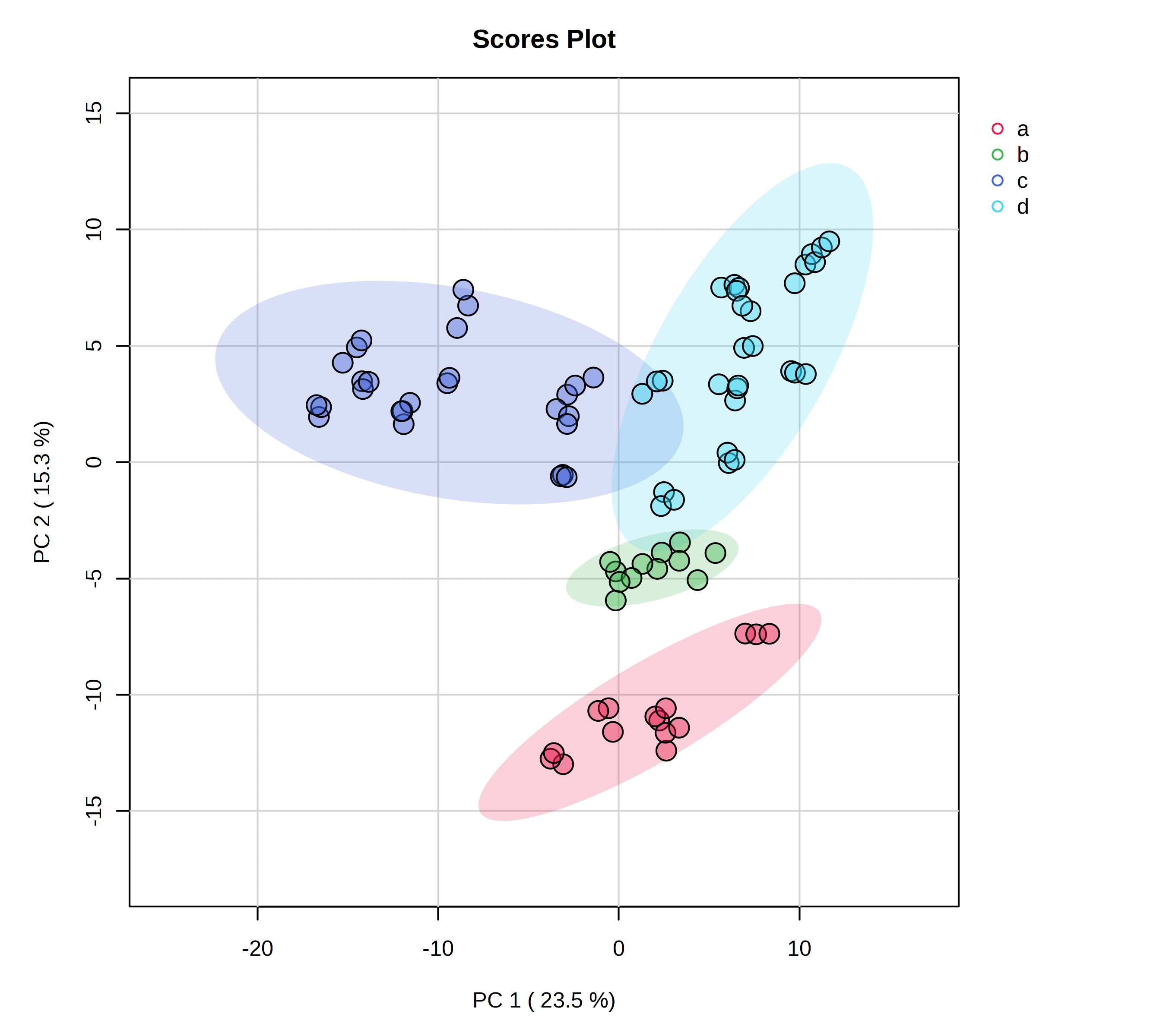


Figure S1. Principal component analysis (PCA) of the 28 plants studied generated from the most significant features of each plant retrieved from both positive and negative modes LCMS of the plant EtOAc extracts


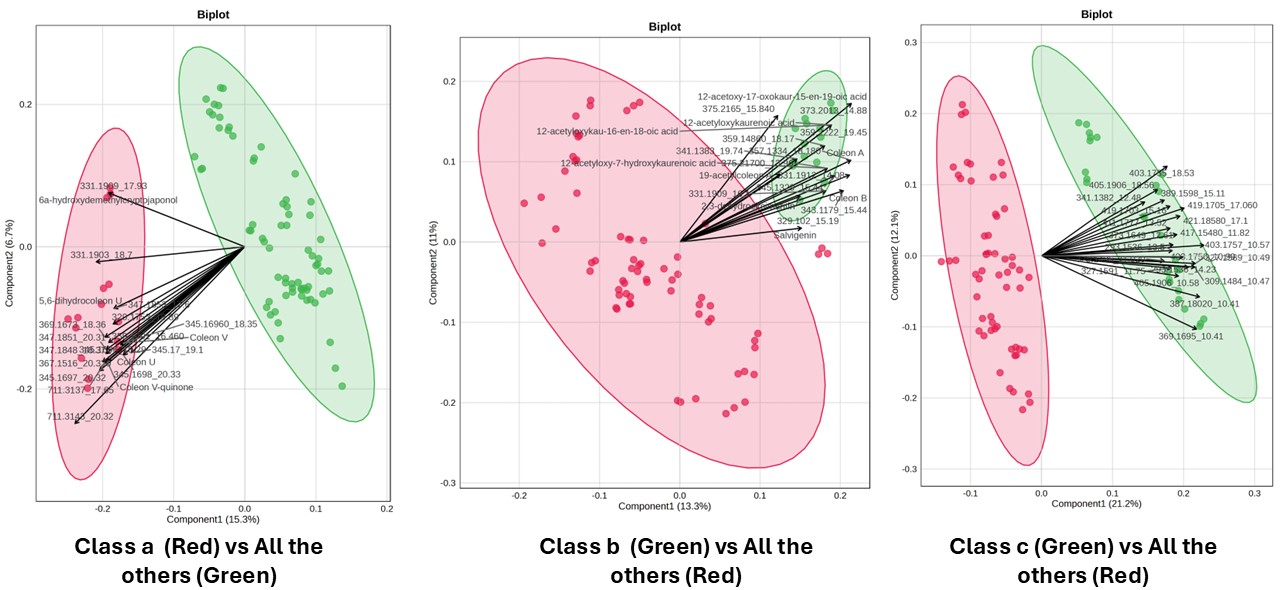


Figure S2. PLSDA biplots of the comparison of each of the classes (or groups) showing the first 21 most important features needed to separate a class (or group) from the others. Important to notice here that Classes are different from the Groups generated from the analysis (class A = Group 1; Class B = Group C; Class C = Group B; Class D = Group D).

Figures S3. ^1^H NMR spectra of fractions in Table S1


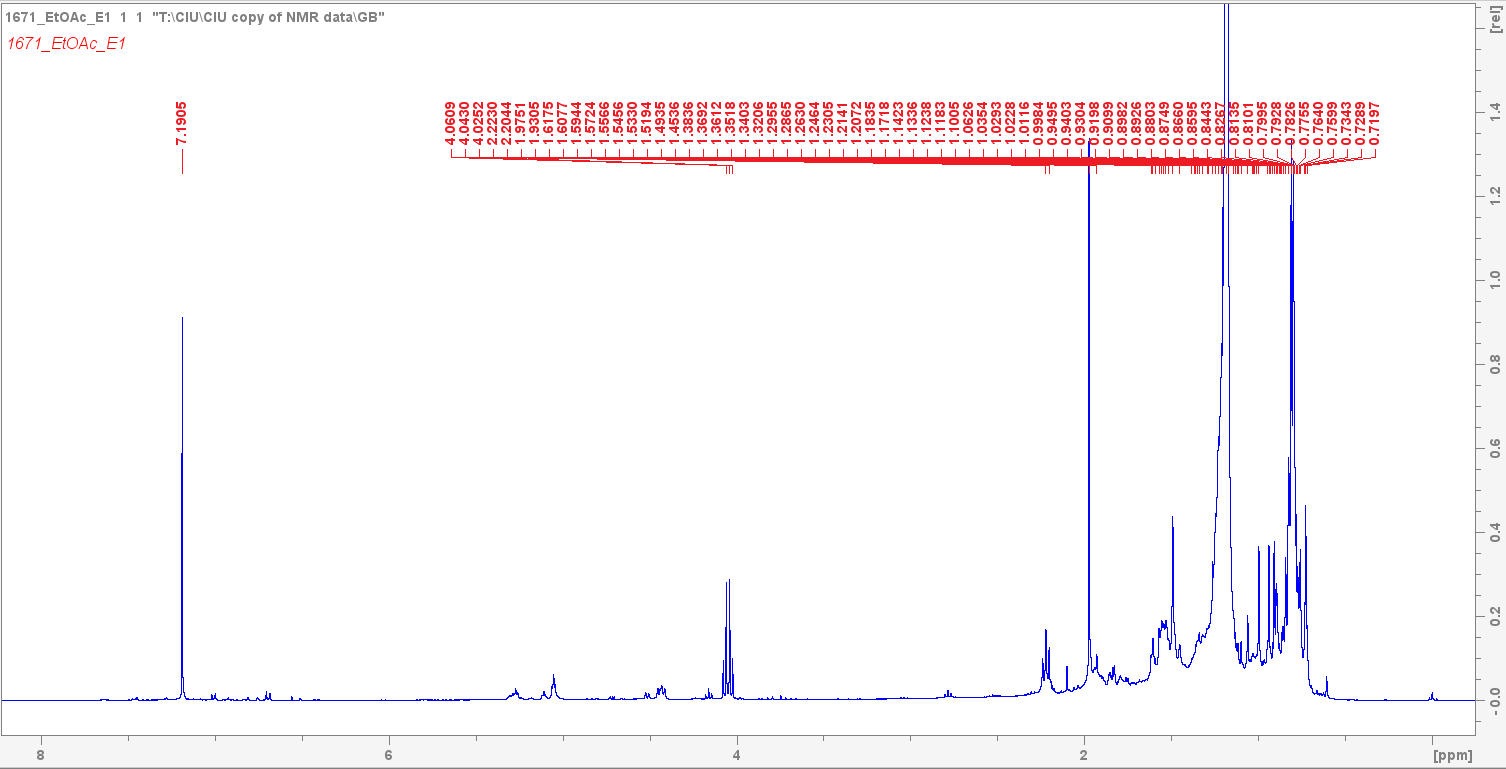


1671_E1


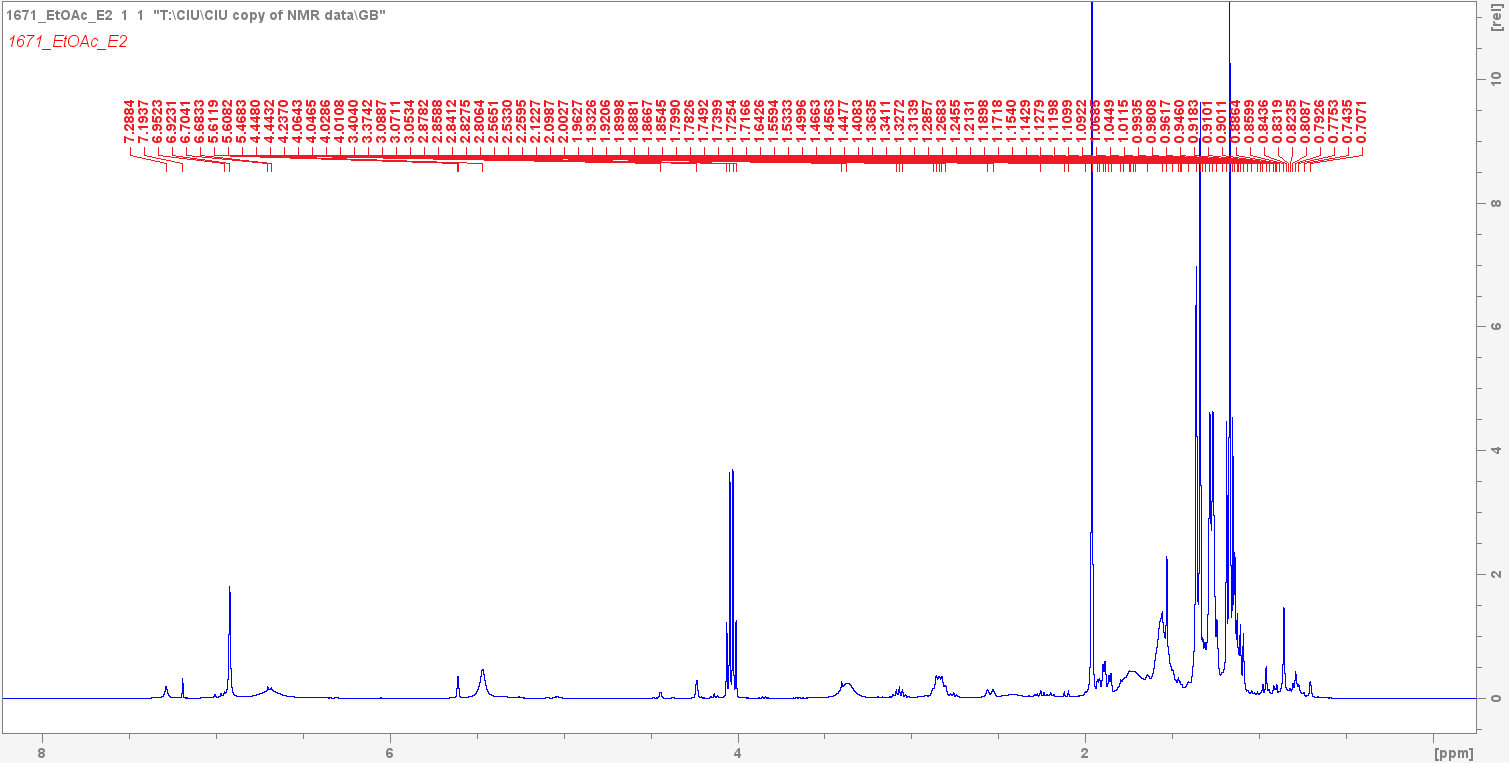


1671_E2


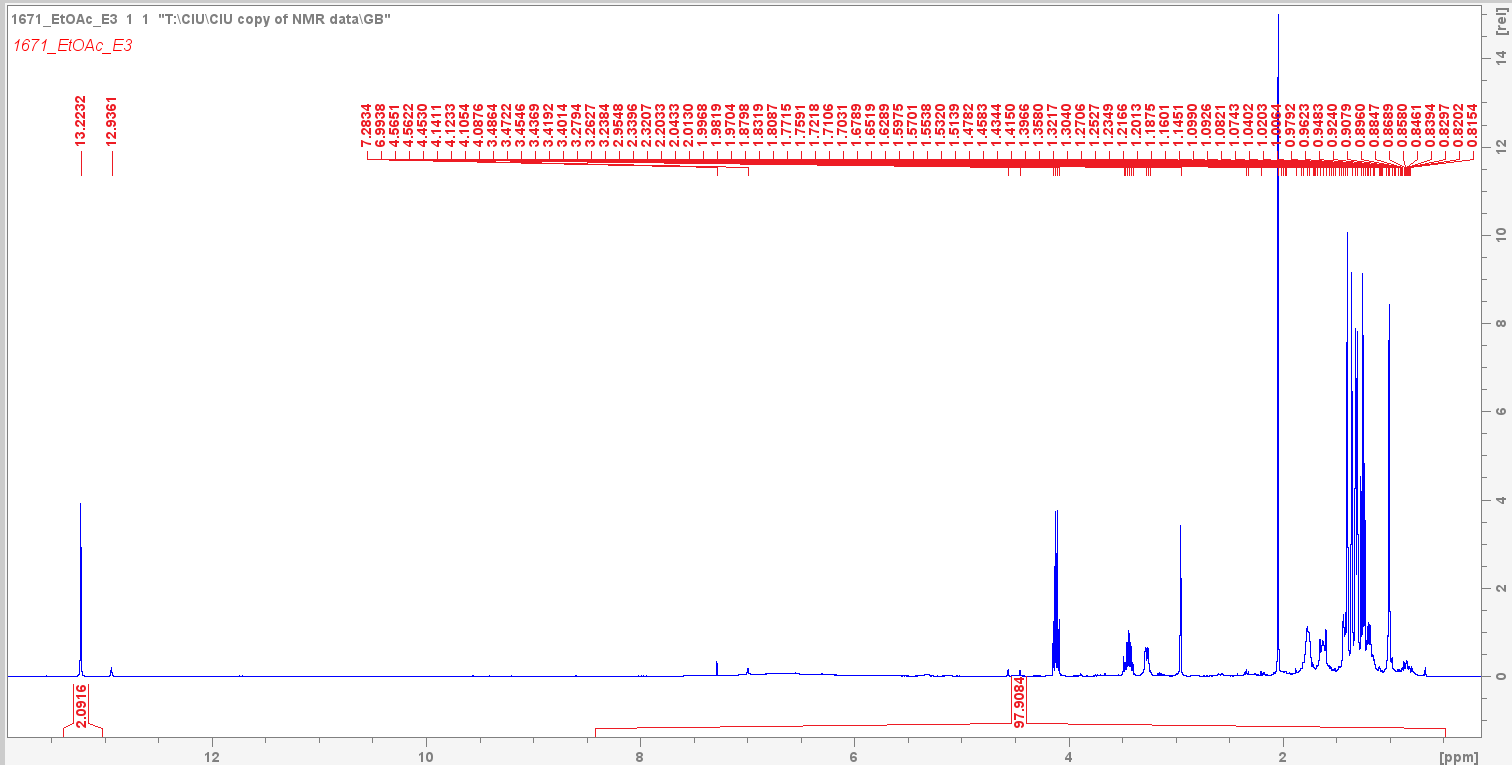


1671_E3


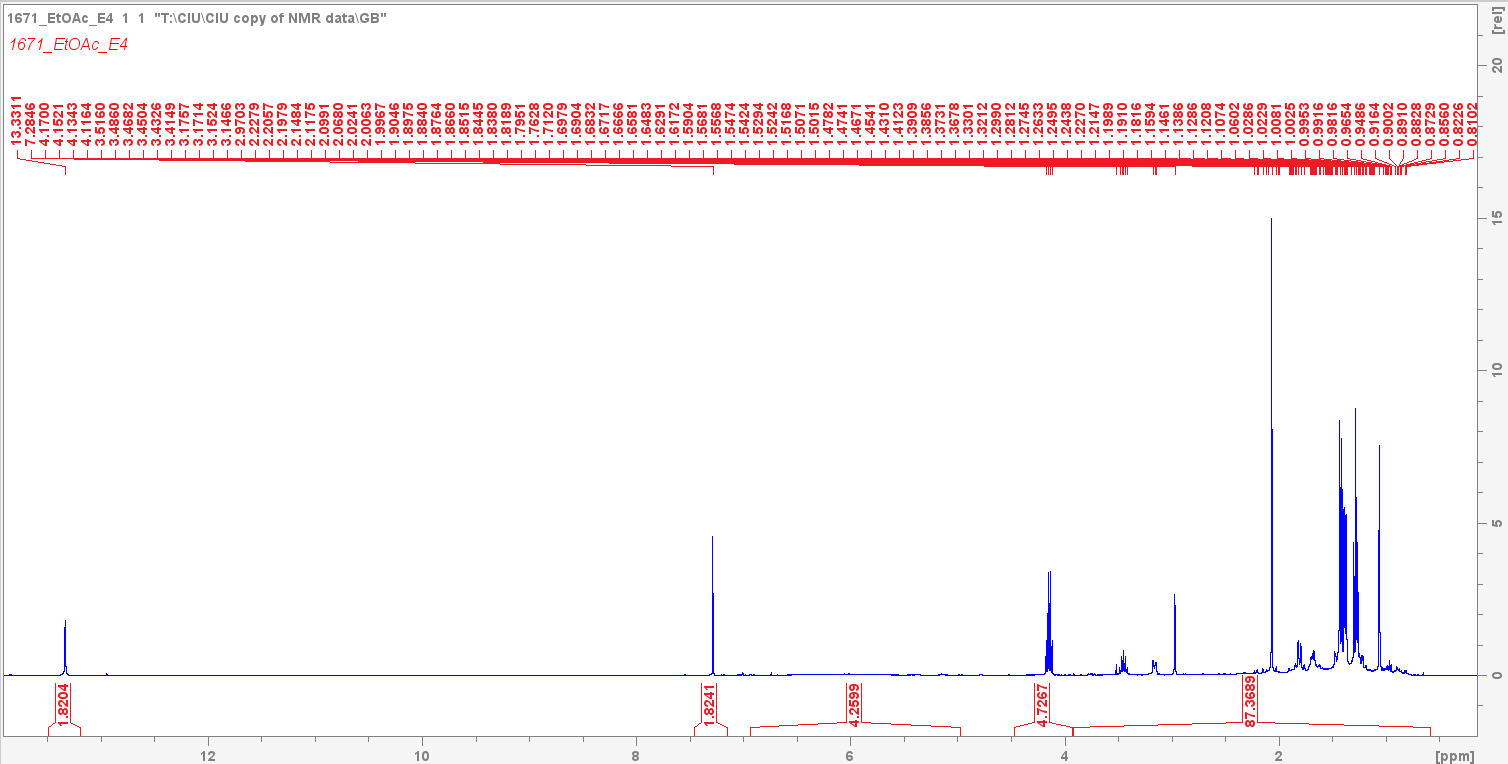


1671_E4


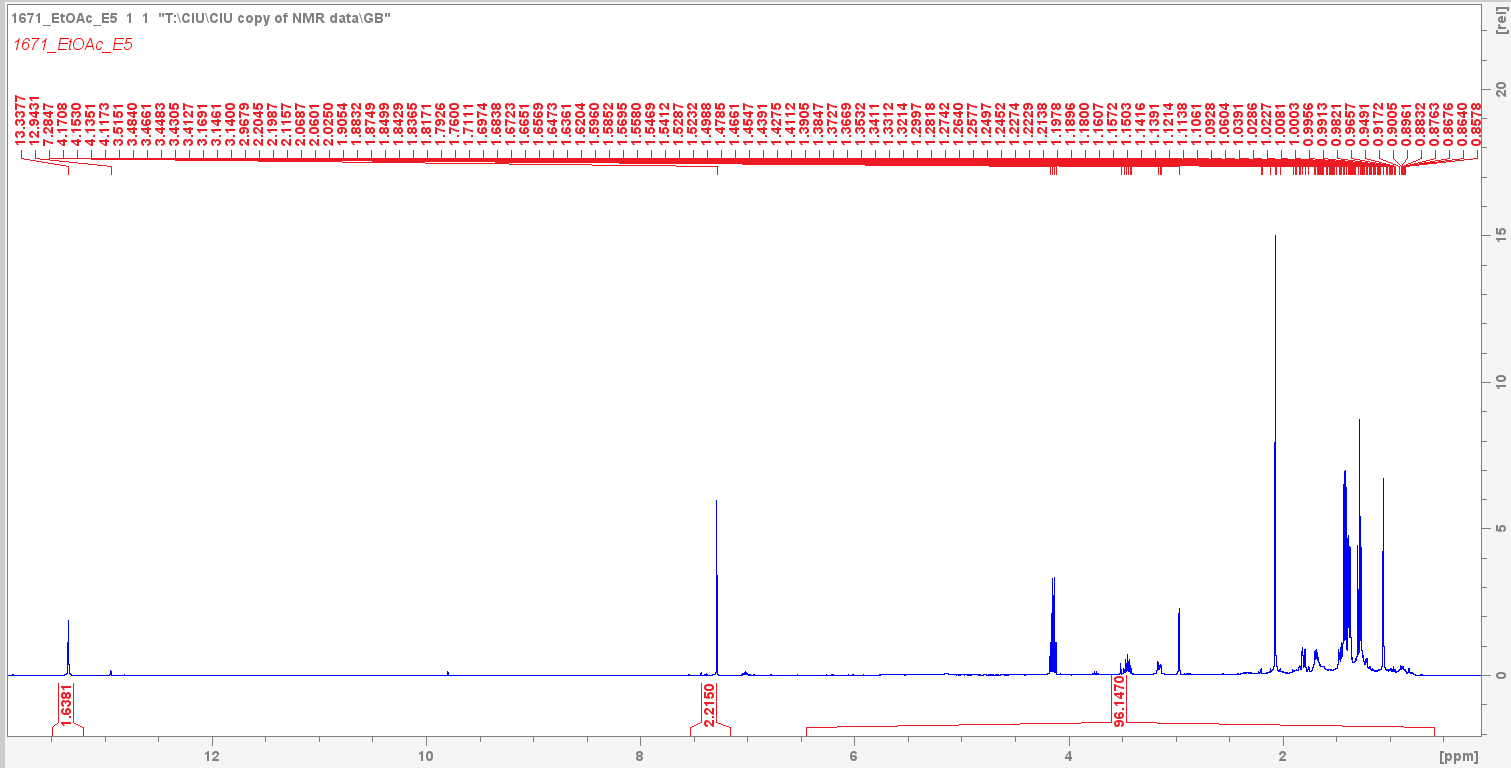


1671_E5


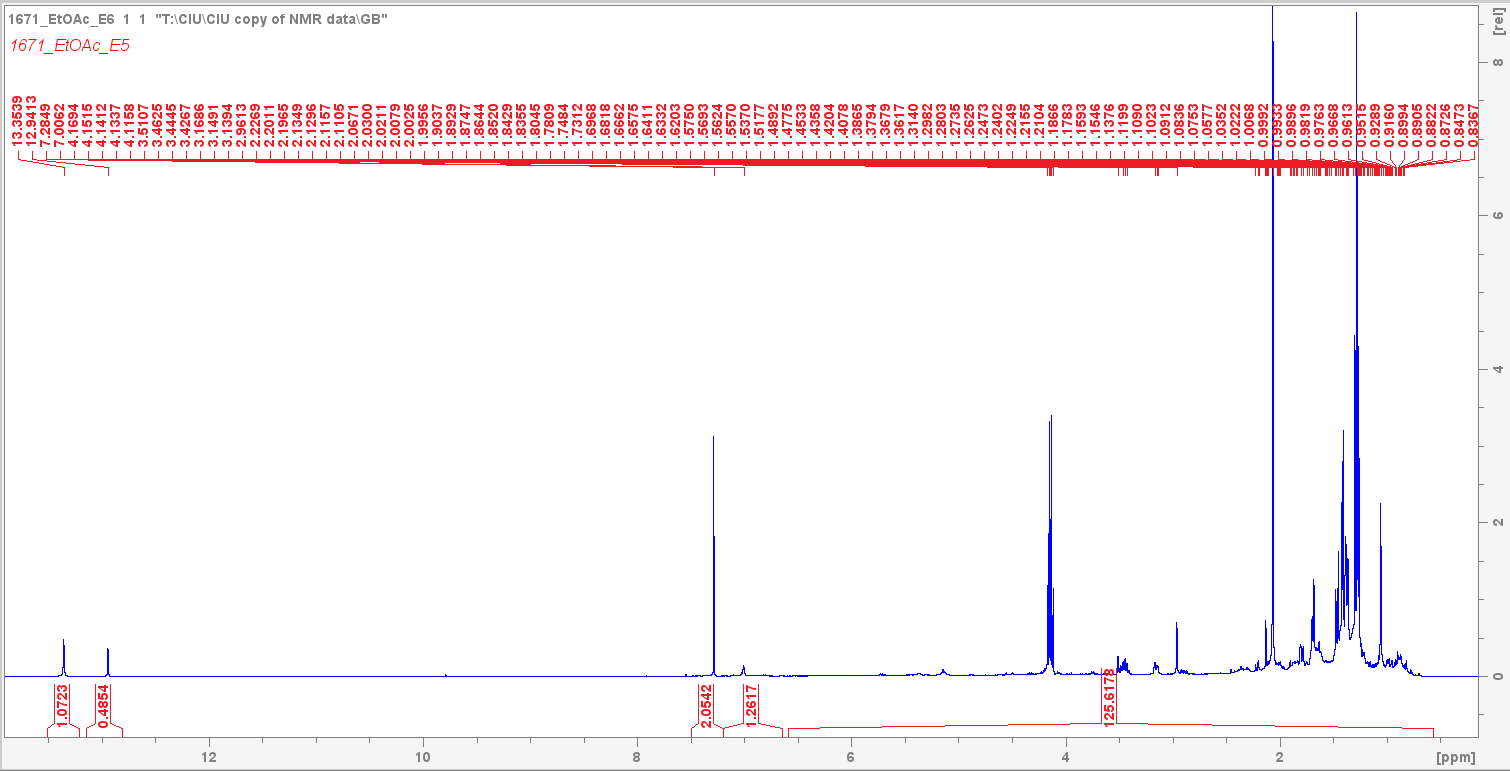


1671_E6


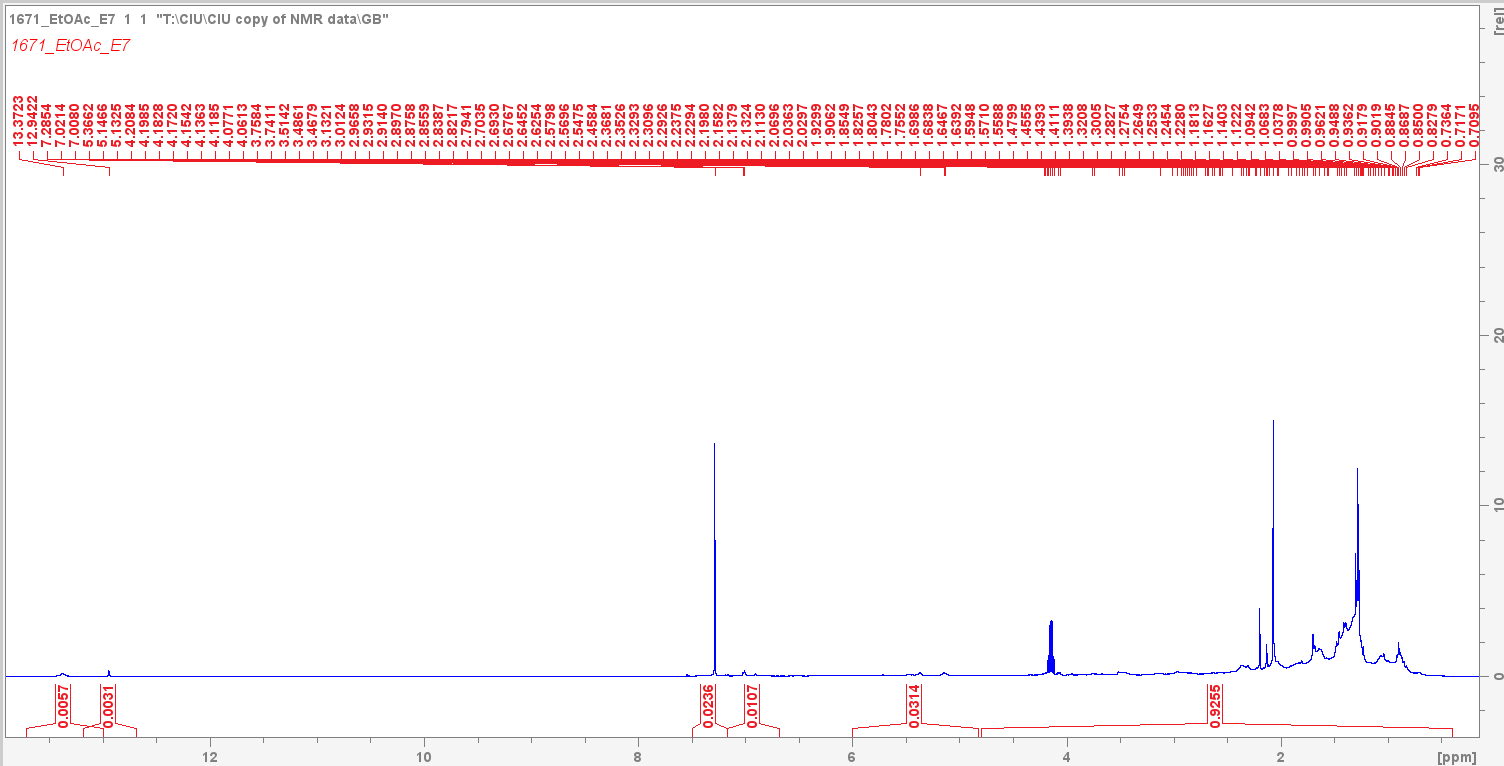


1671_E7


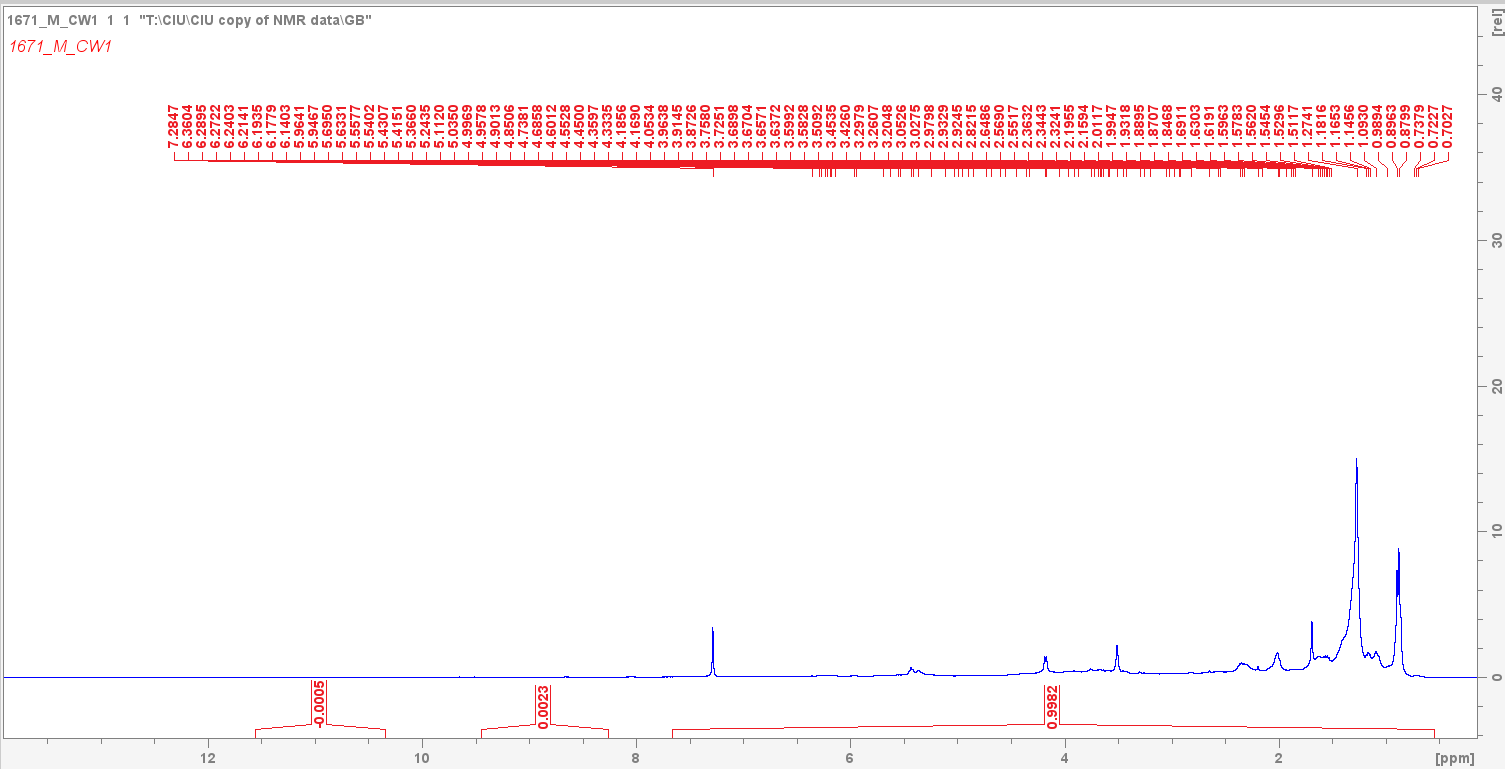


1671_E9


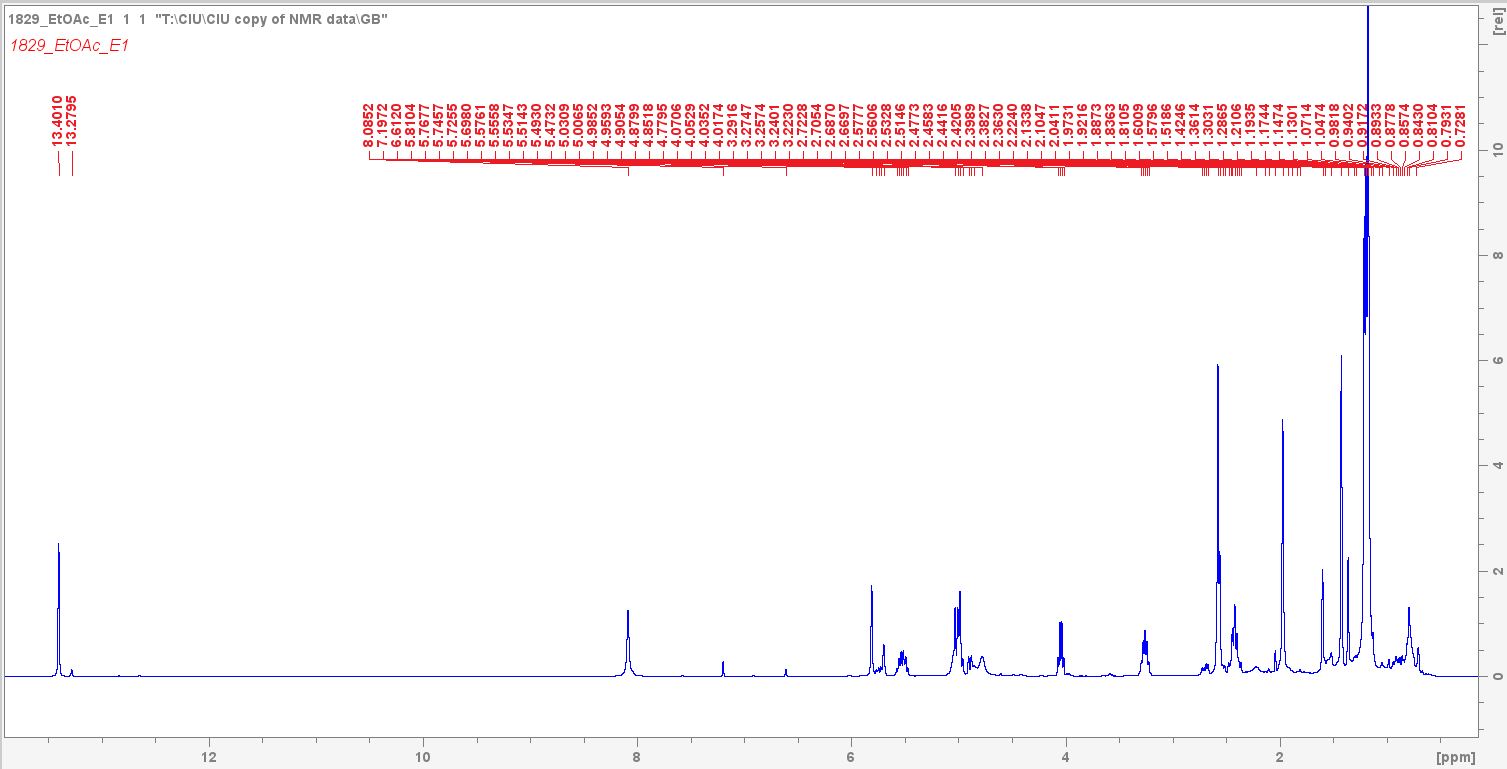


1829_E1


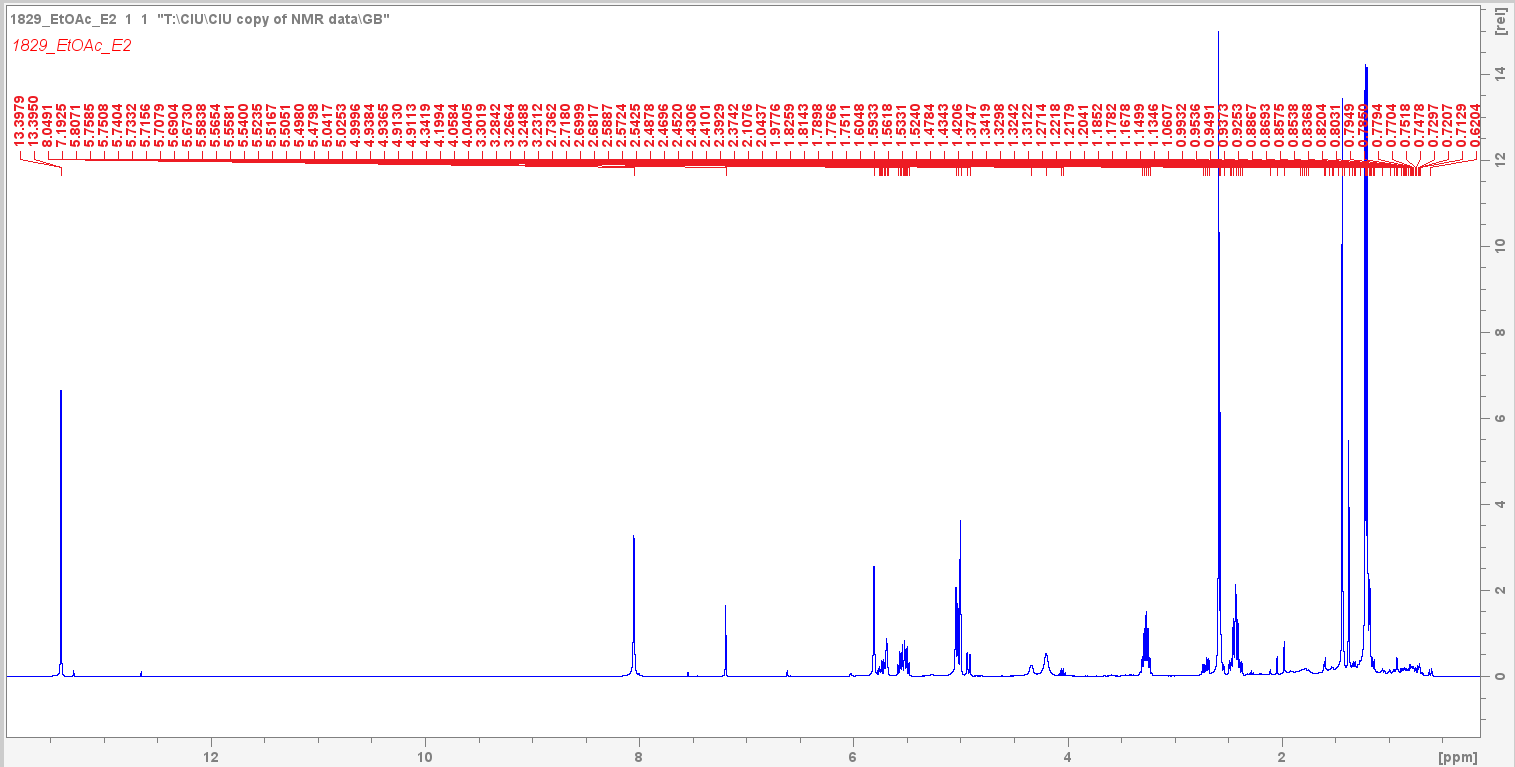


1829_E2


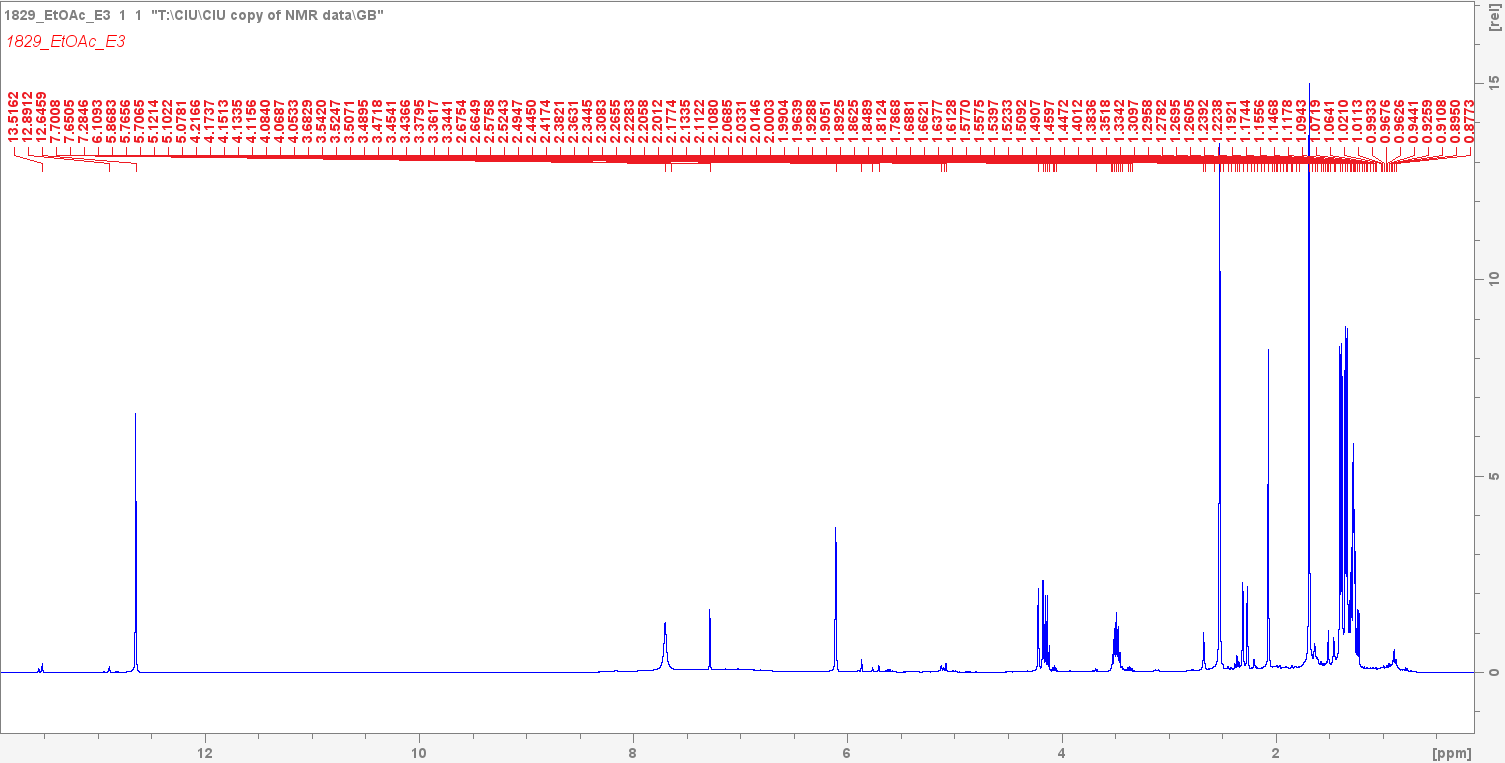


1829_E3


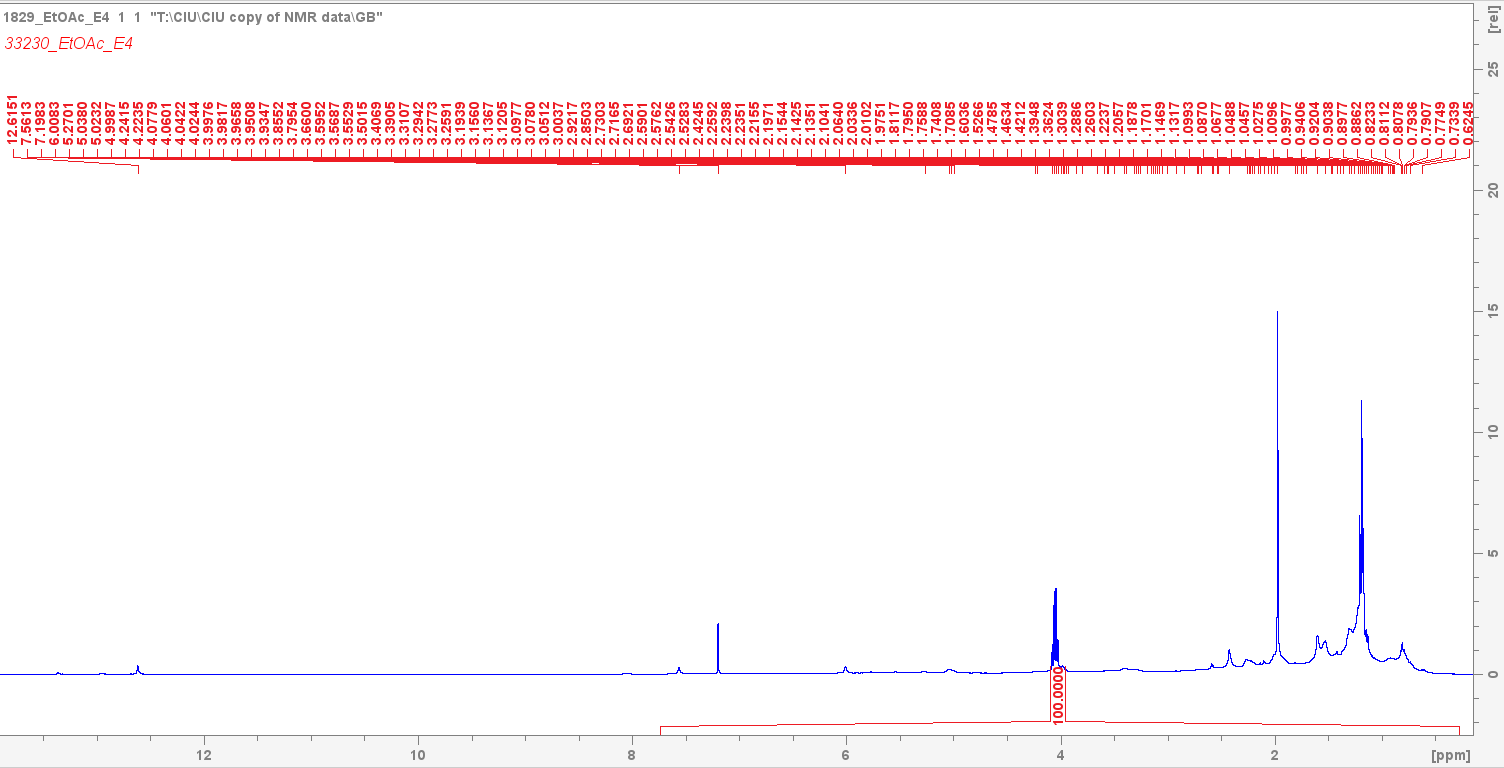


1829_E4


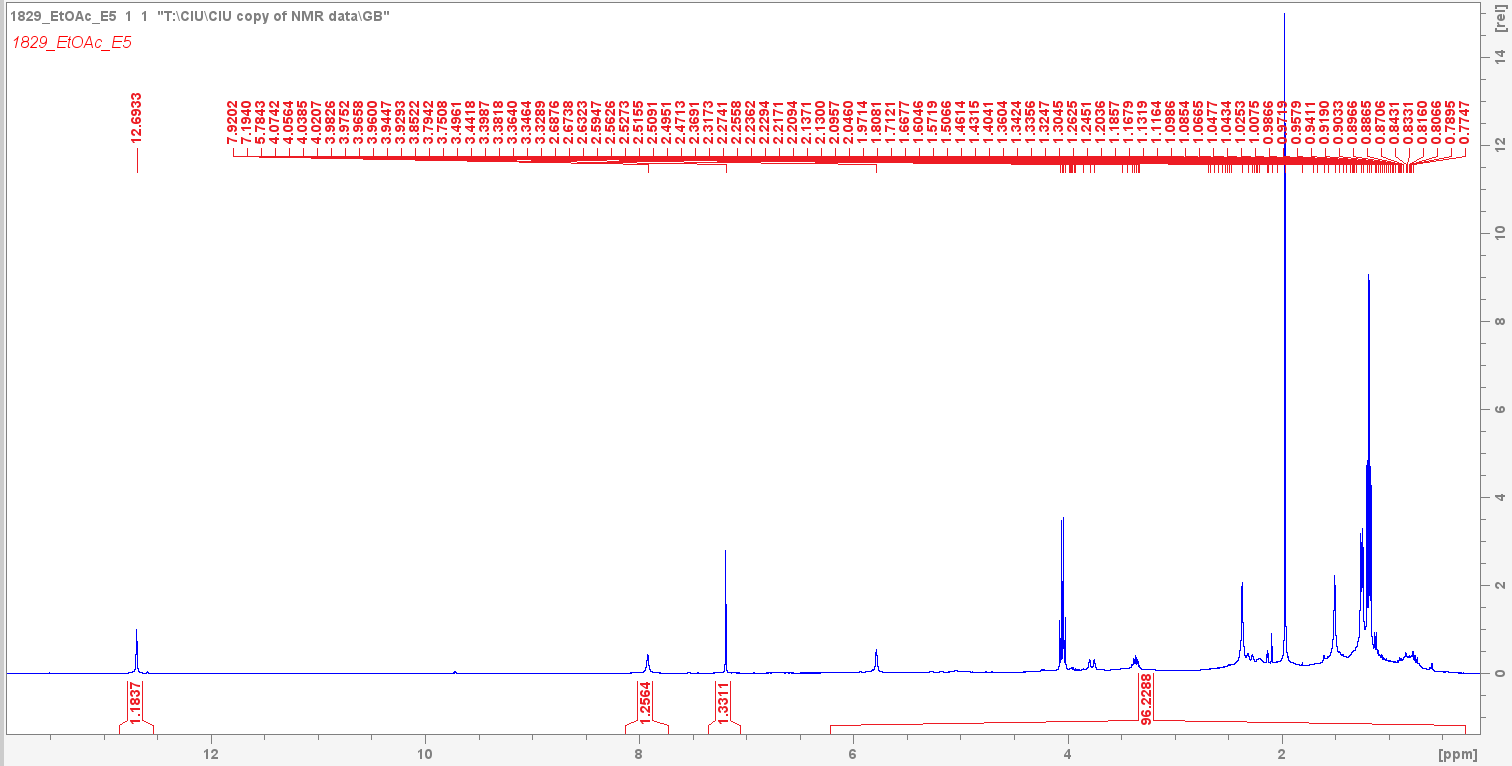


1829_E5


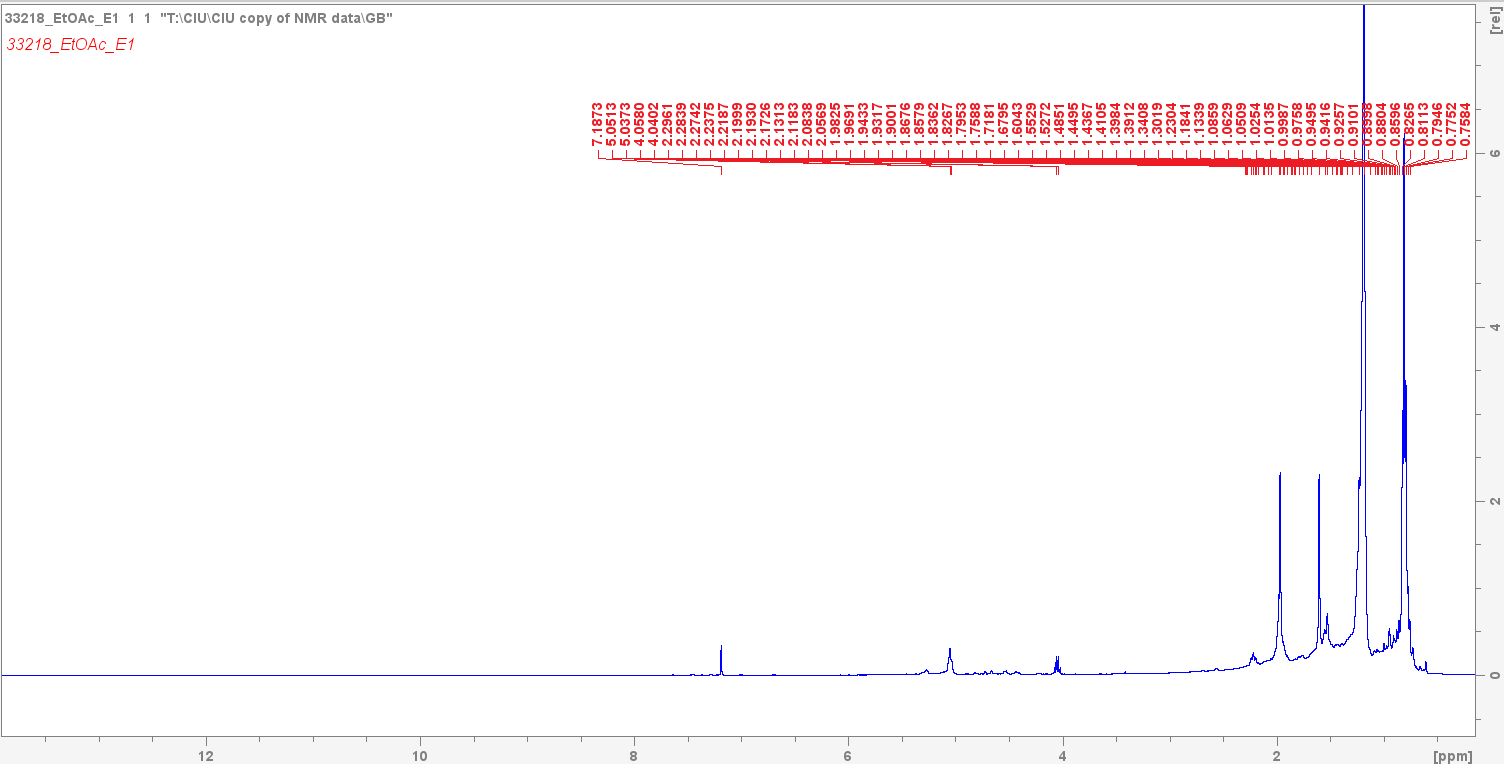


33218_E1


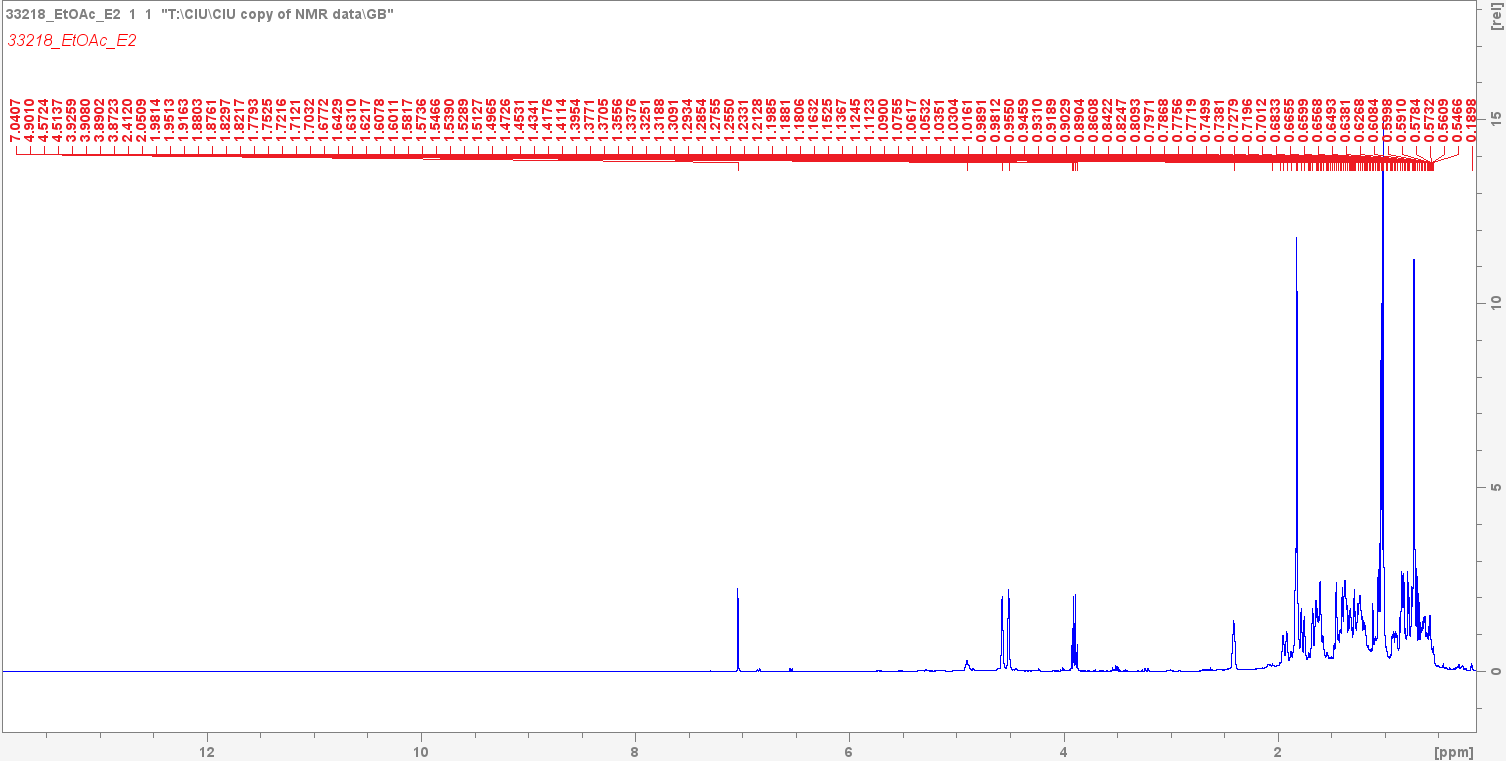


33218_E2


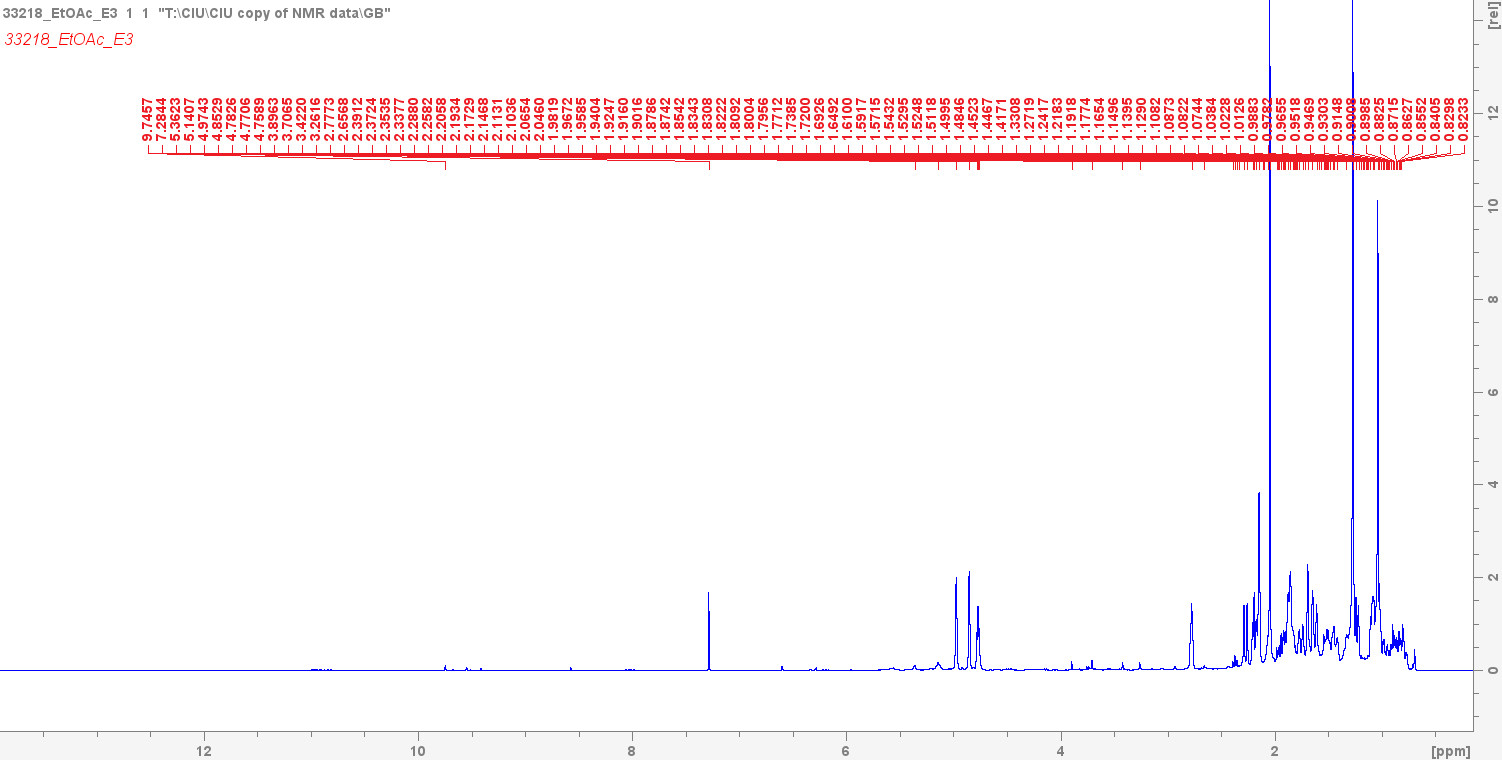

33218_E3


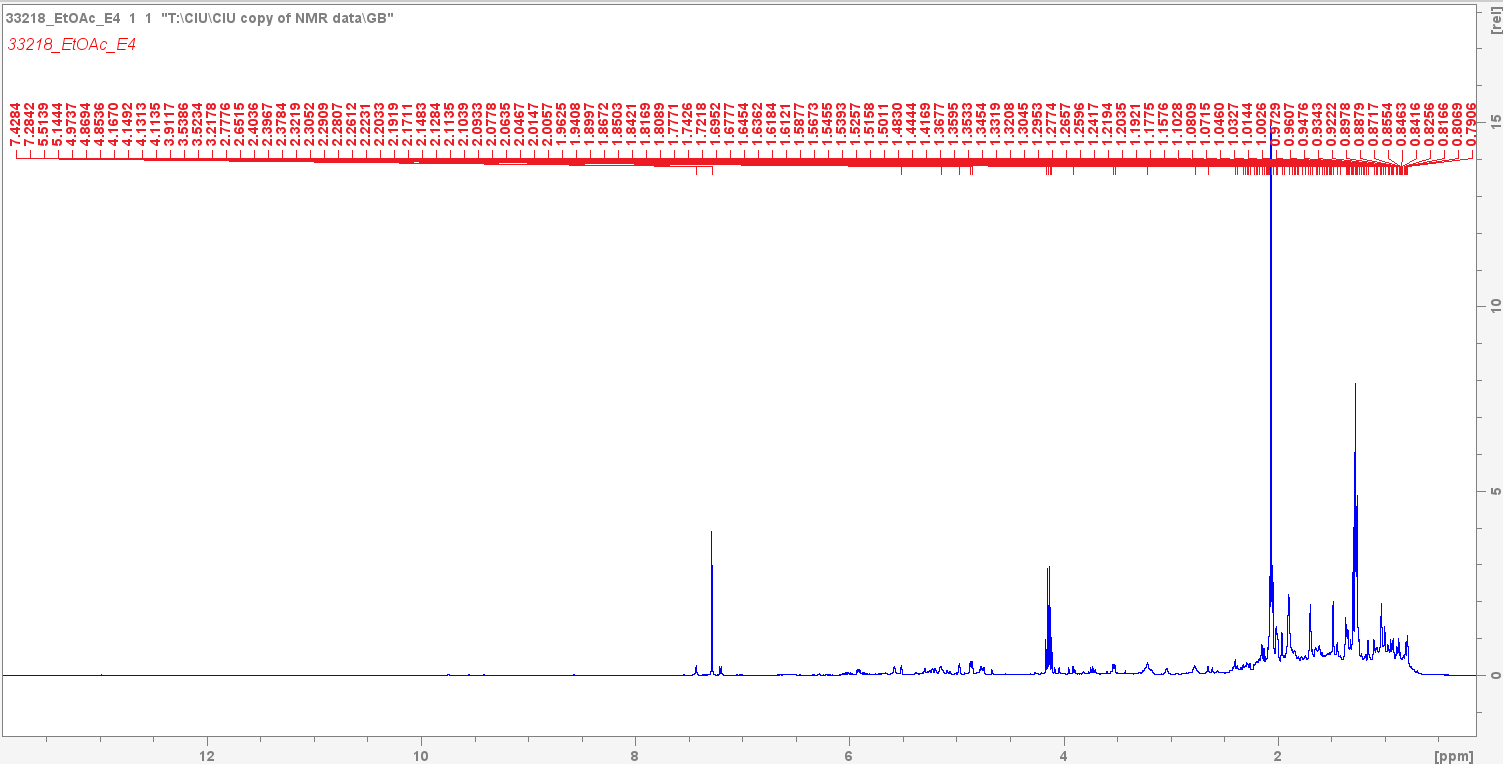


33218_E4


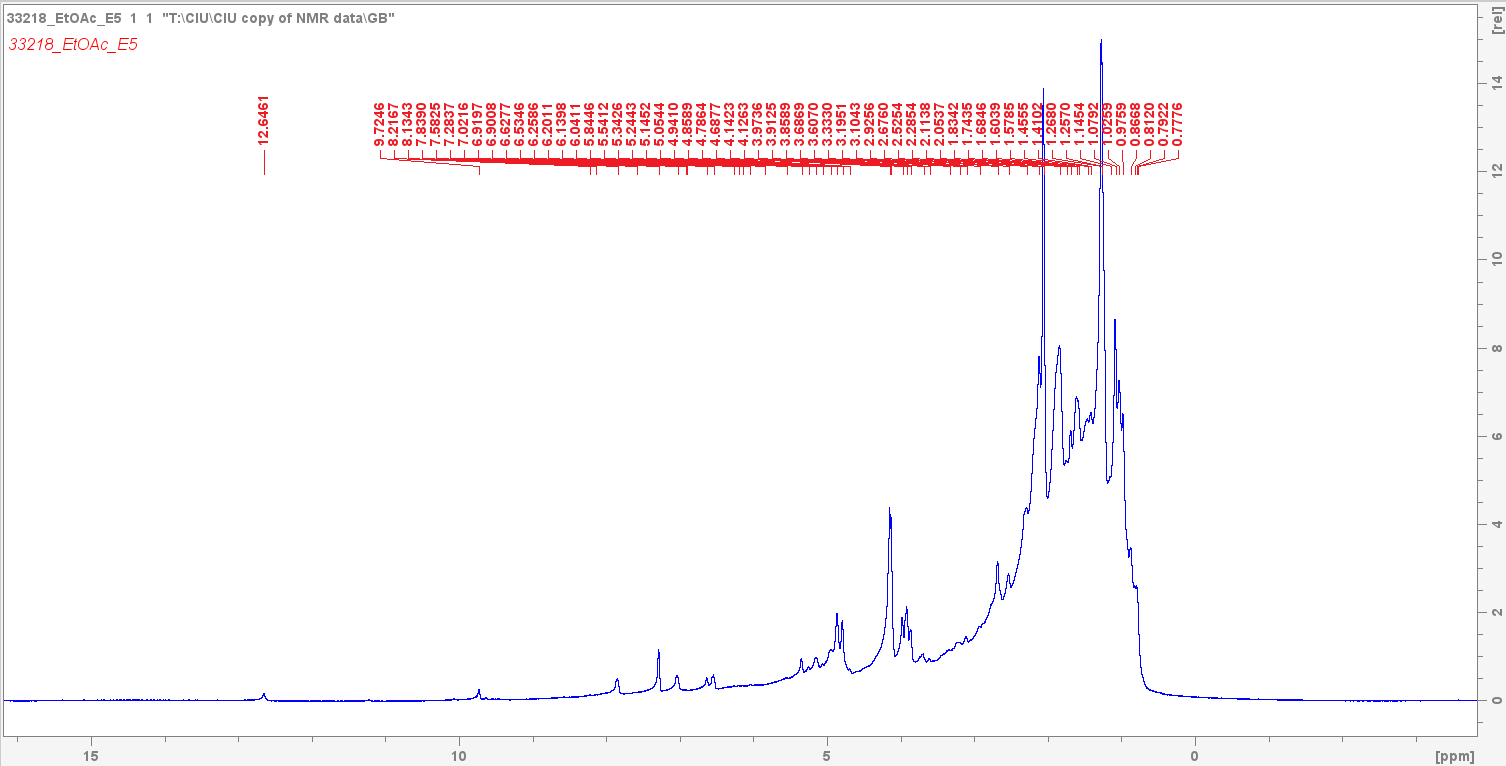


33218_E5


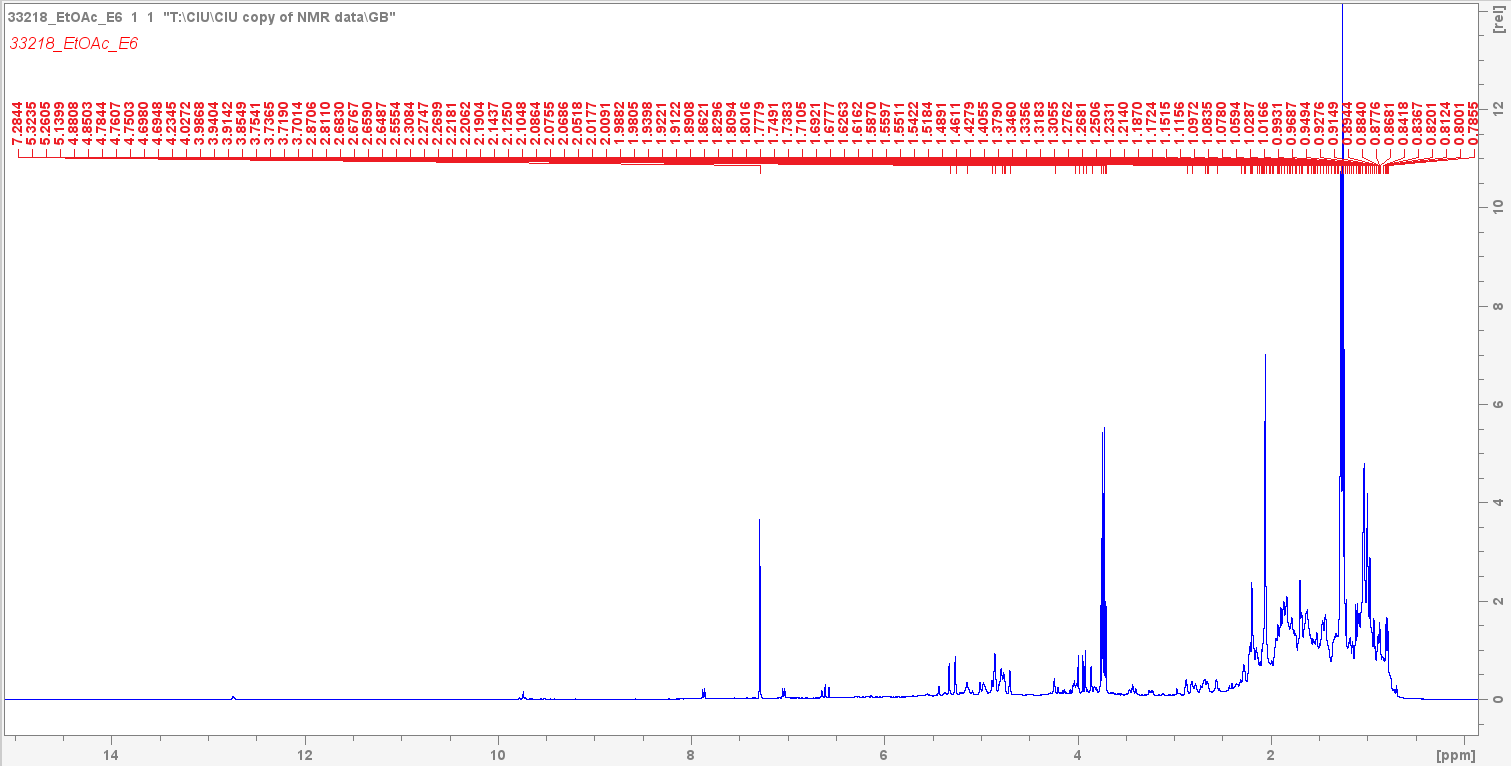


33218_E6


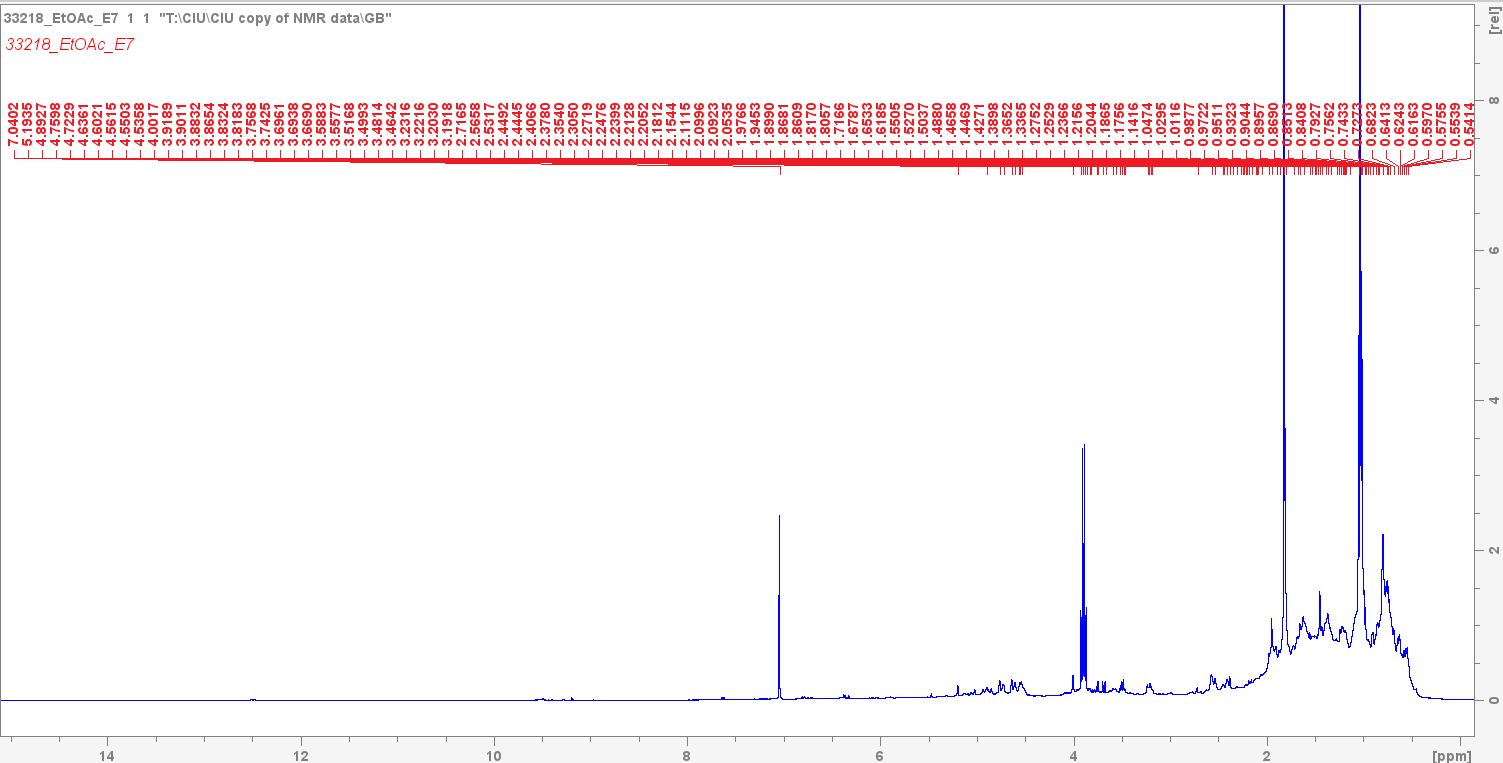


33218_E7


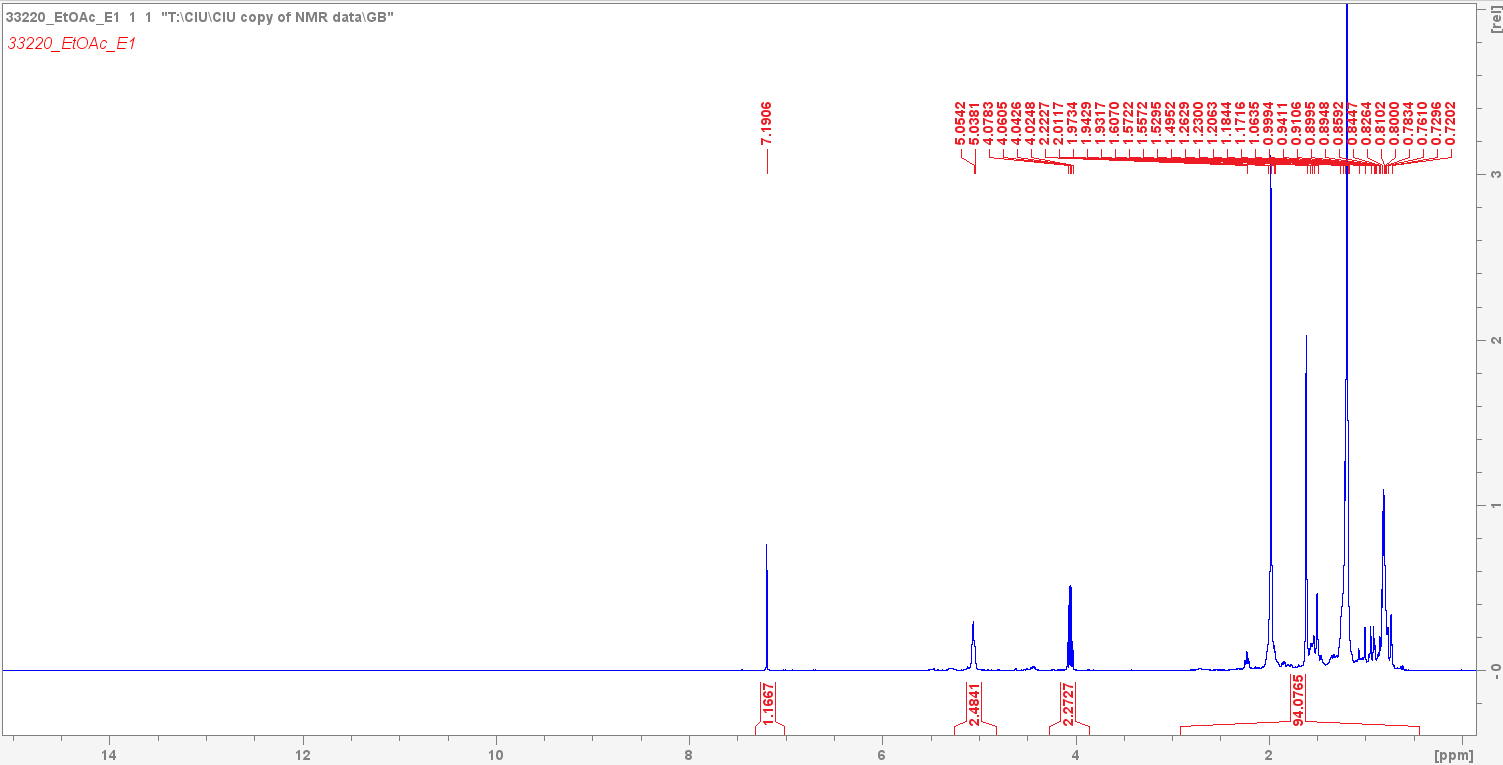


33220_E1


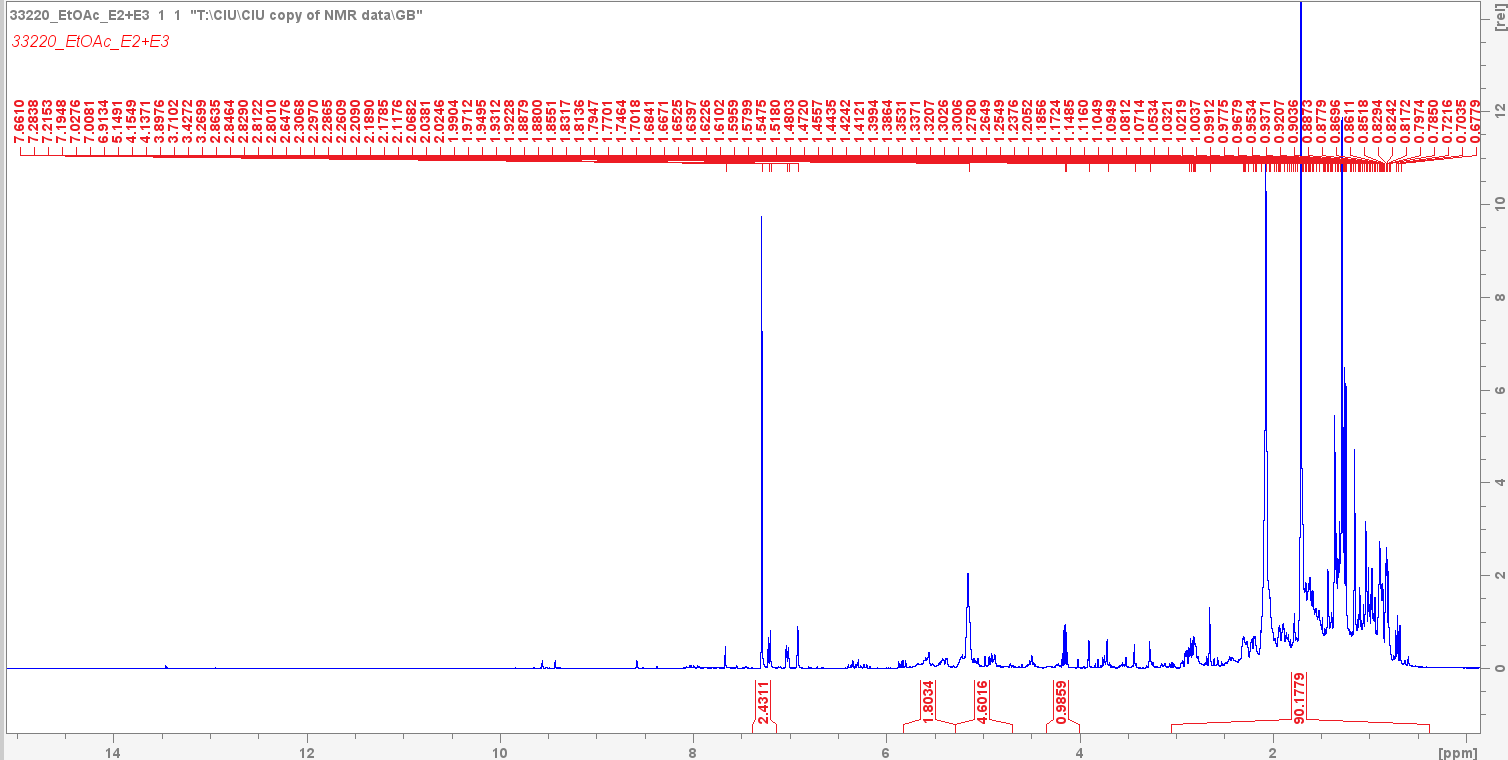


33220_E2


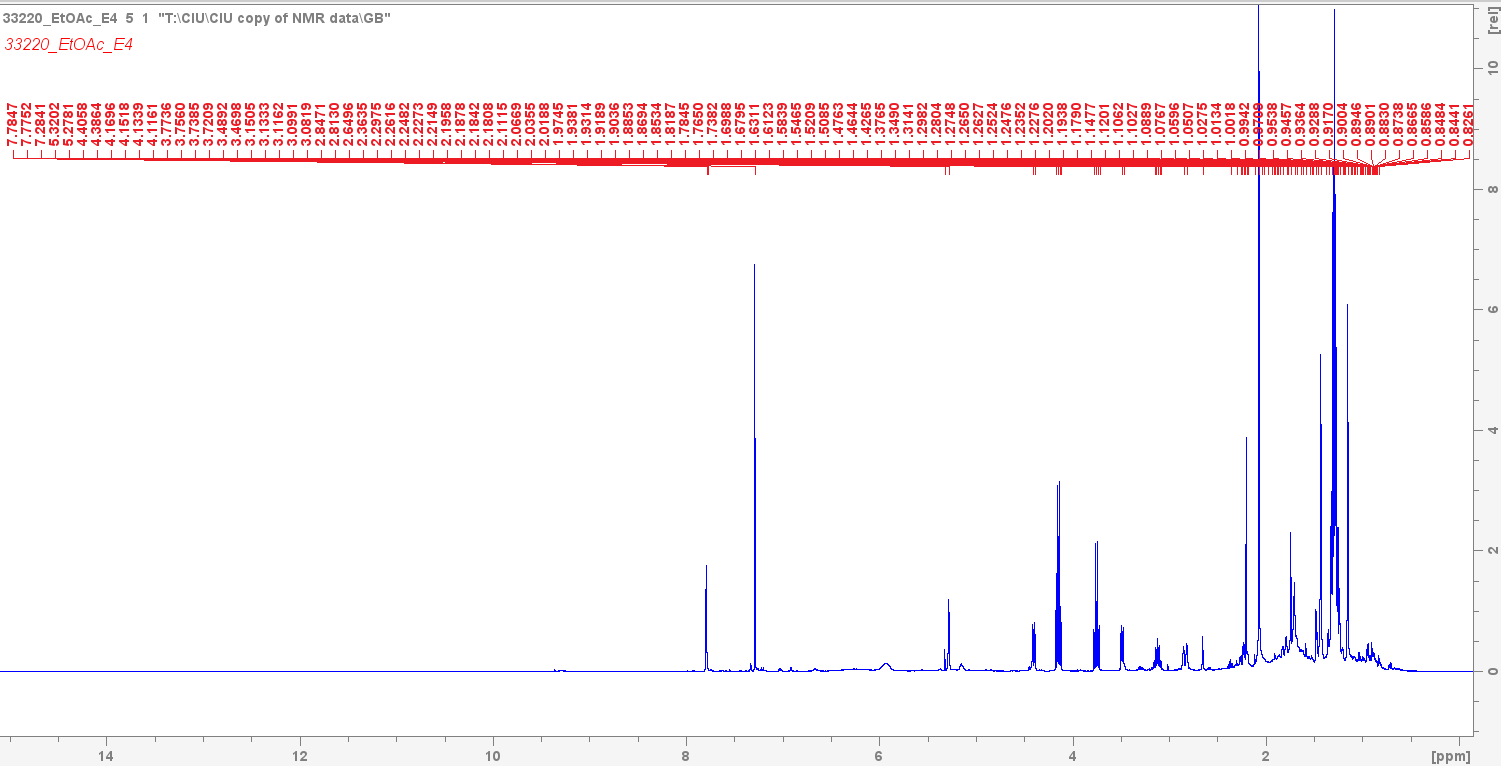


33220_E4


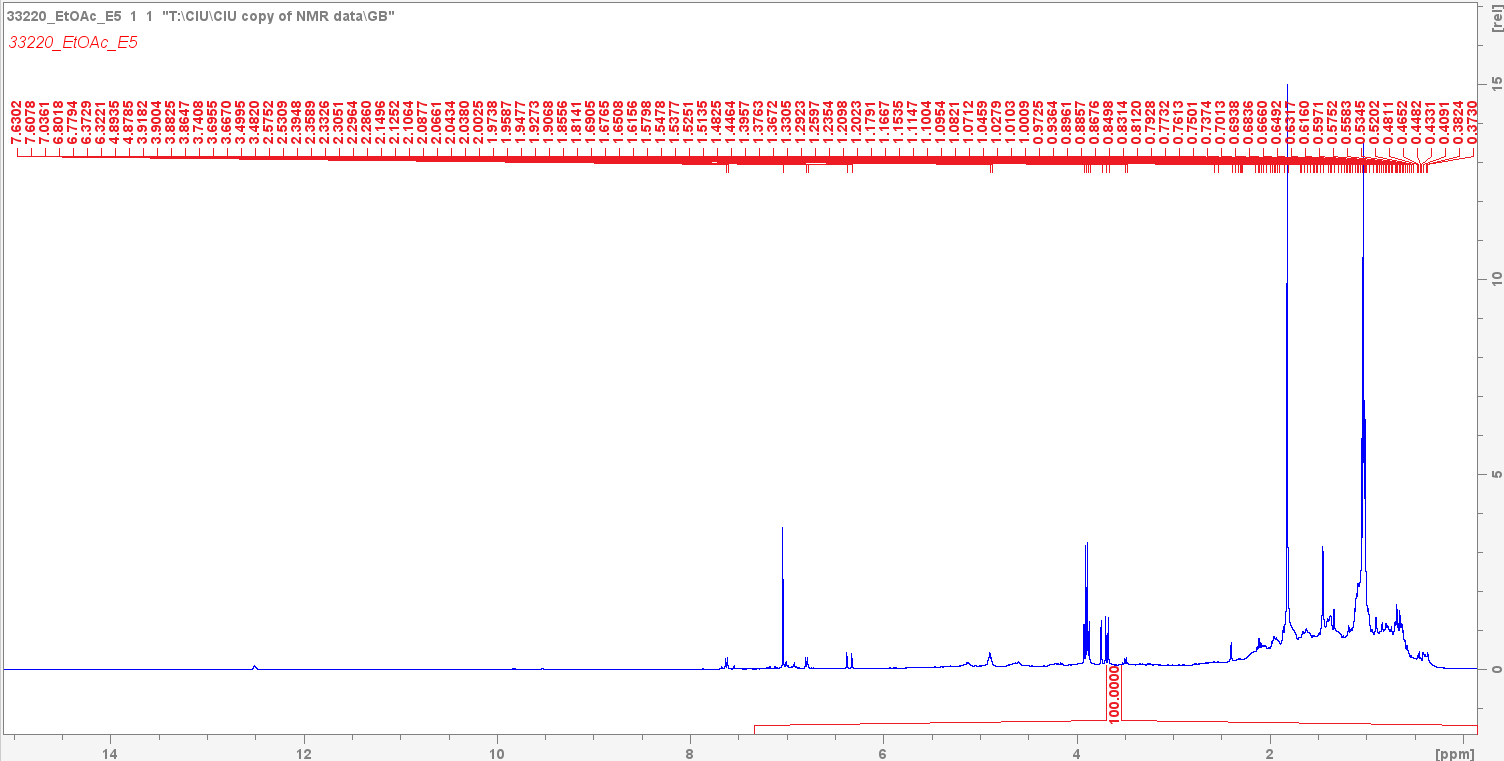


33220_E5


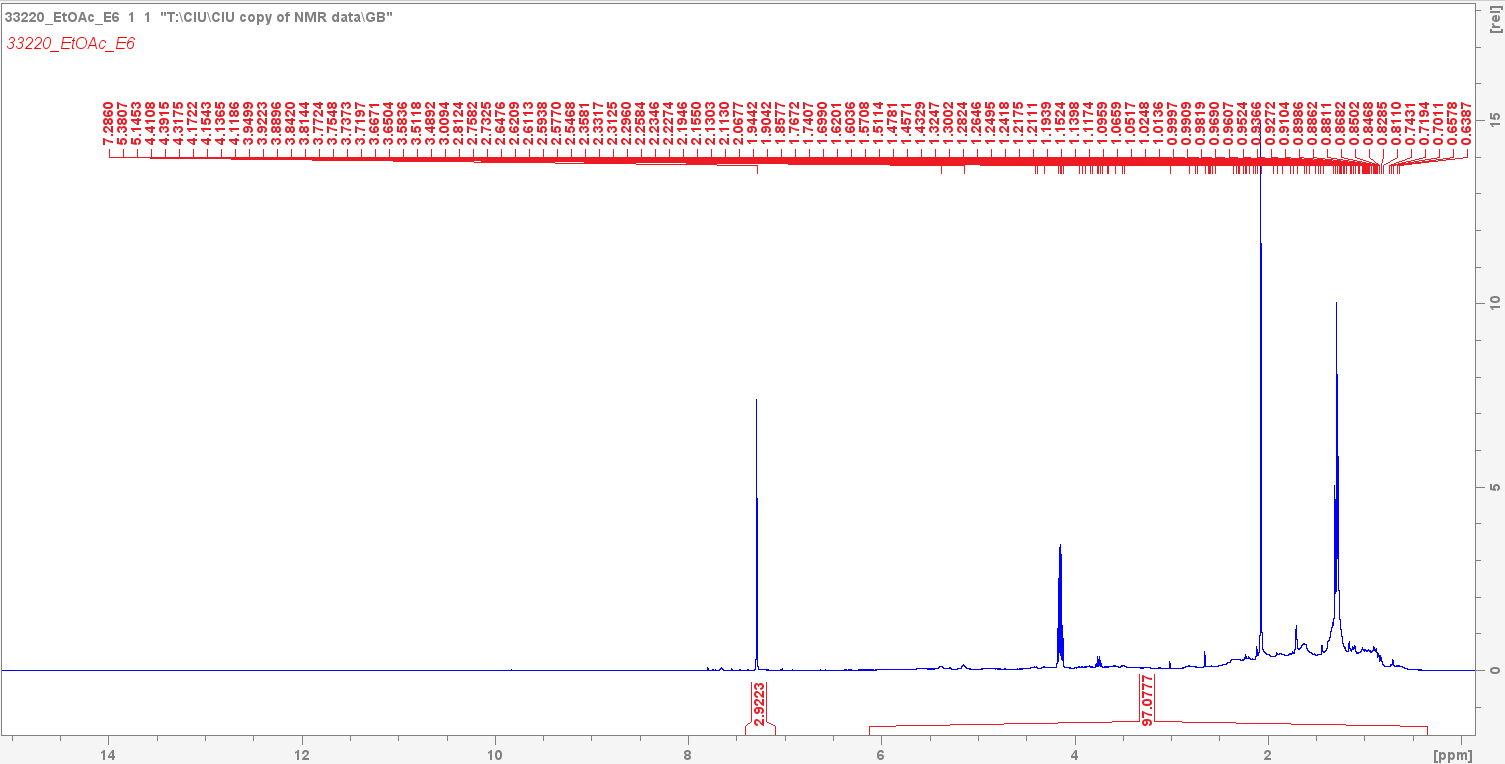


33220_E6


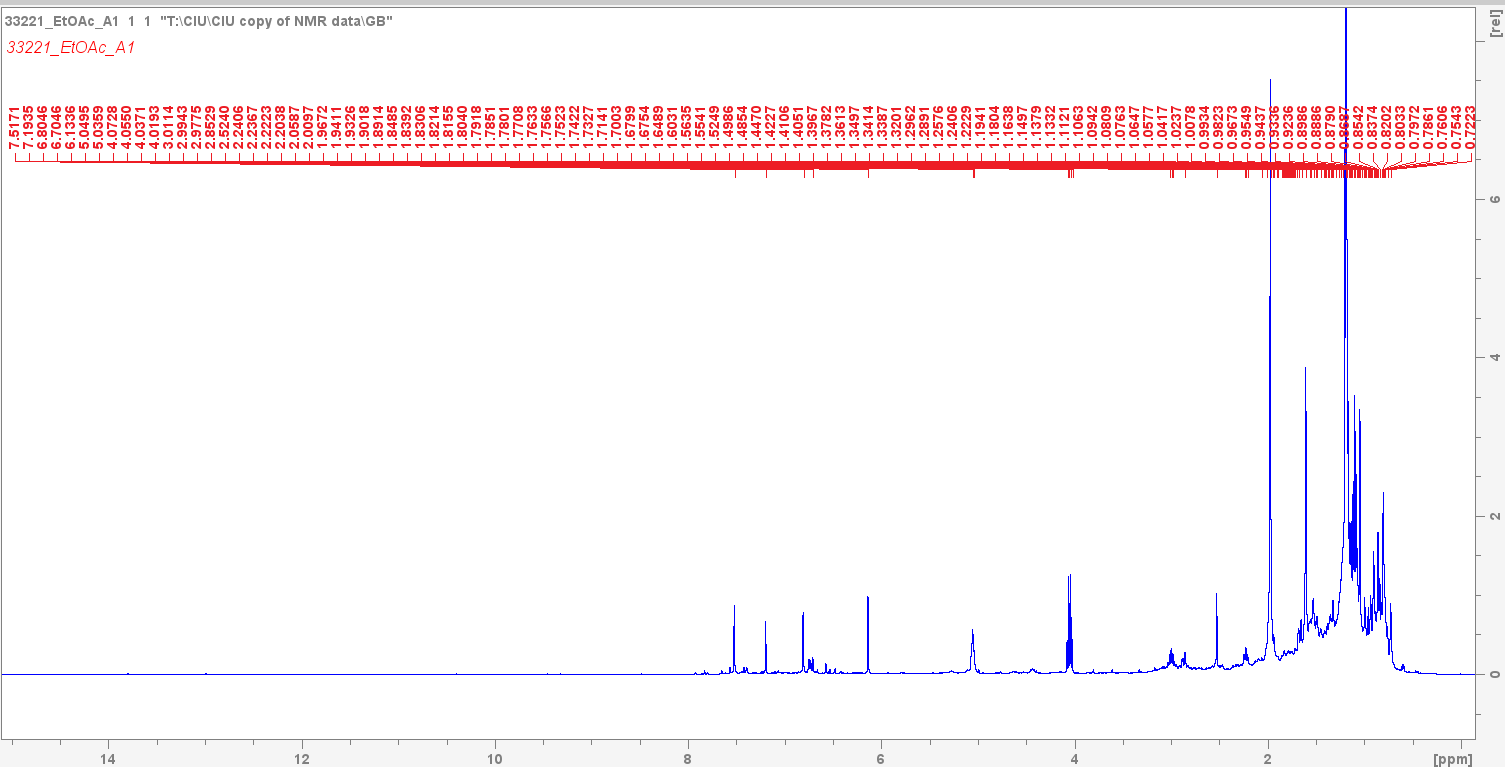


33221_E1


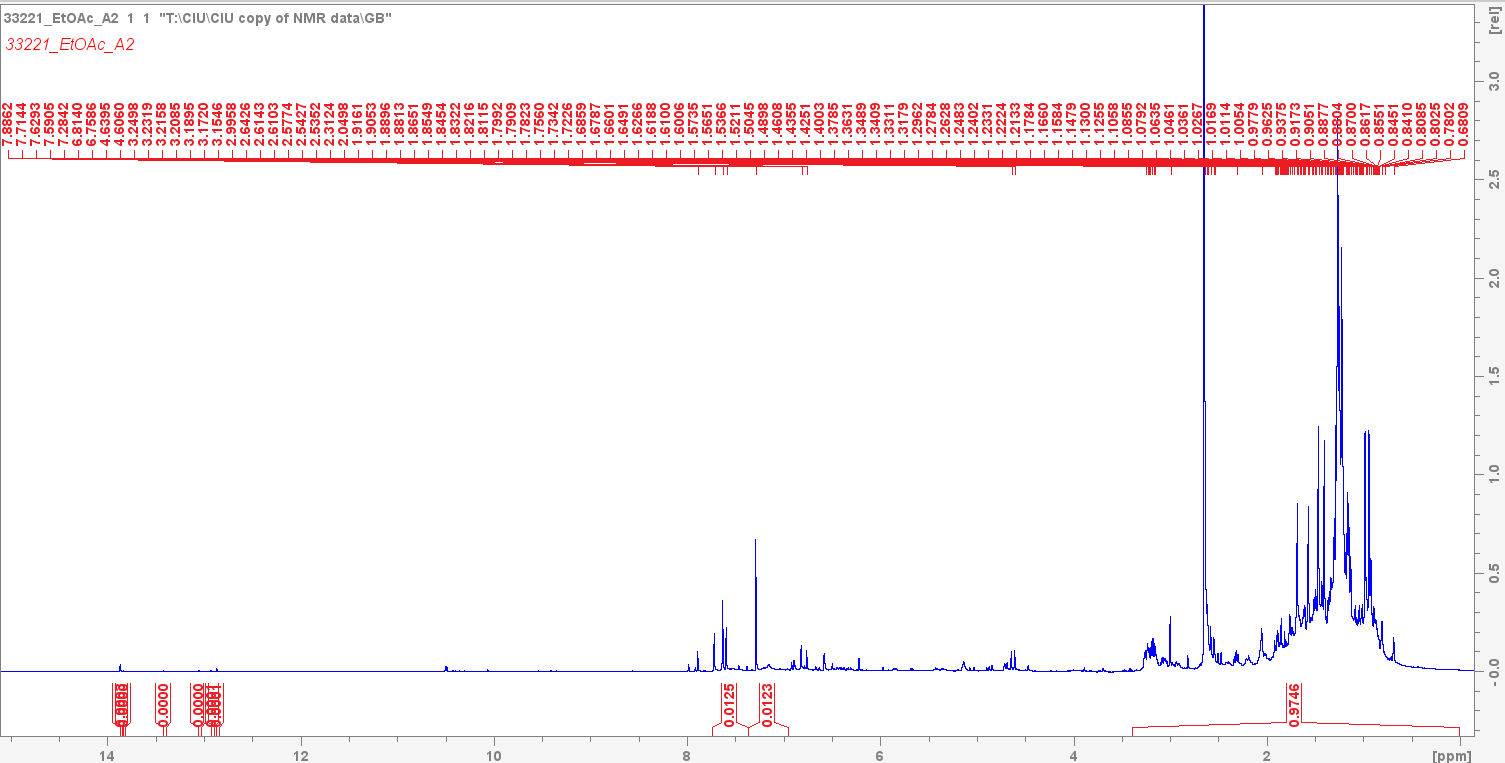


33221_E2


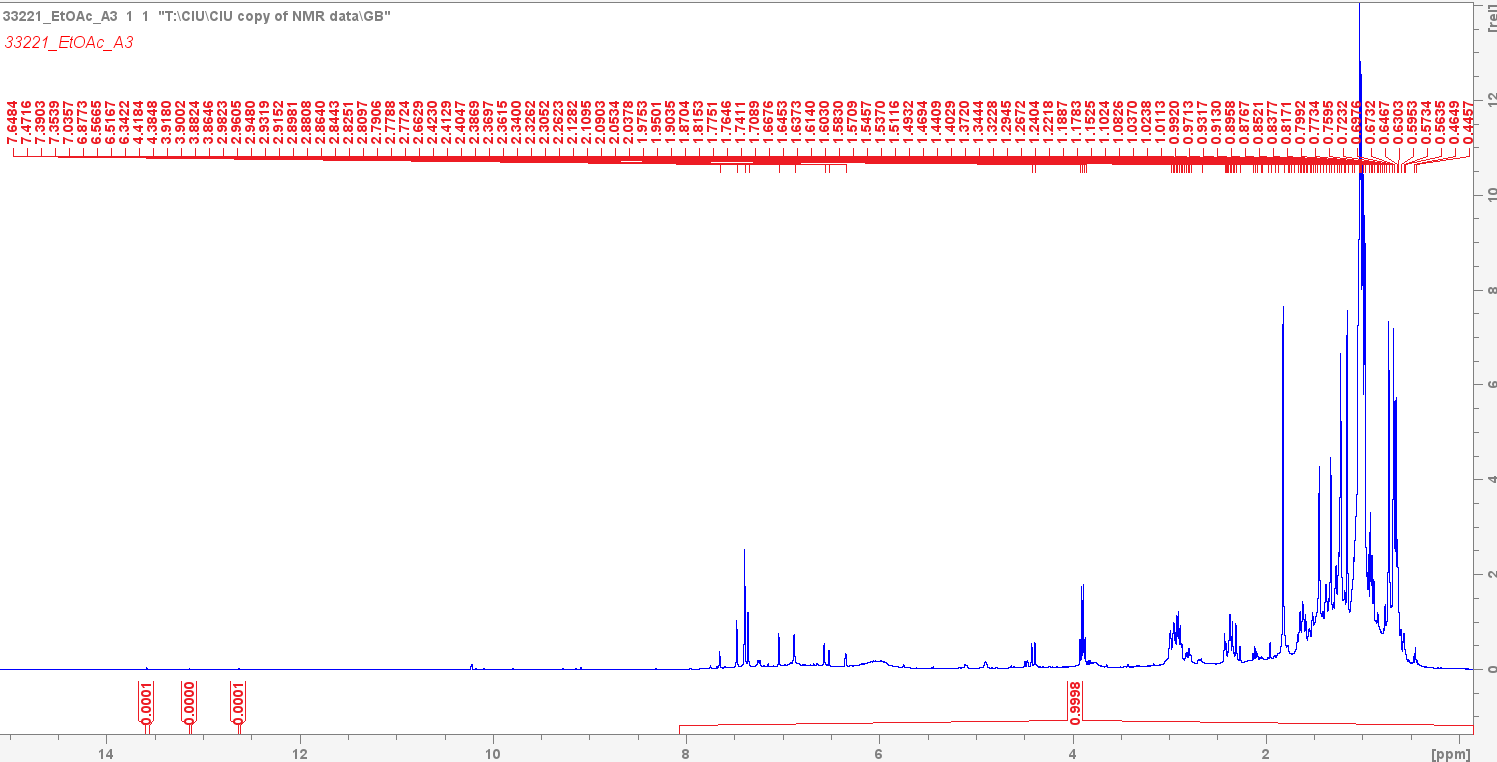


33221_E3


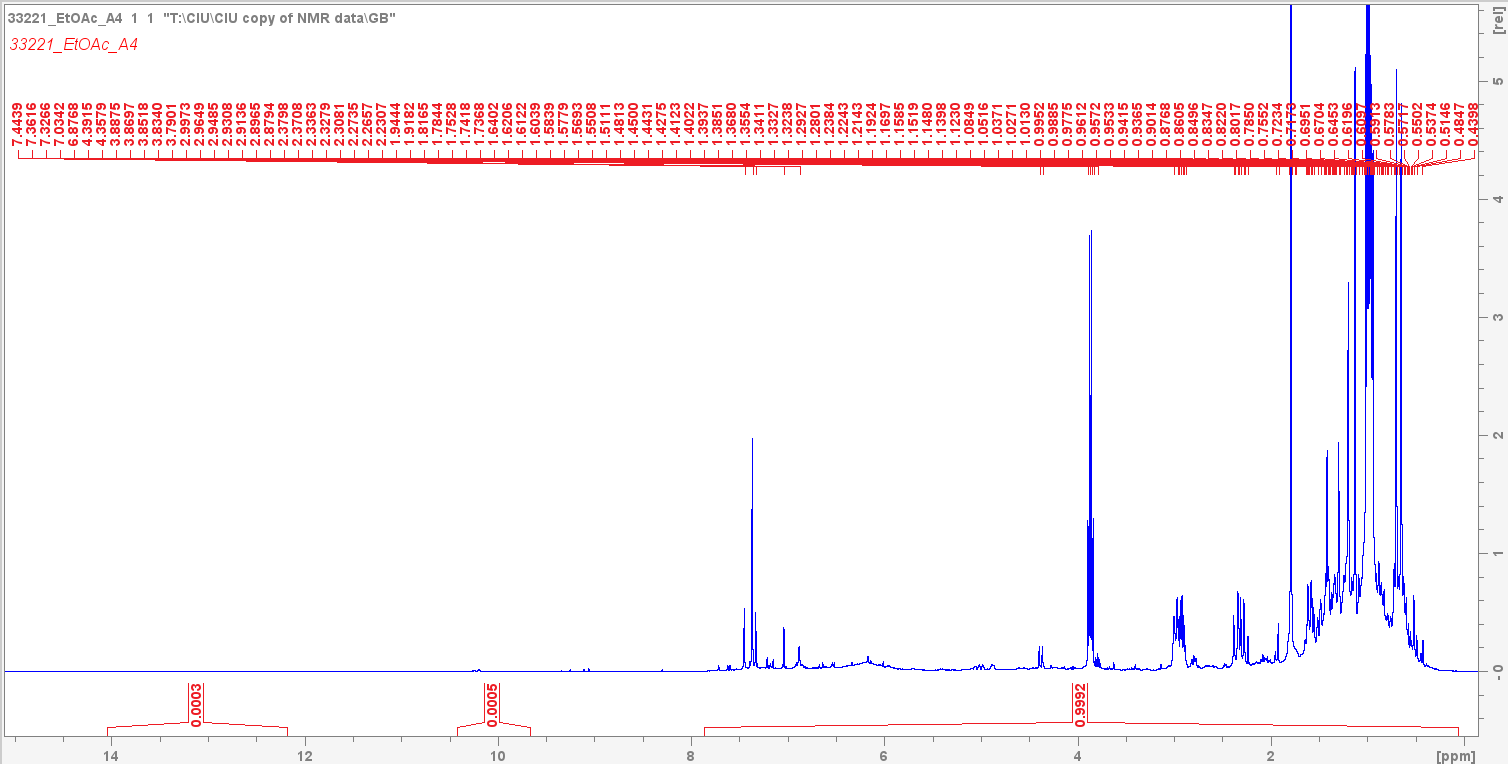


33221_E4


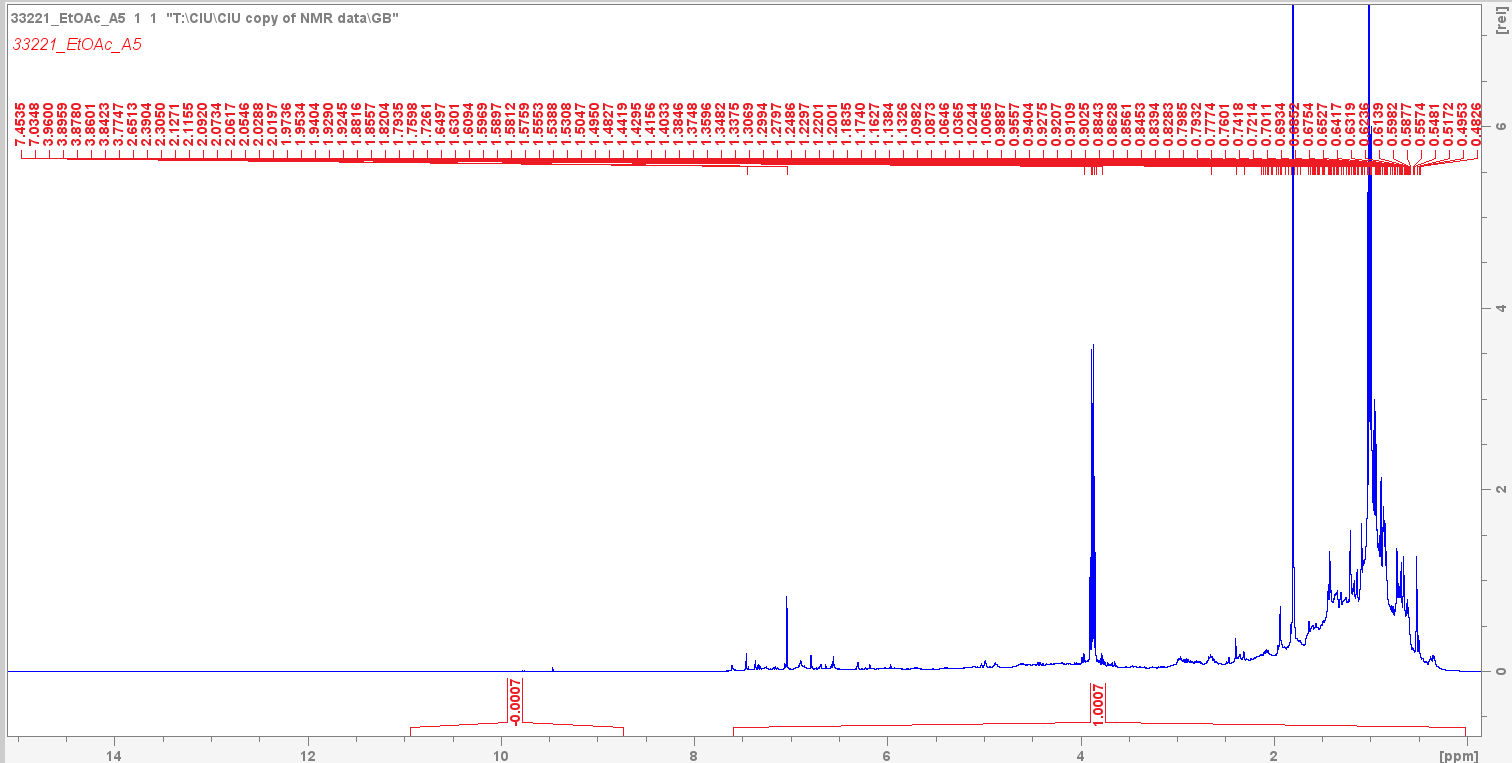


33221_E5


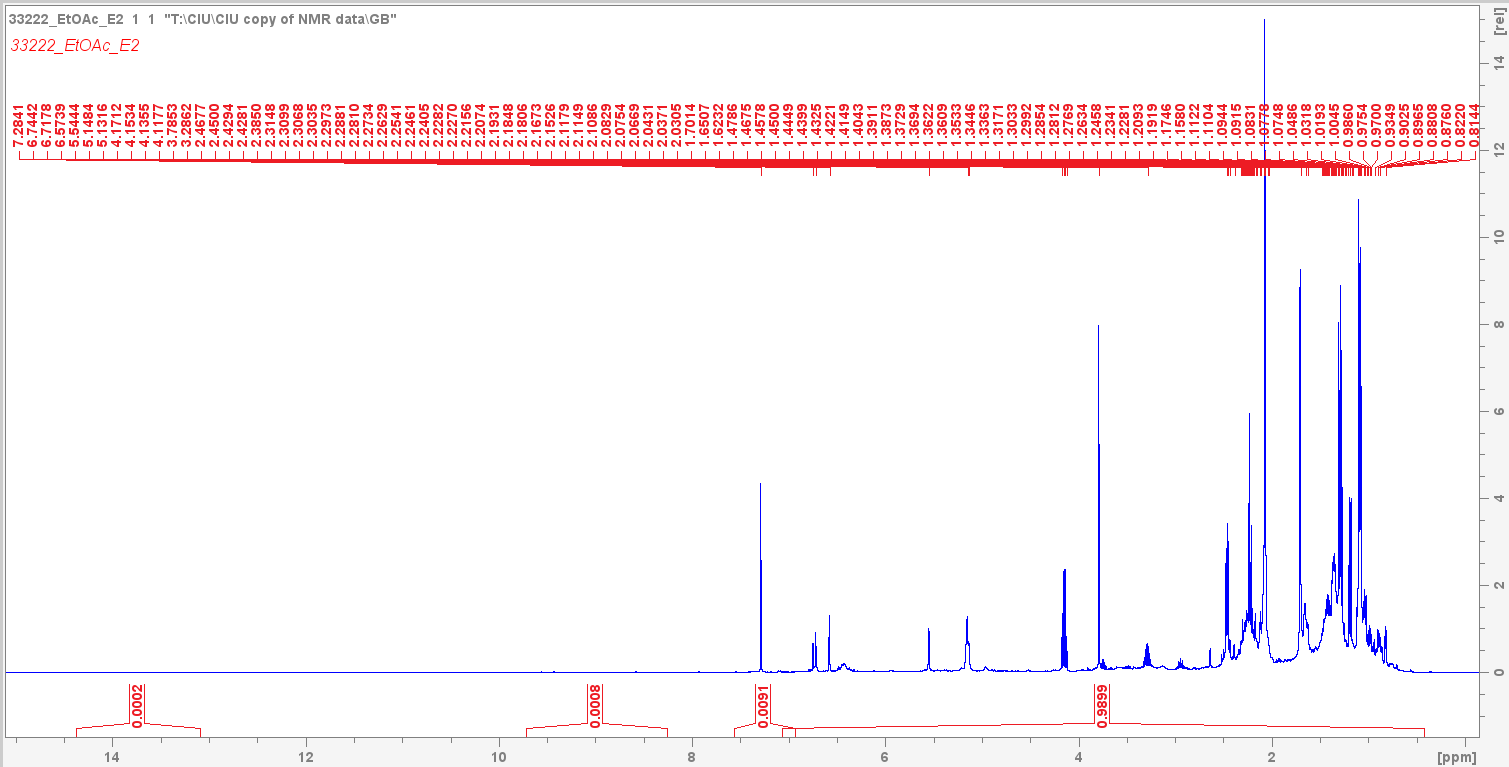


33222_E2


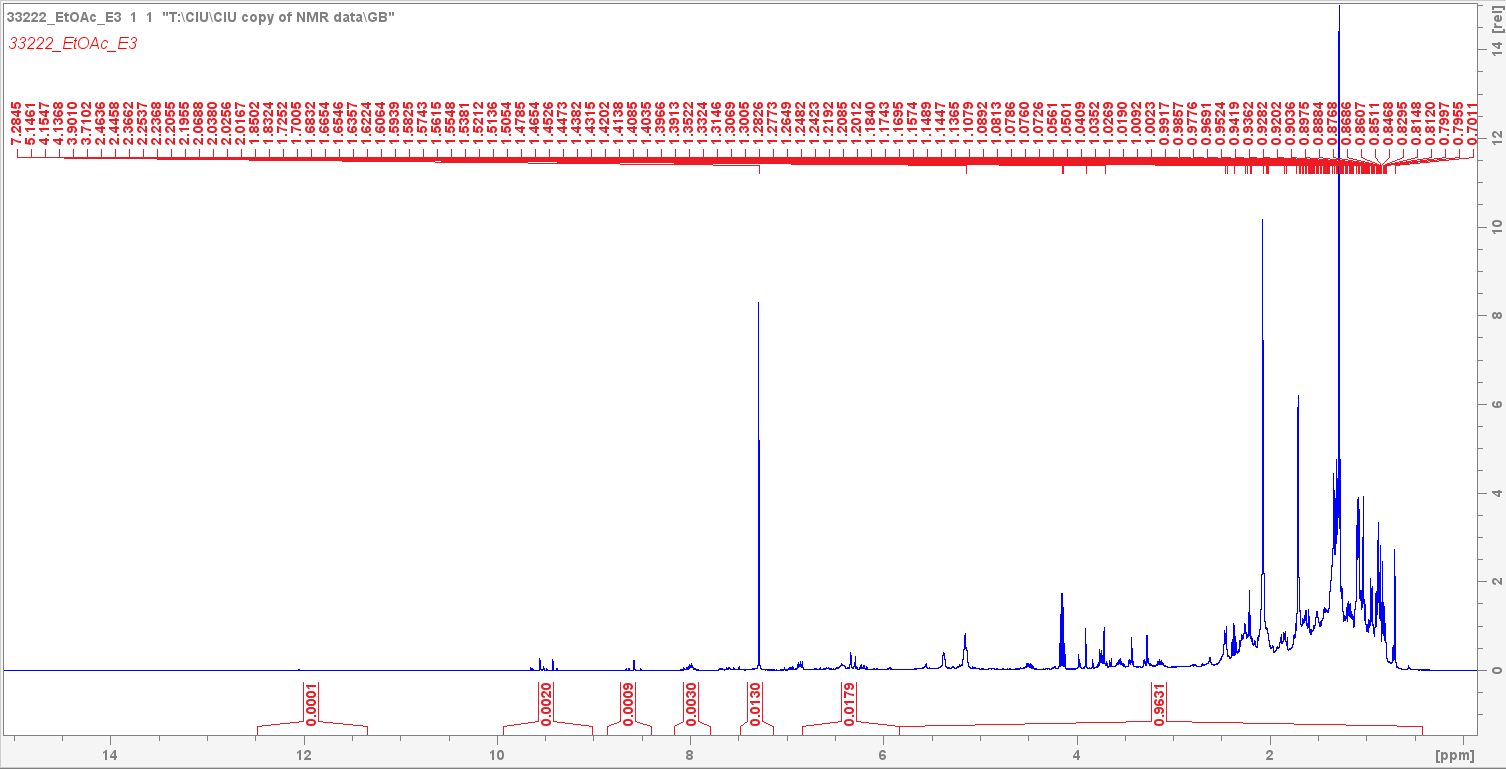


33222_E3


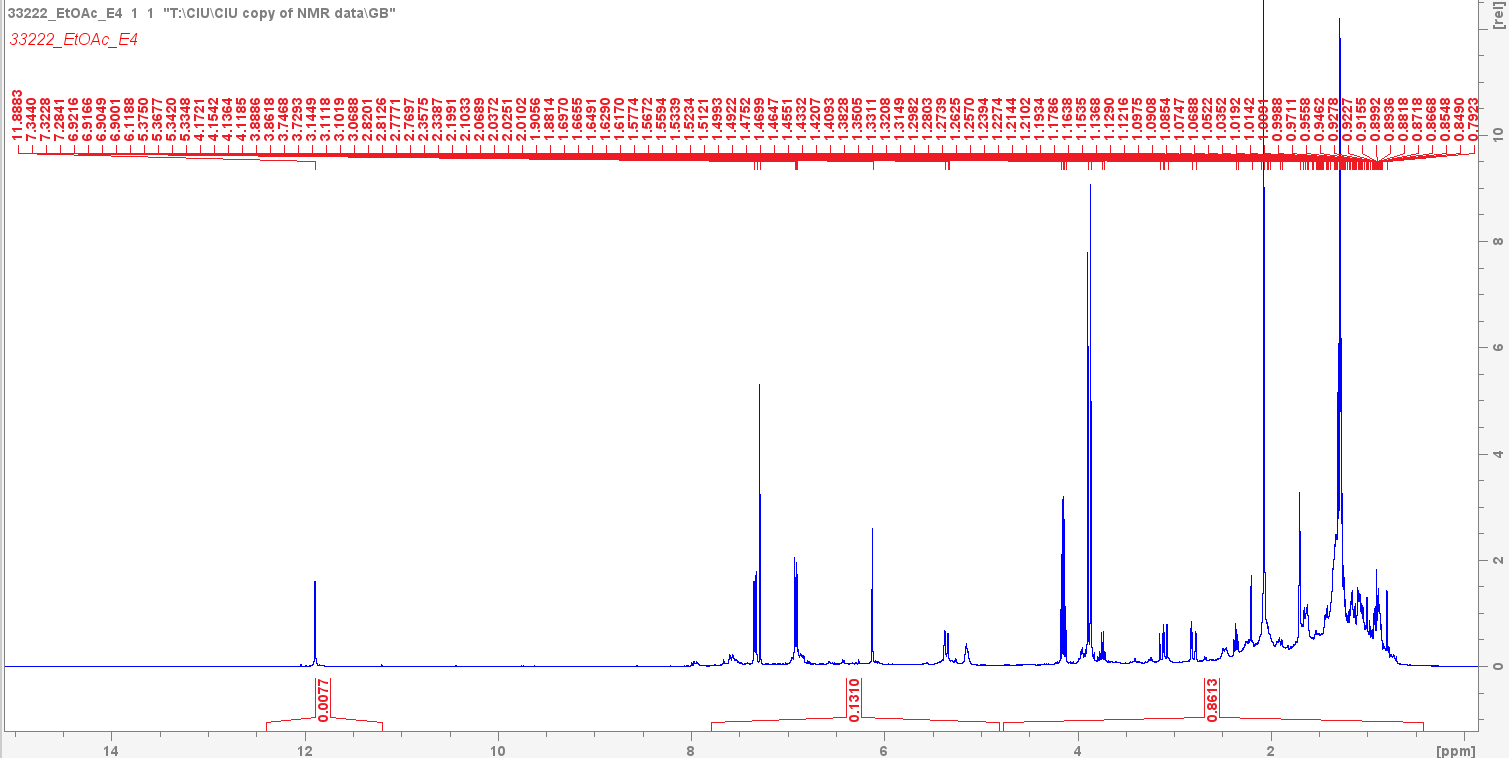


33222_E4


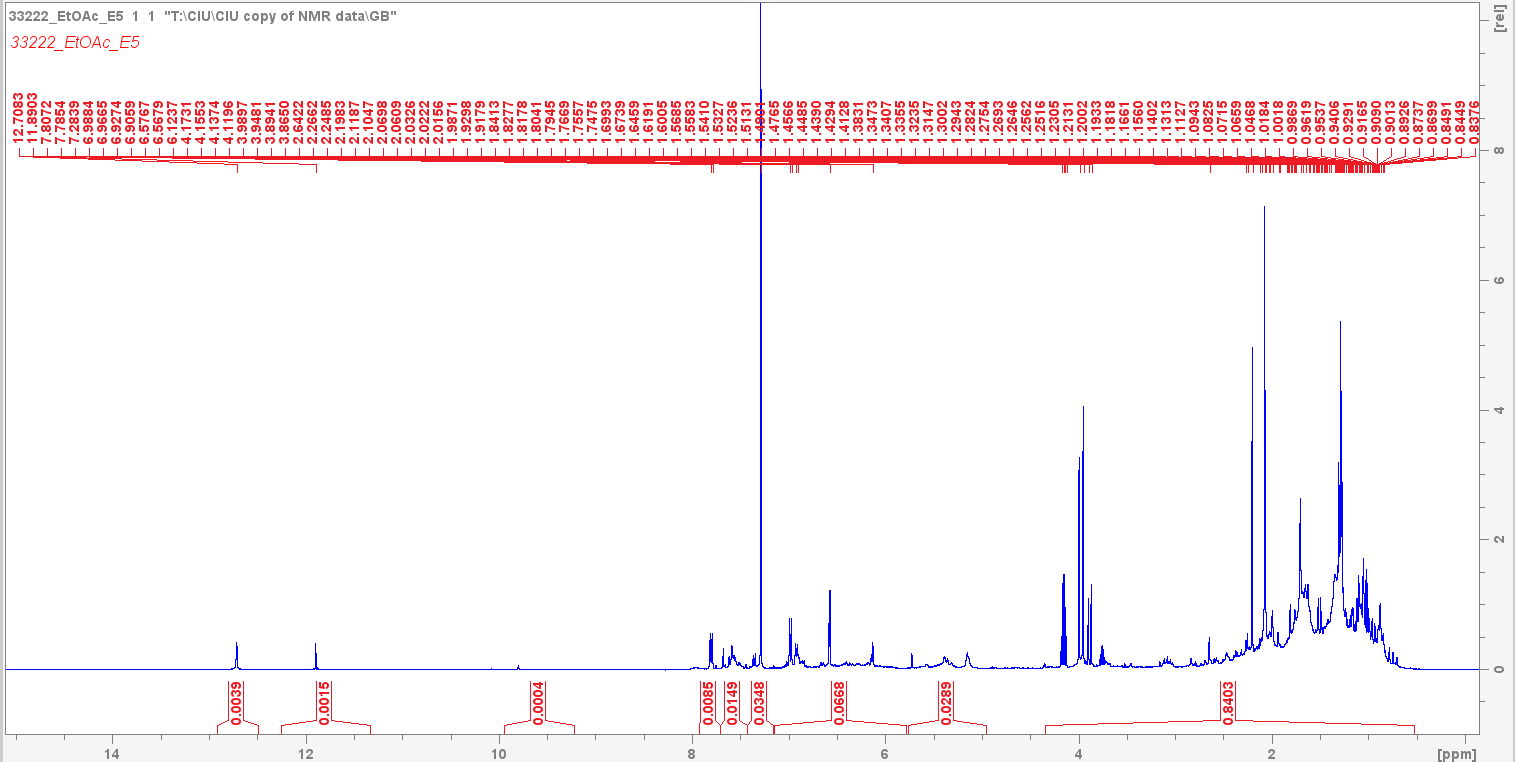


33222_E5


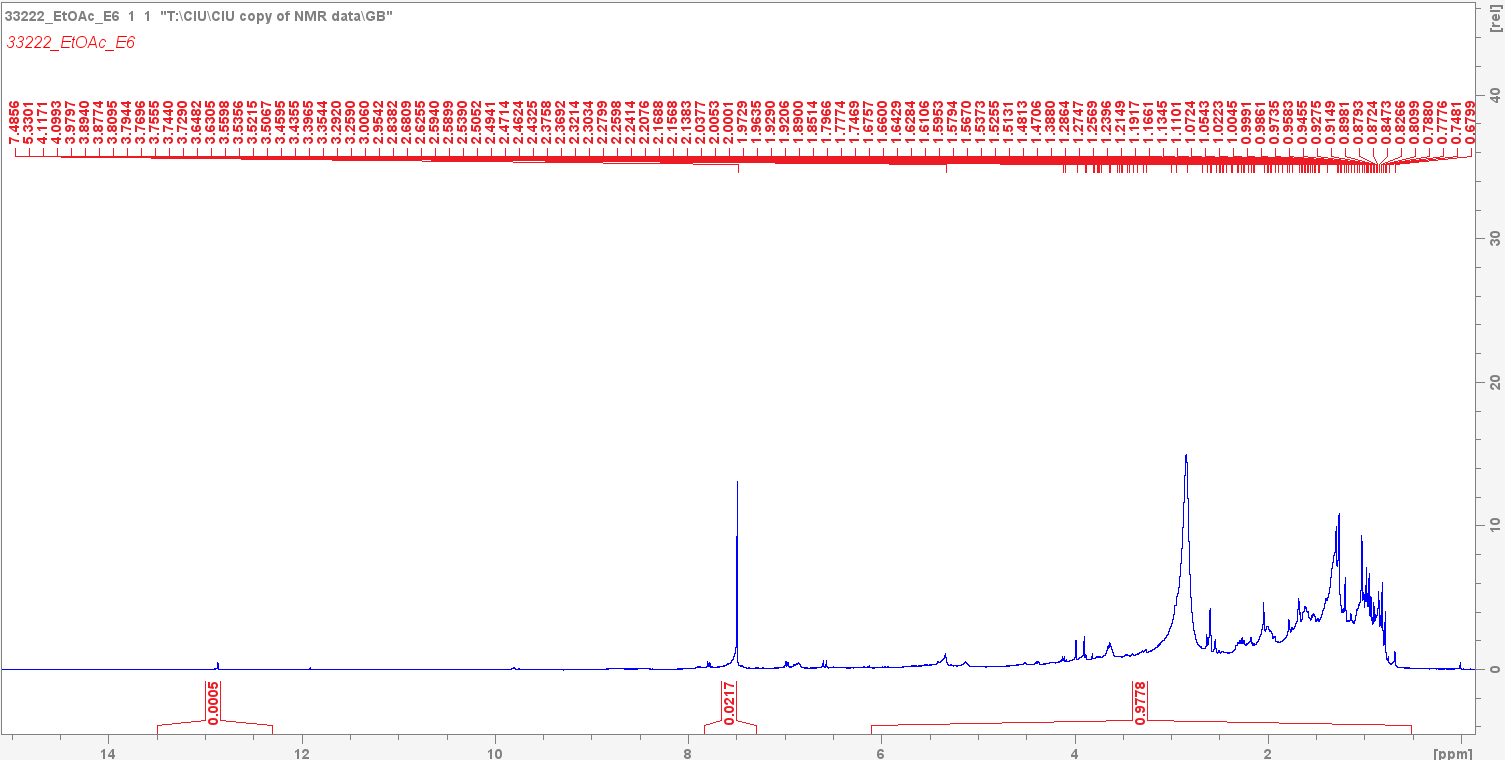


33222_E6


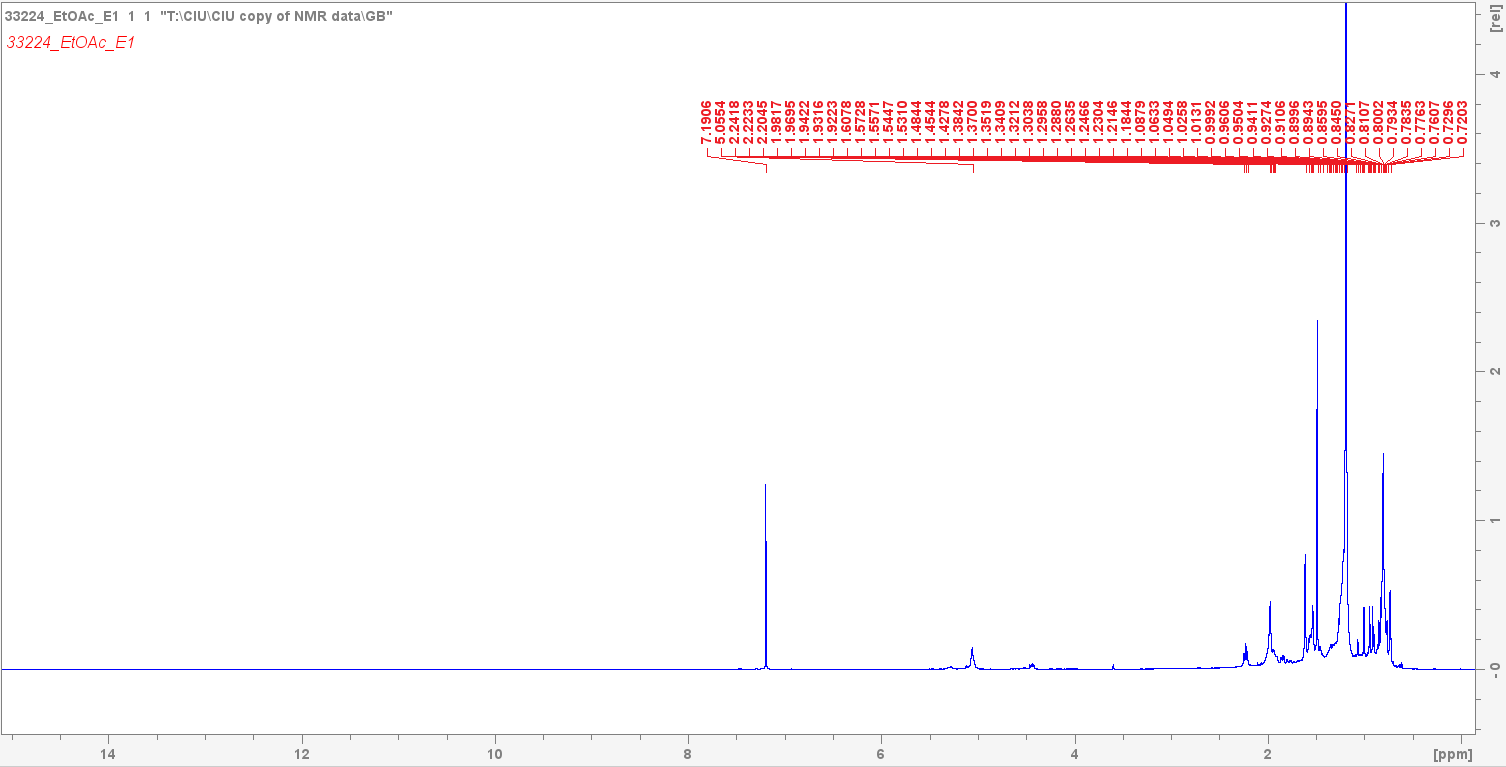


33224_E1


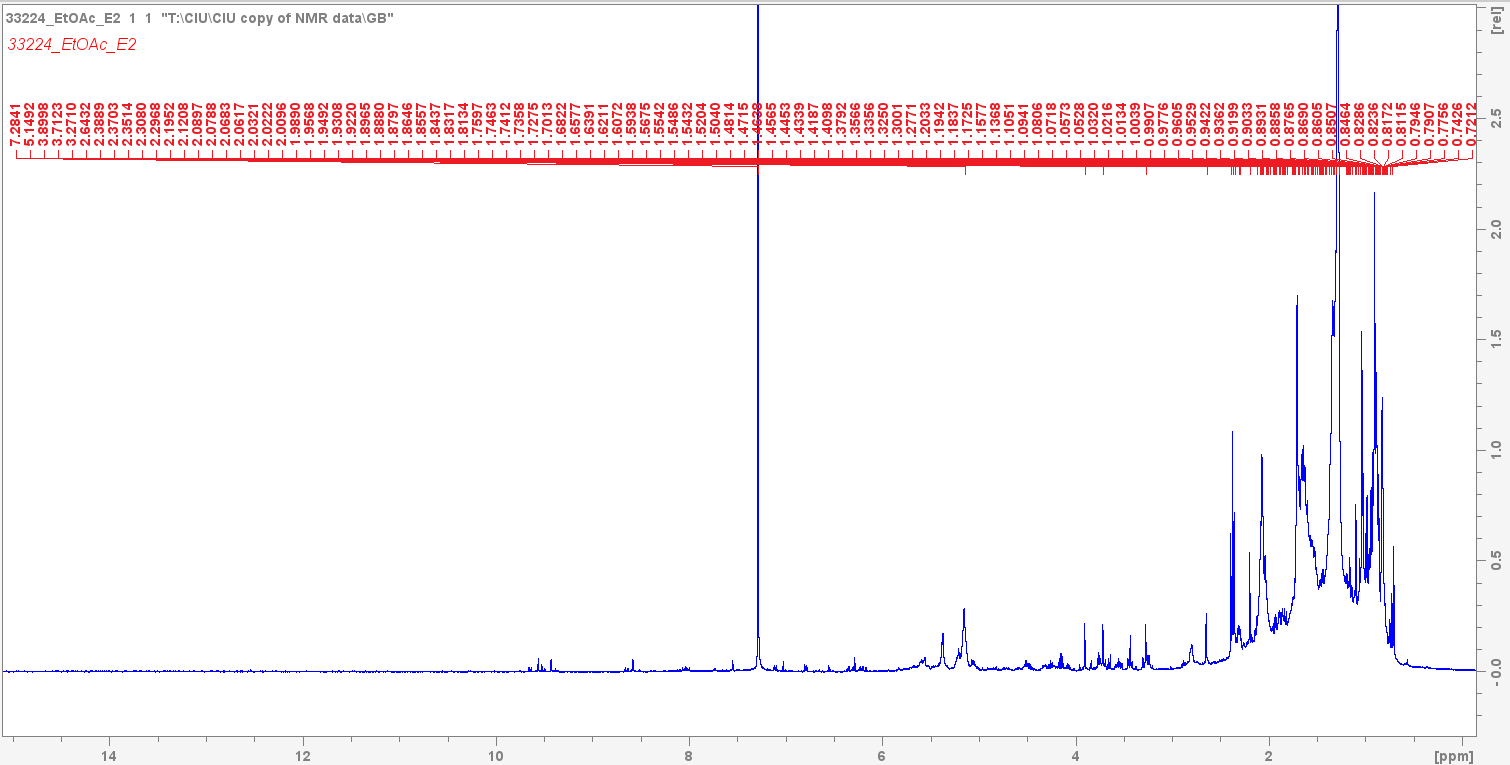


33224_E2


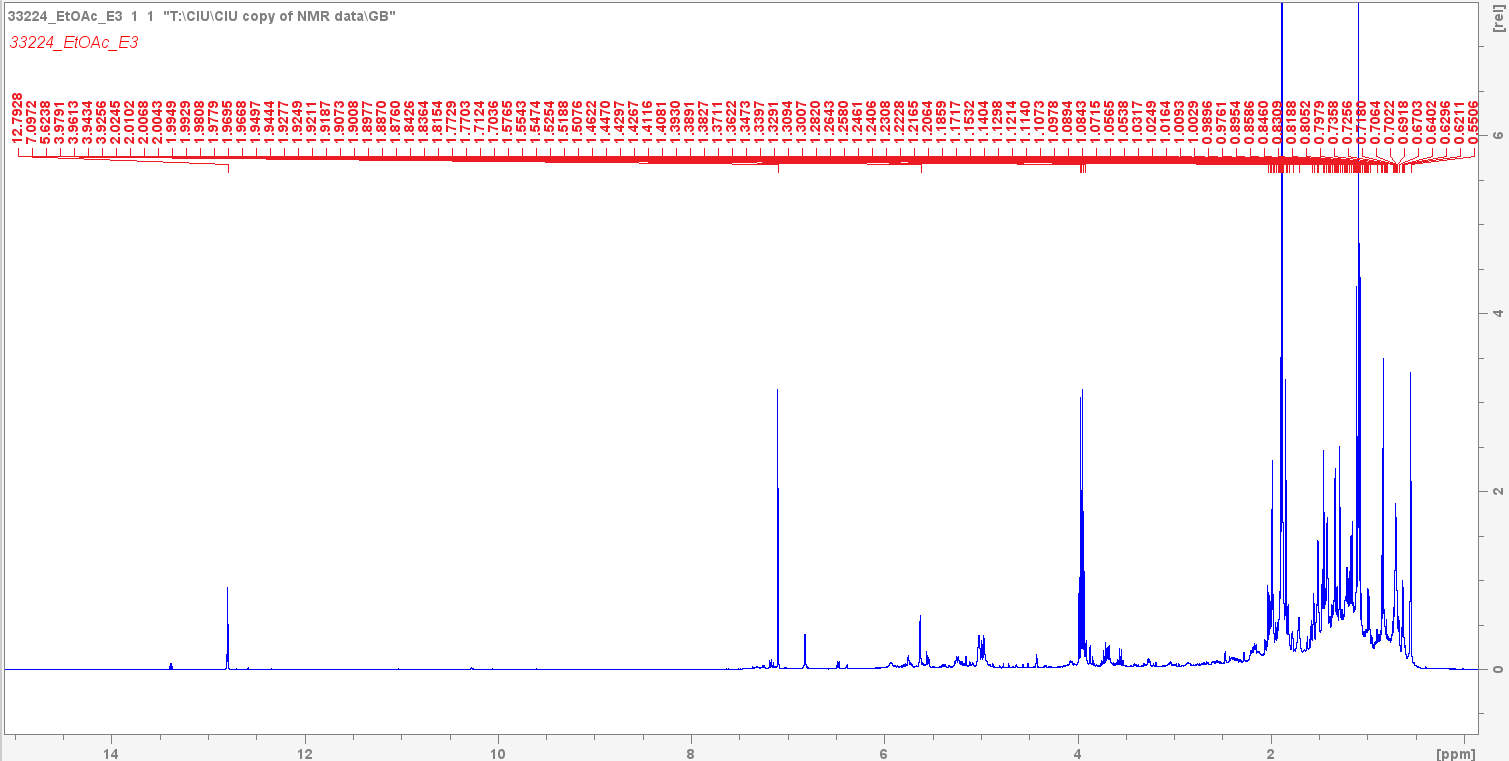


33224_E3


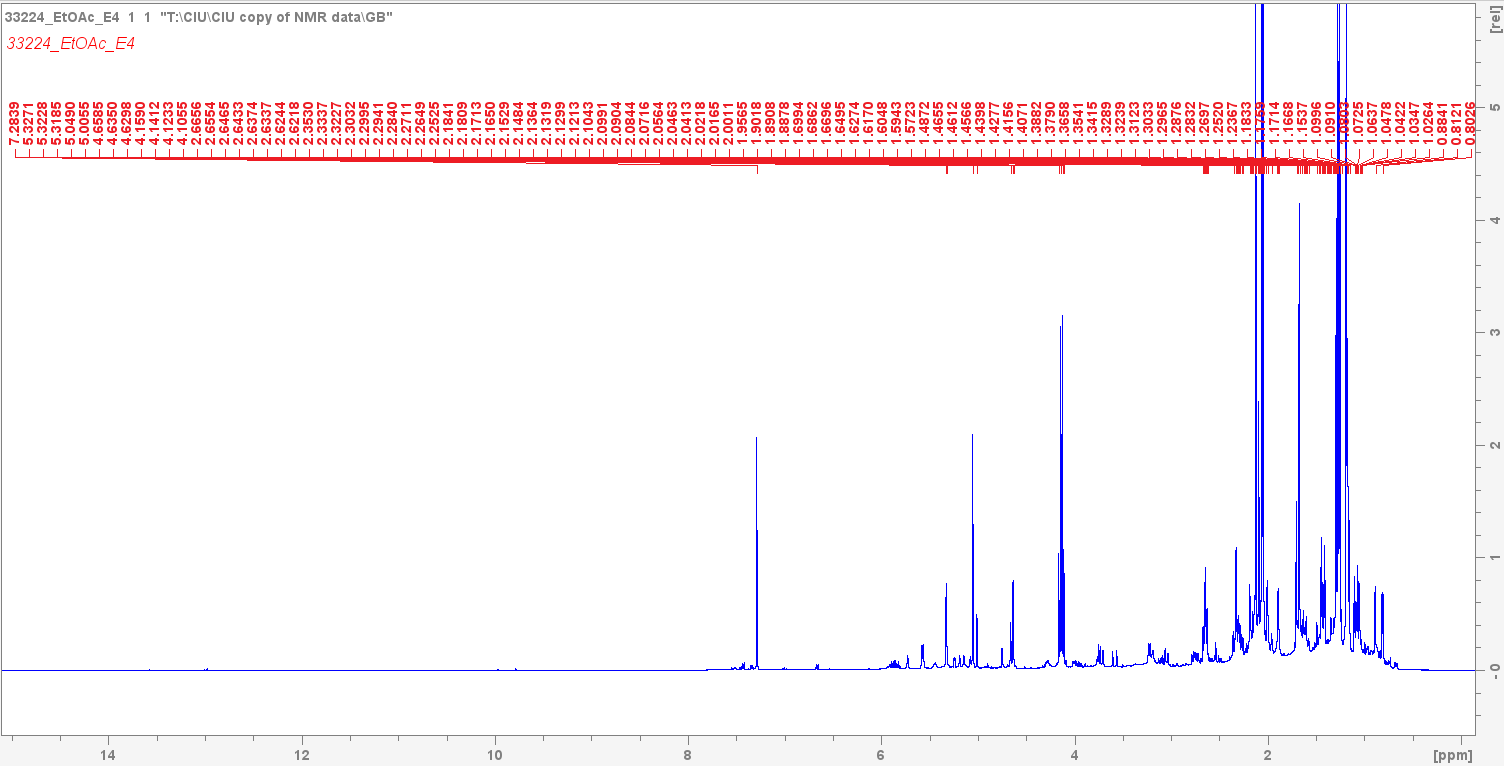


33224_E4


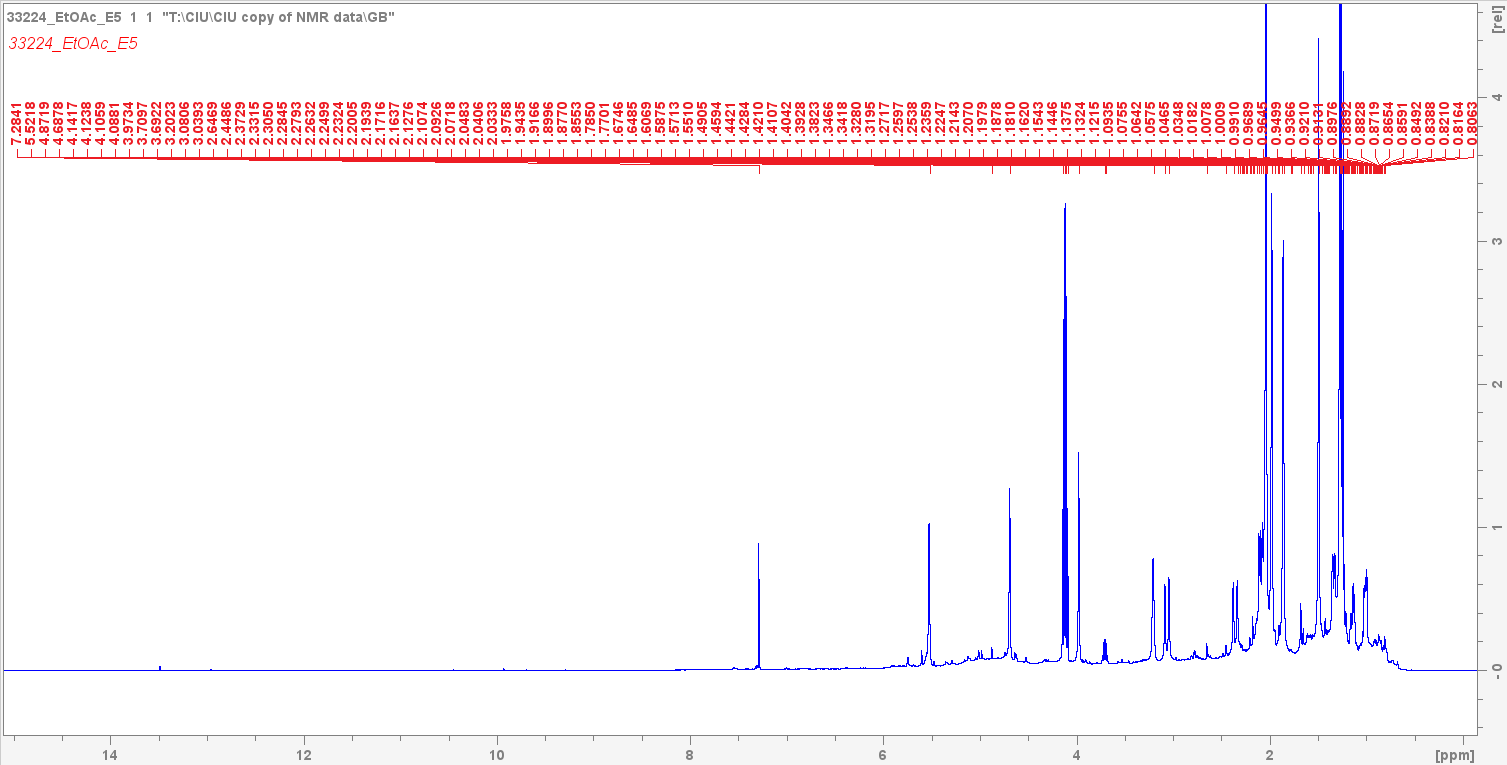


33224_E5


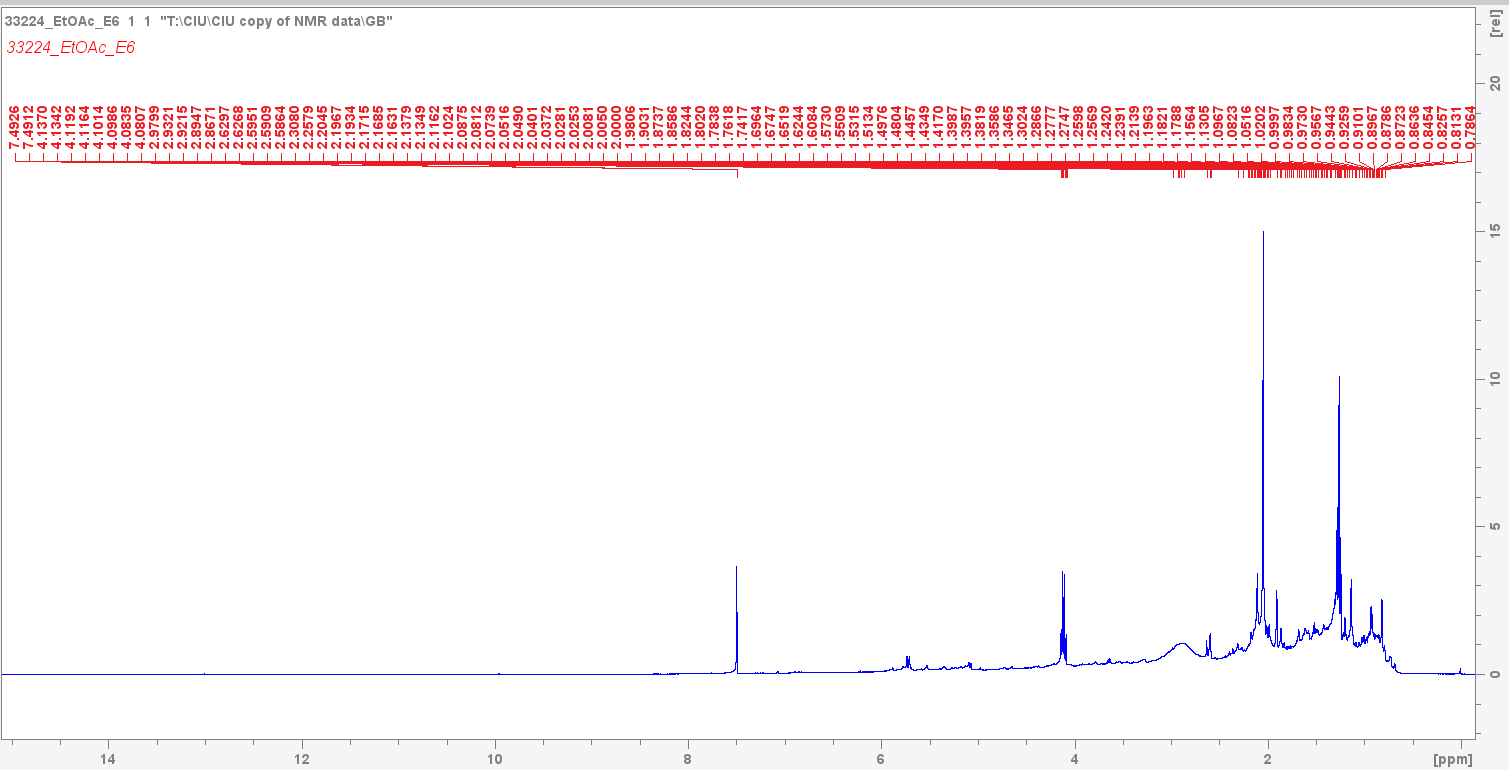
33224_E6


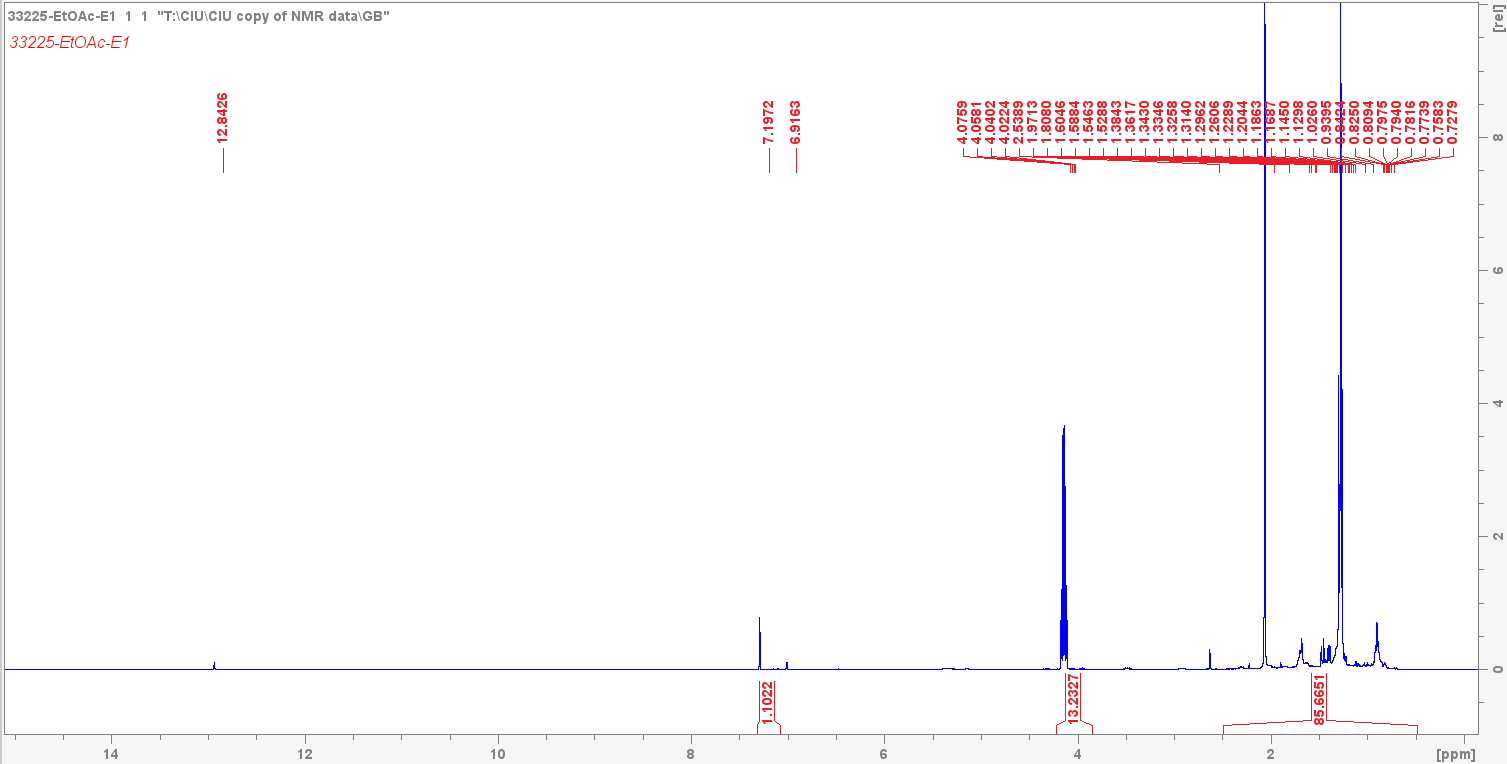


33225_E1


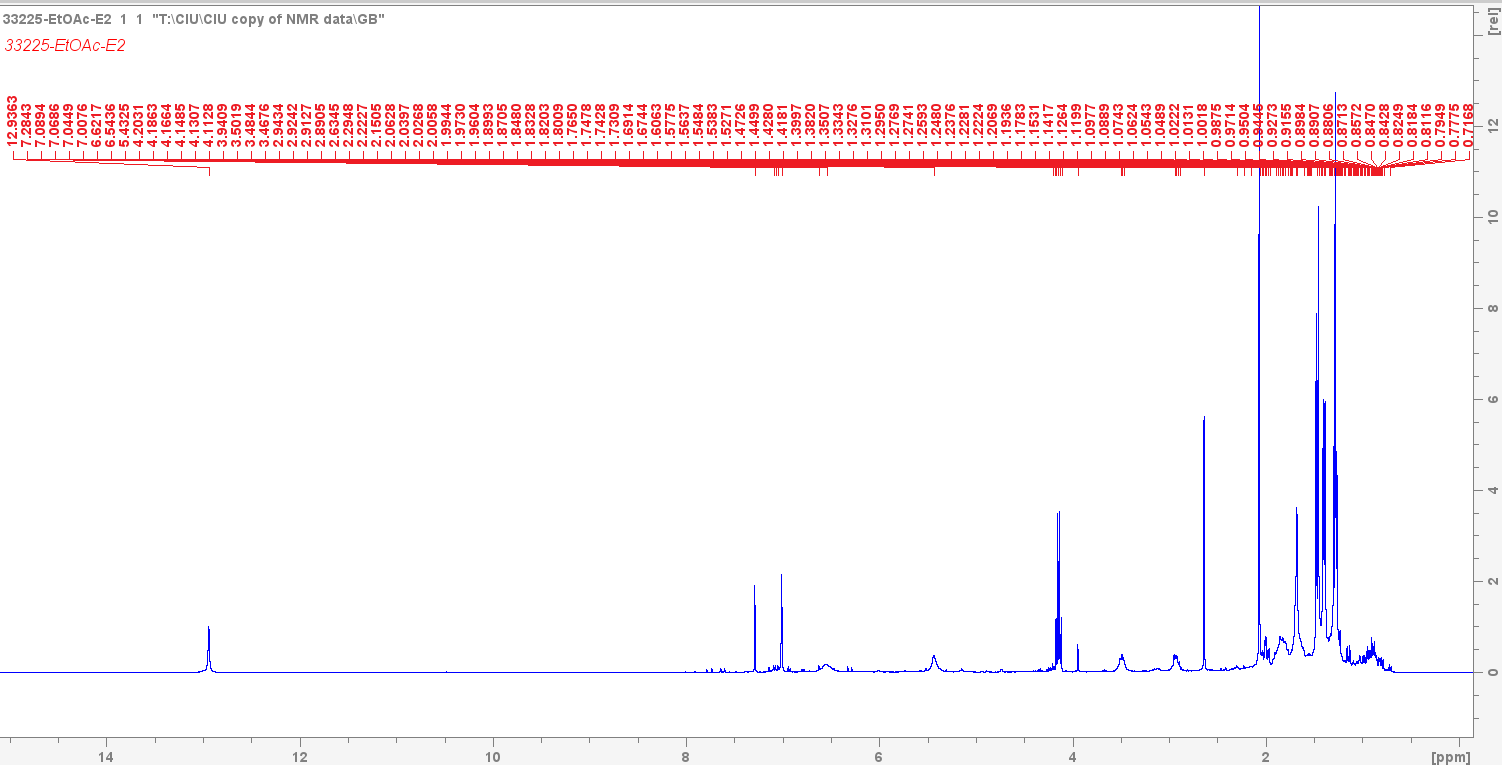


33225_E2


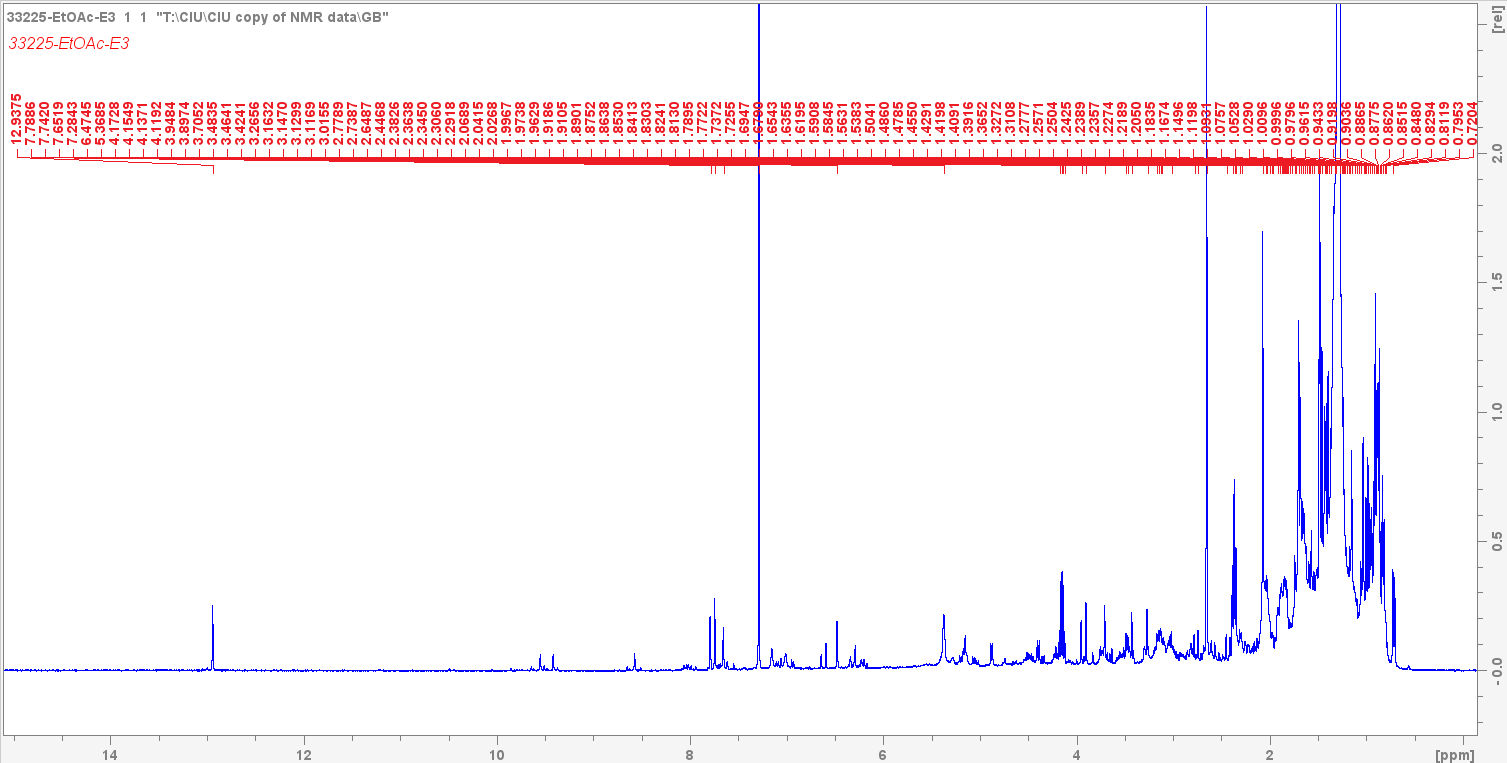


33225_E3


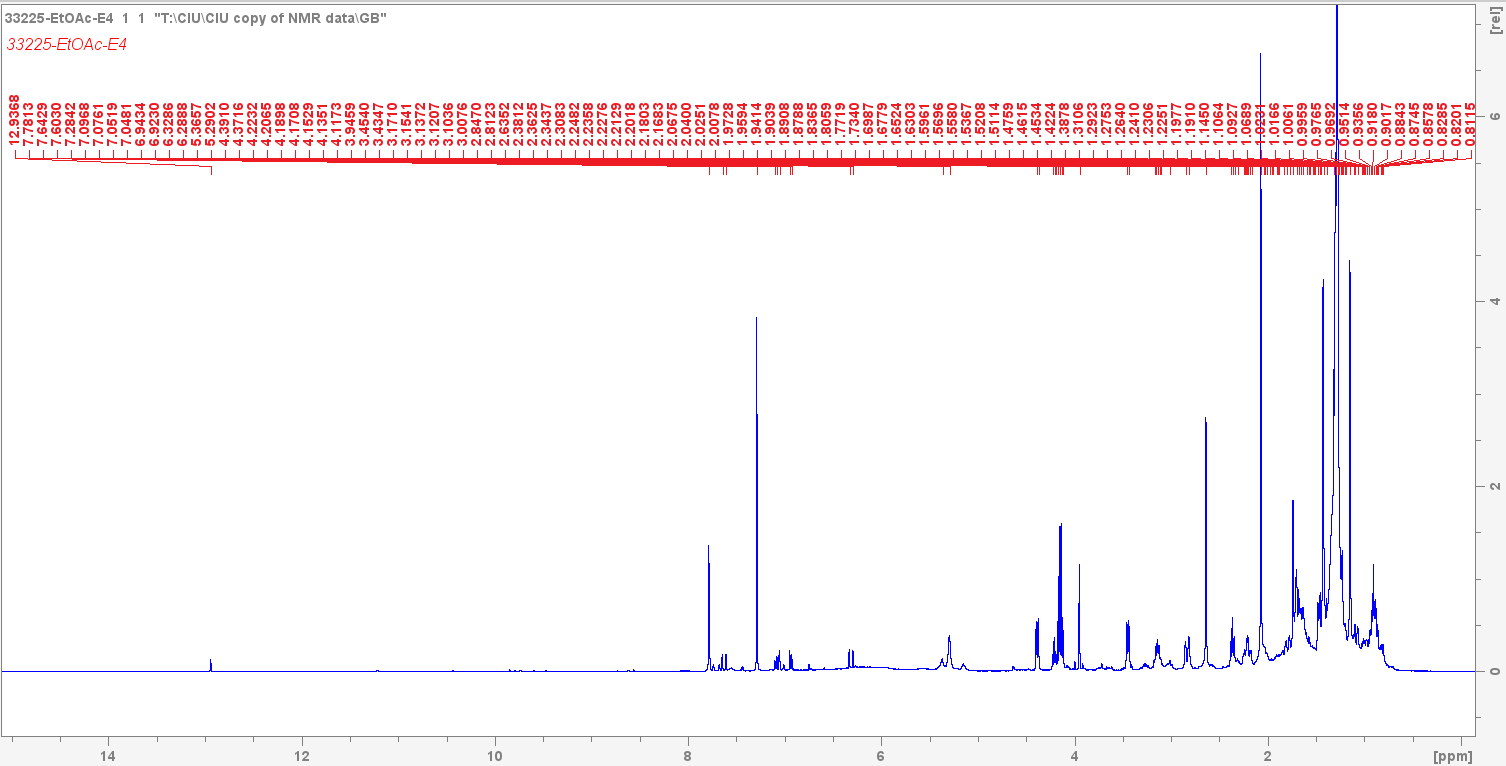


33225_E4


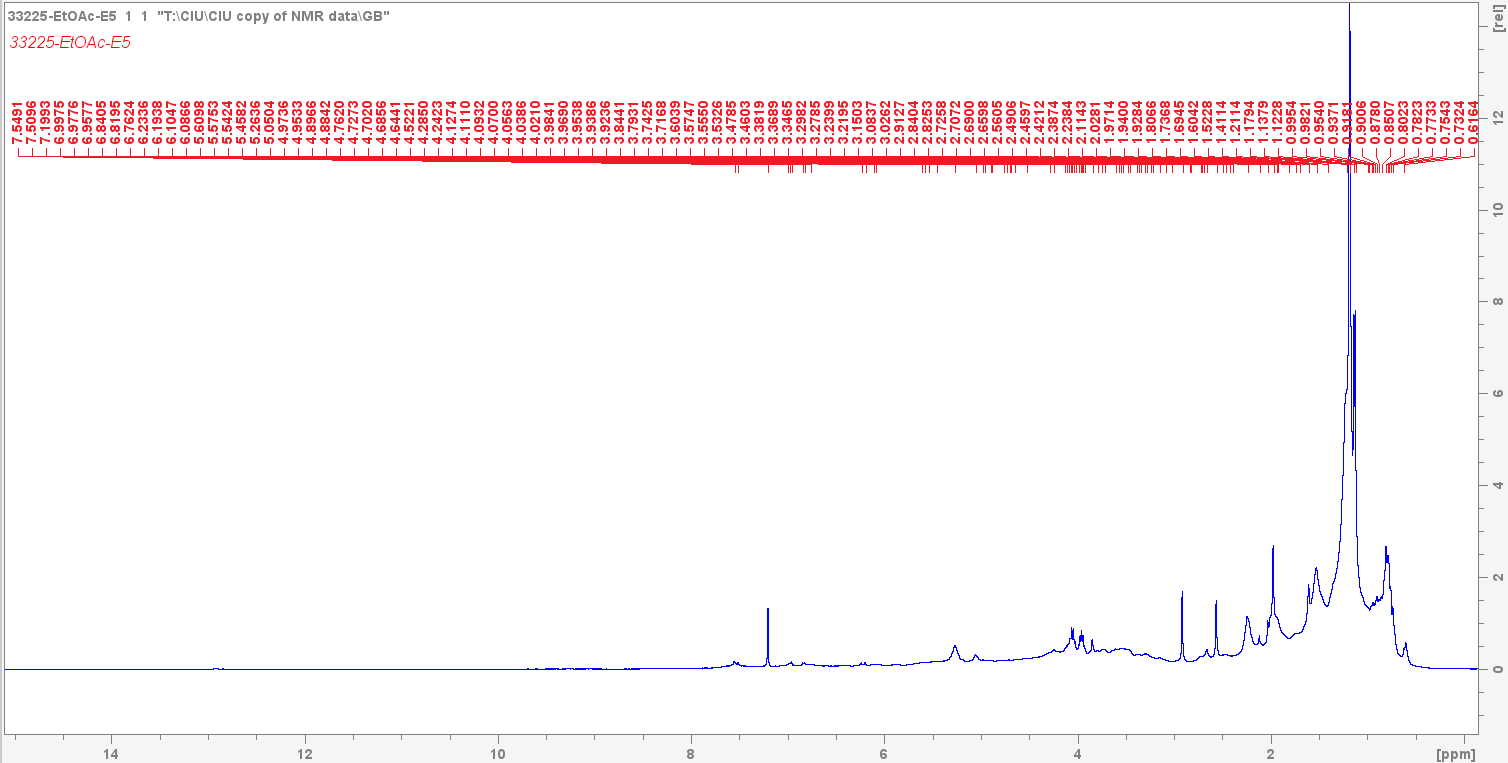


33225_E5


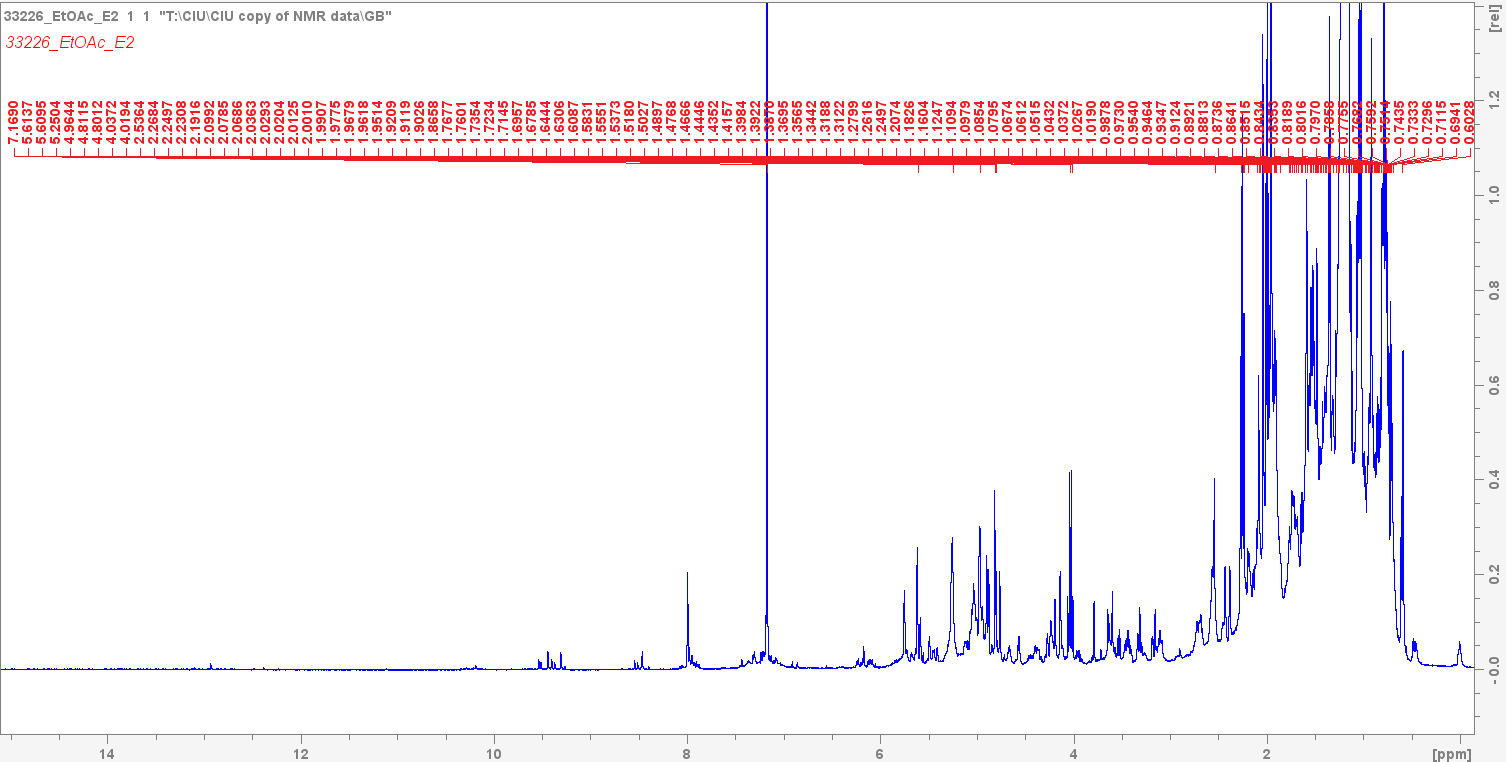


33226_E2


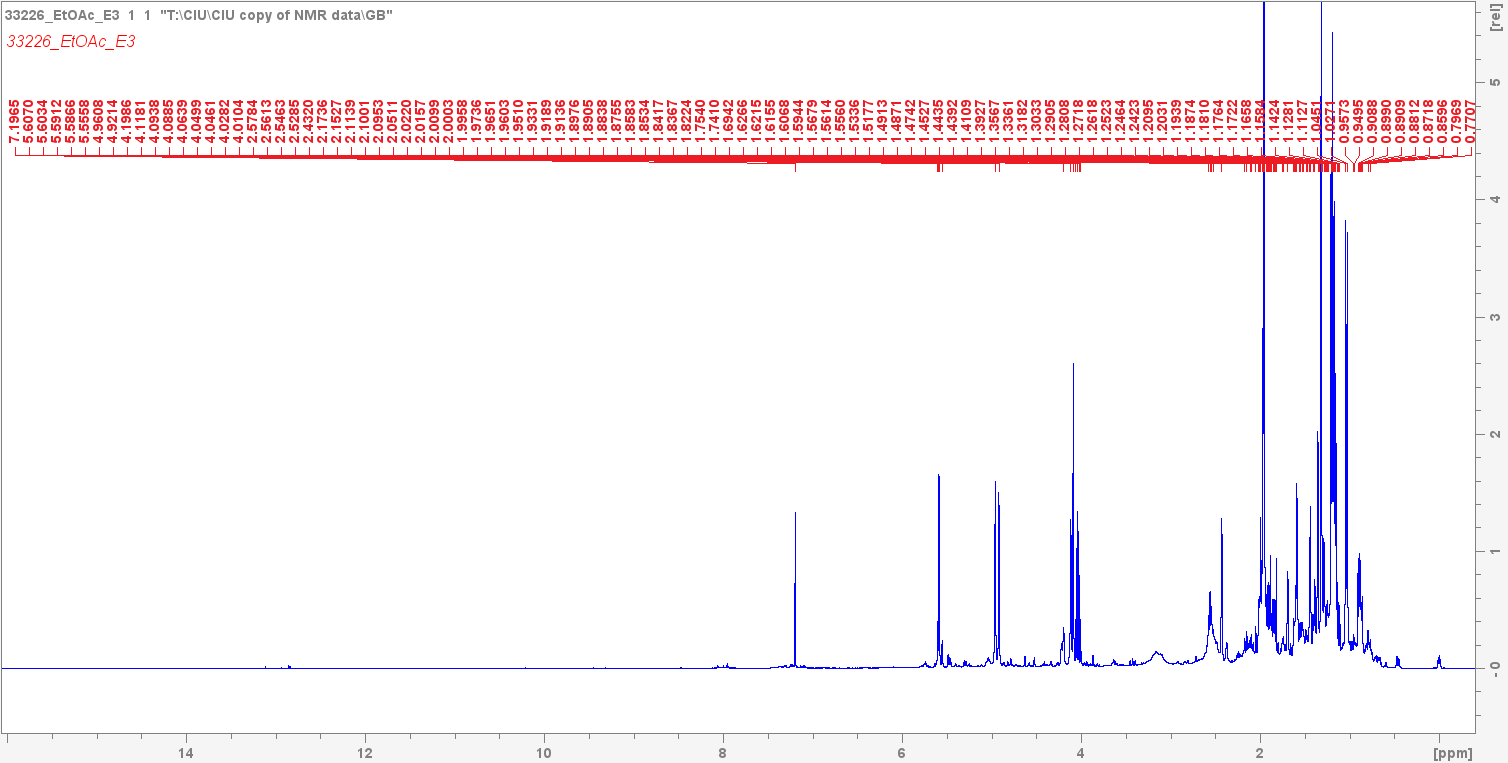


33226_E3


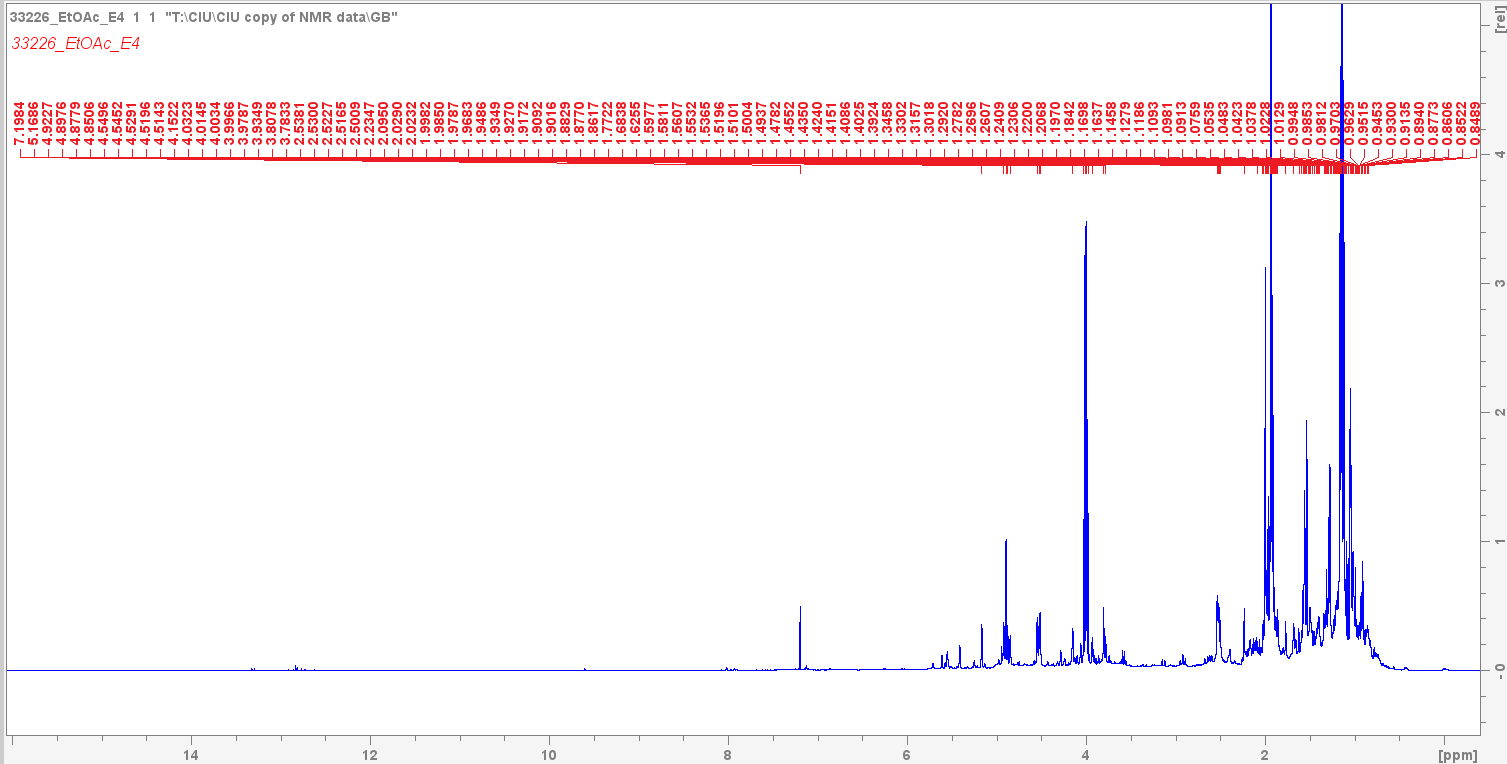


33226_E4


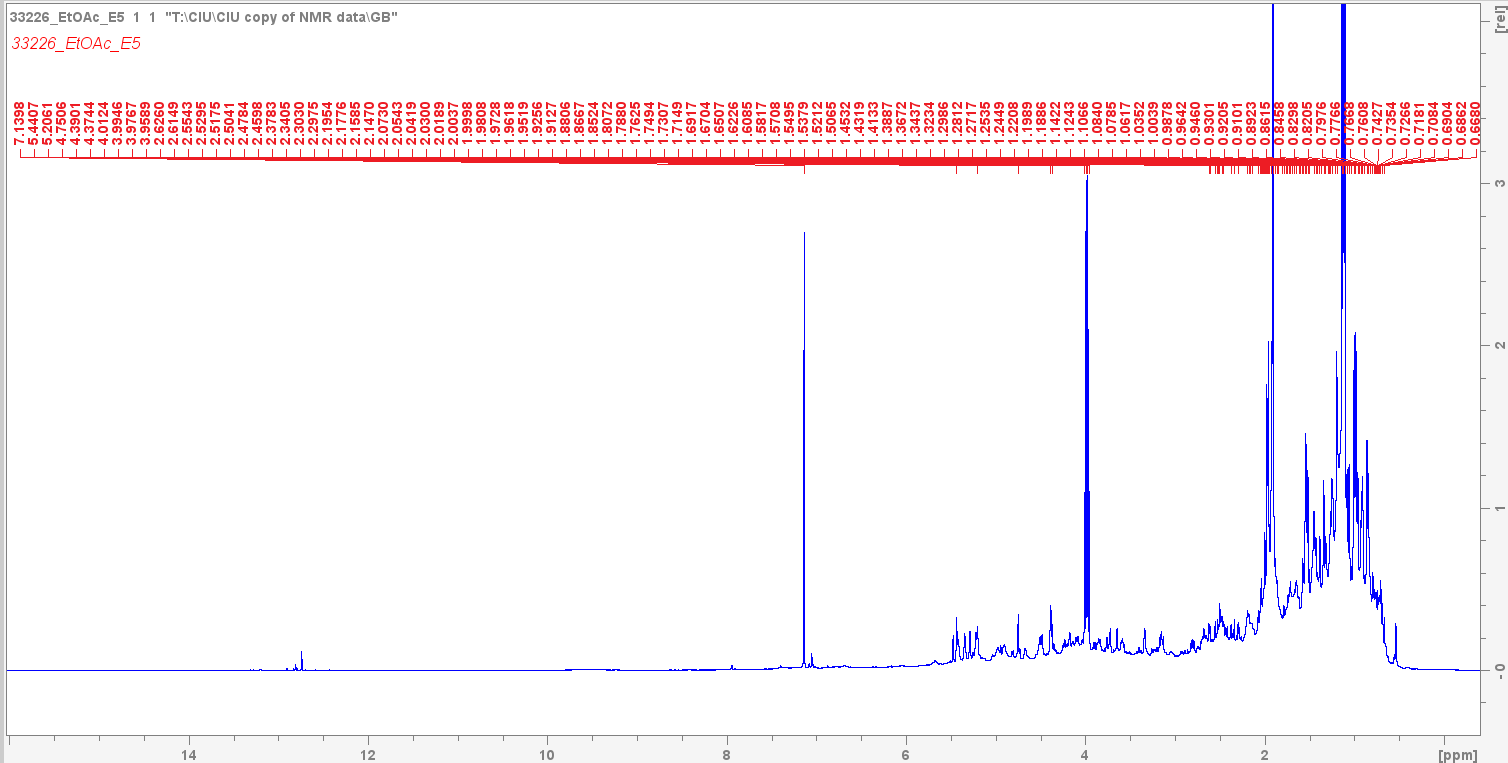


33226_E5


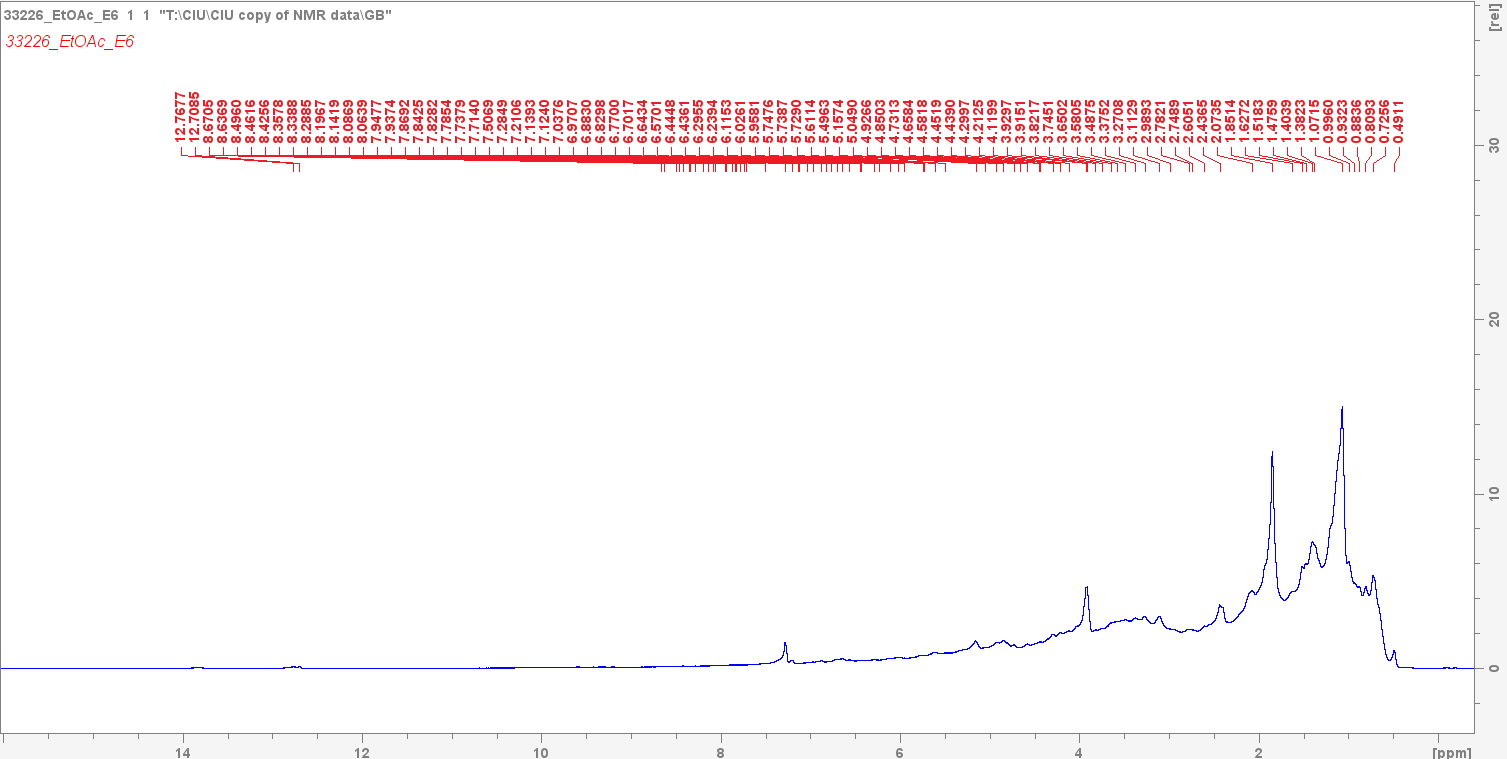


33226_E6


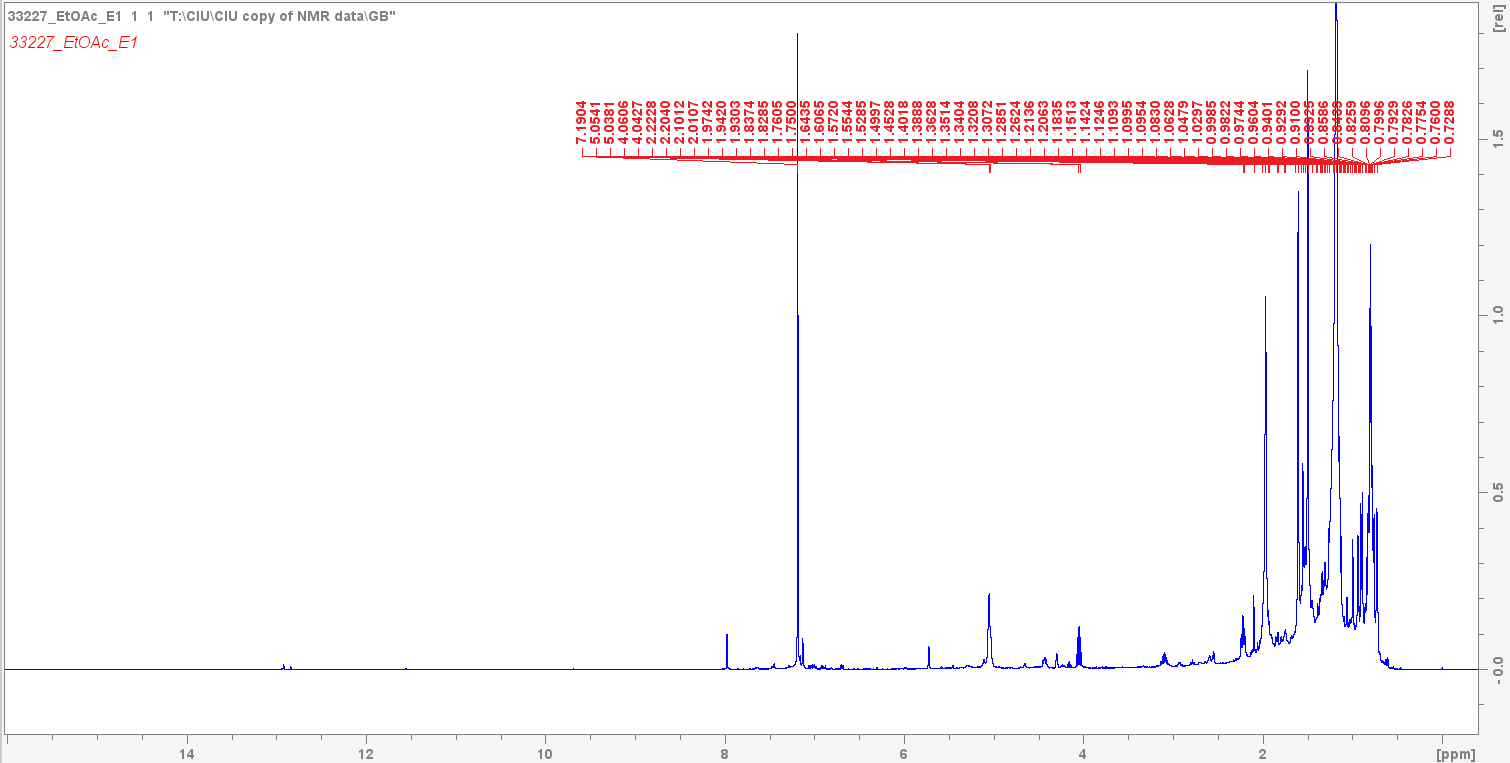


33227_E1


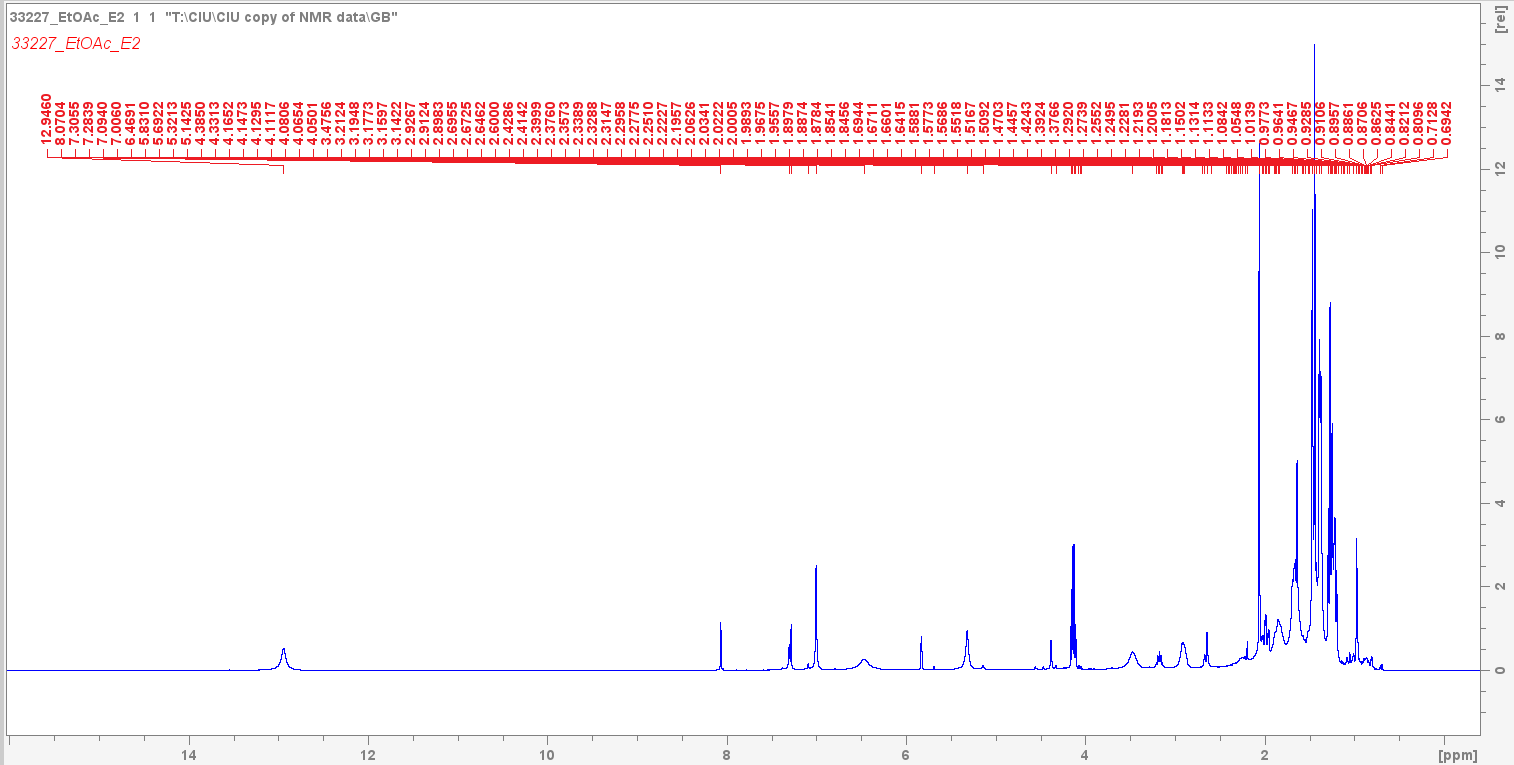


33227_E2


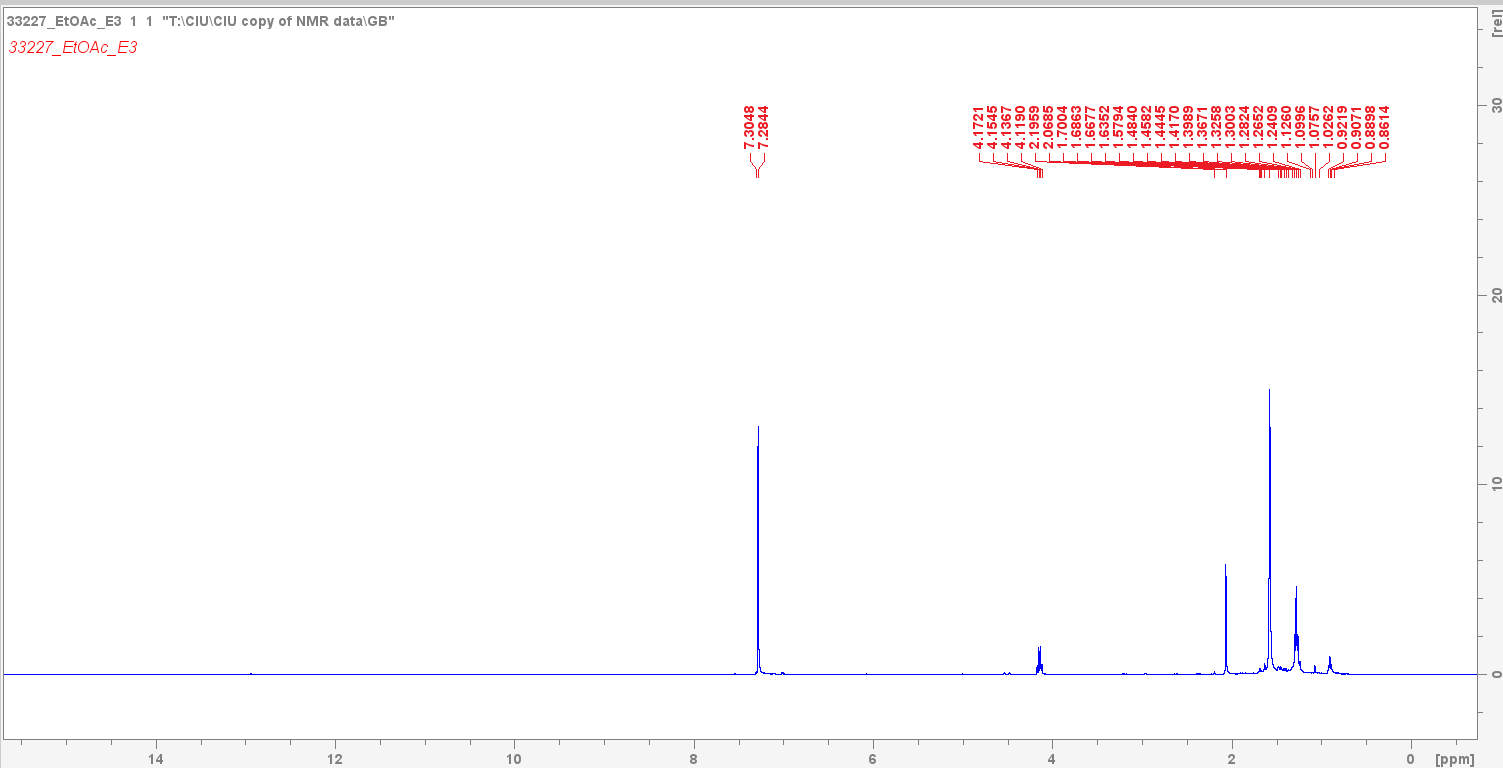


33227_E3


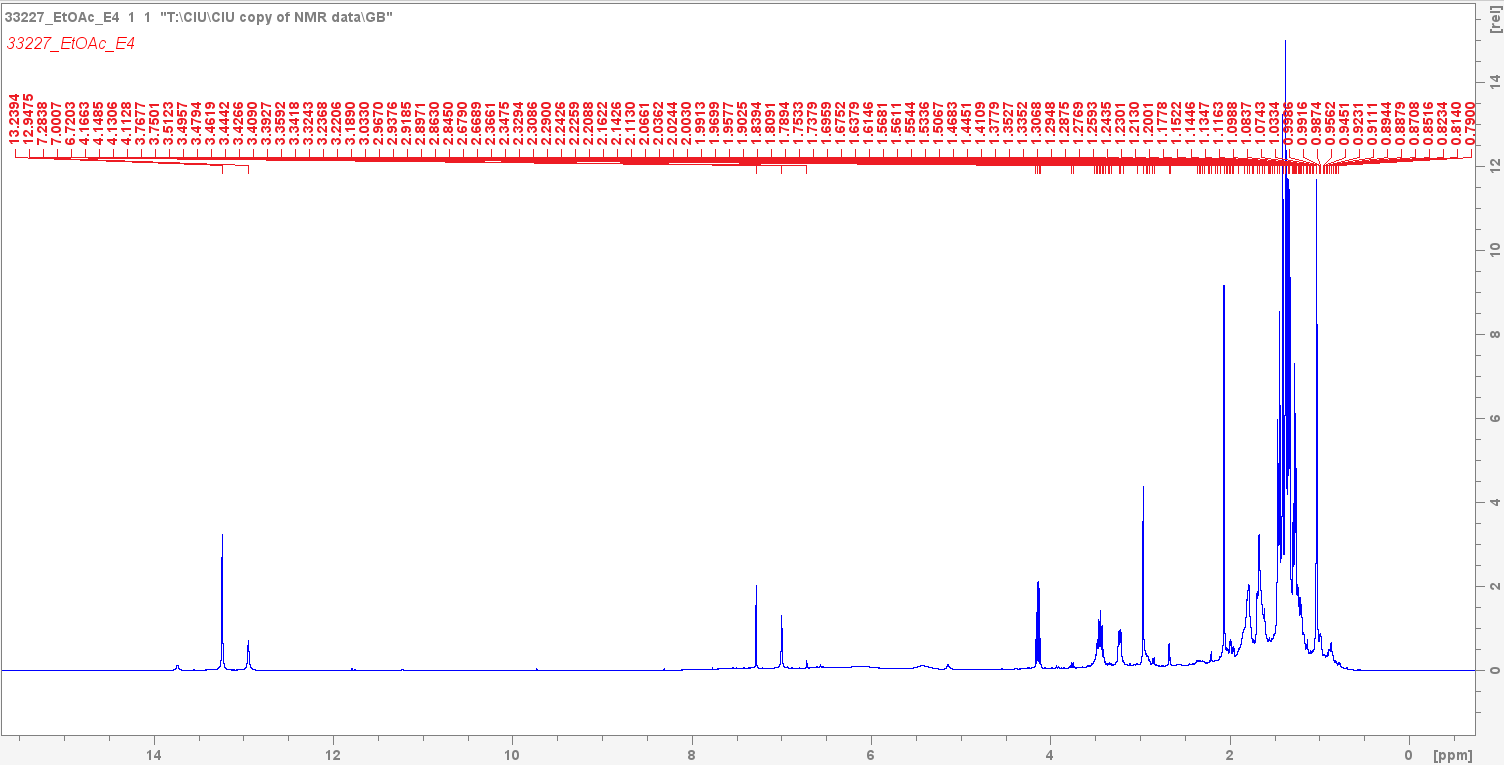


33227_E4


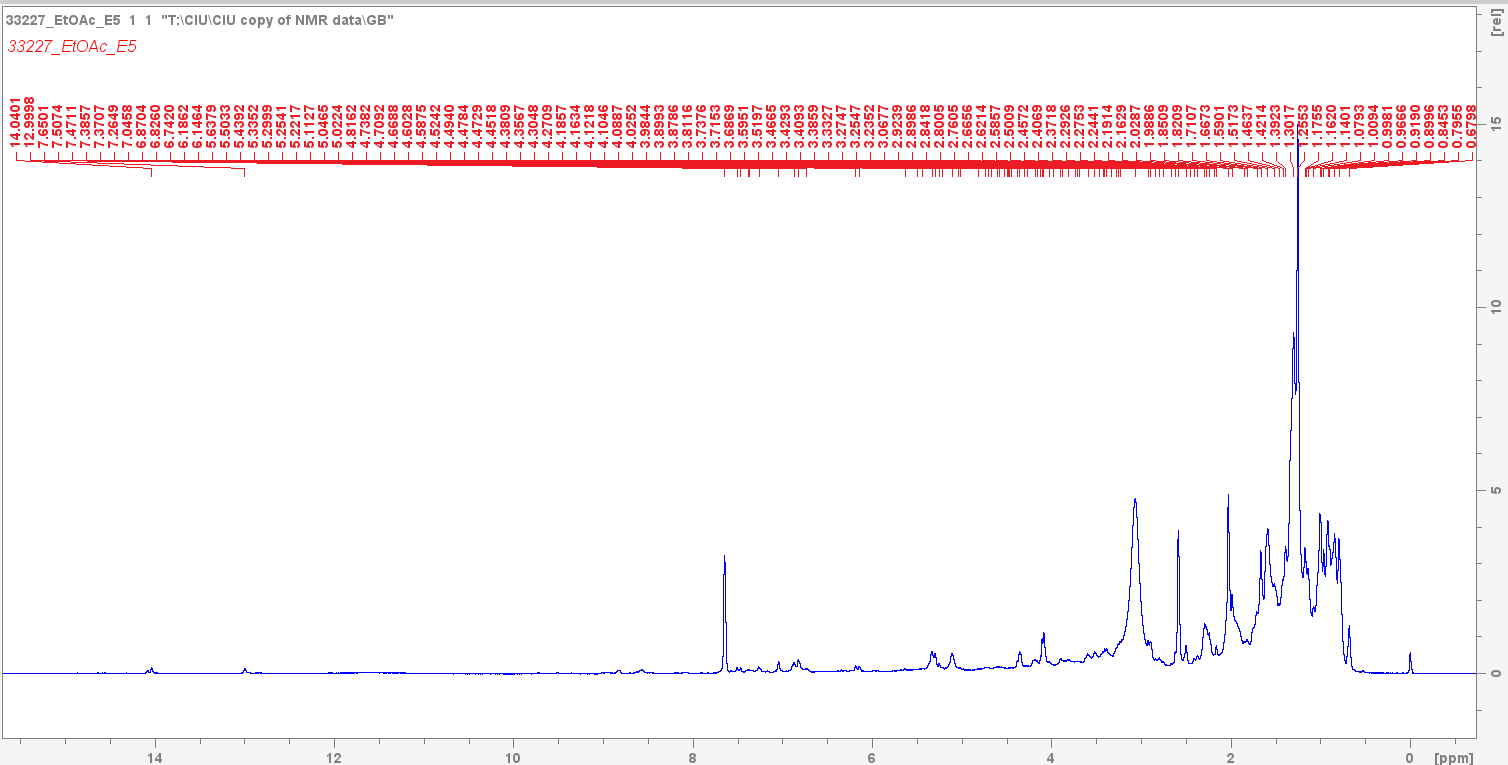


33227_E5


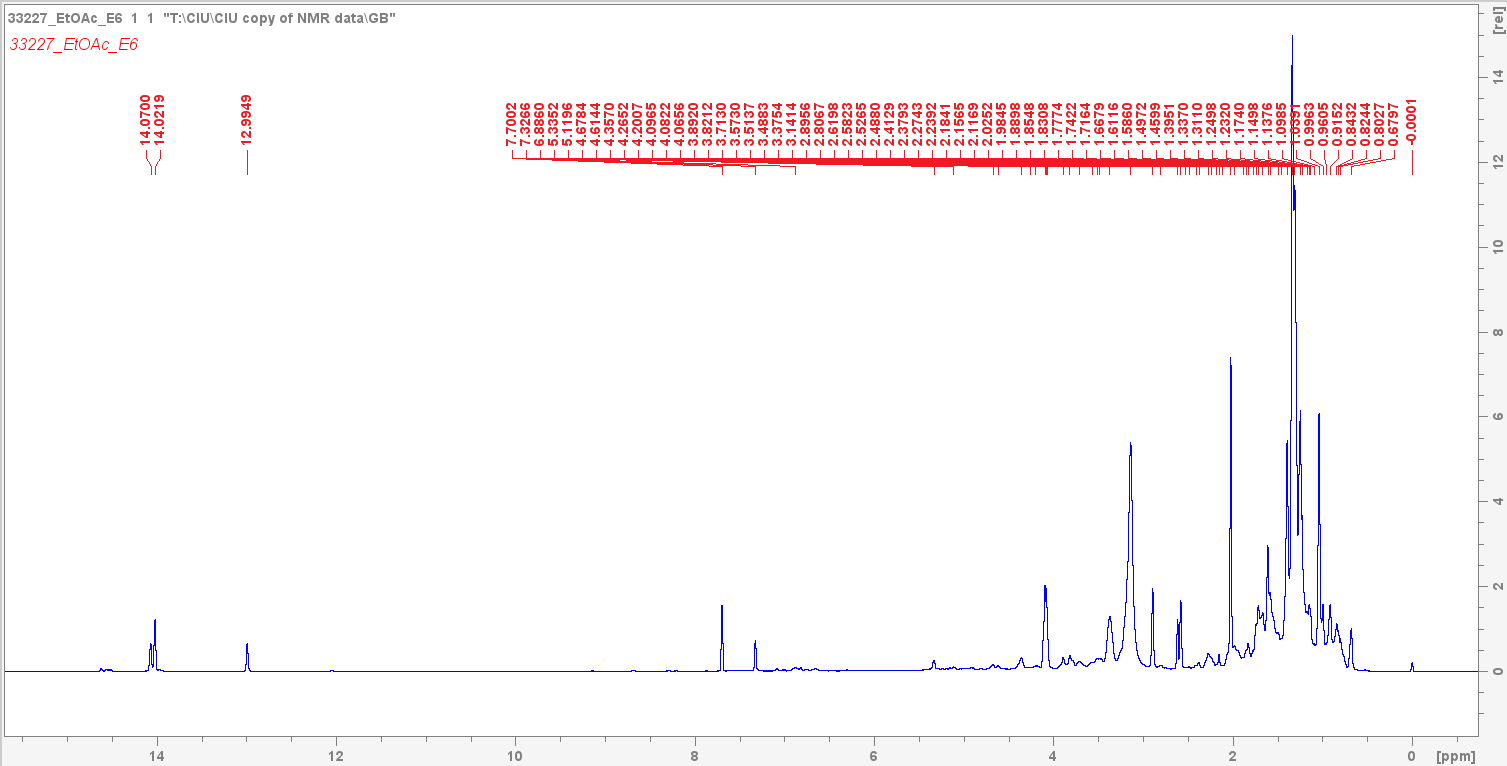


33227_E6


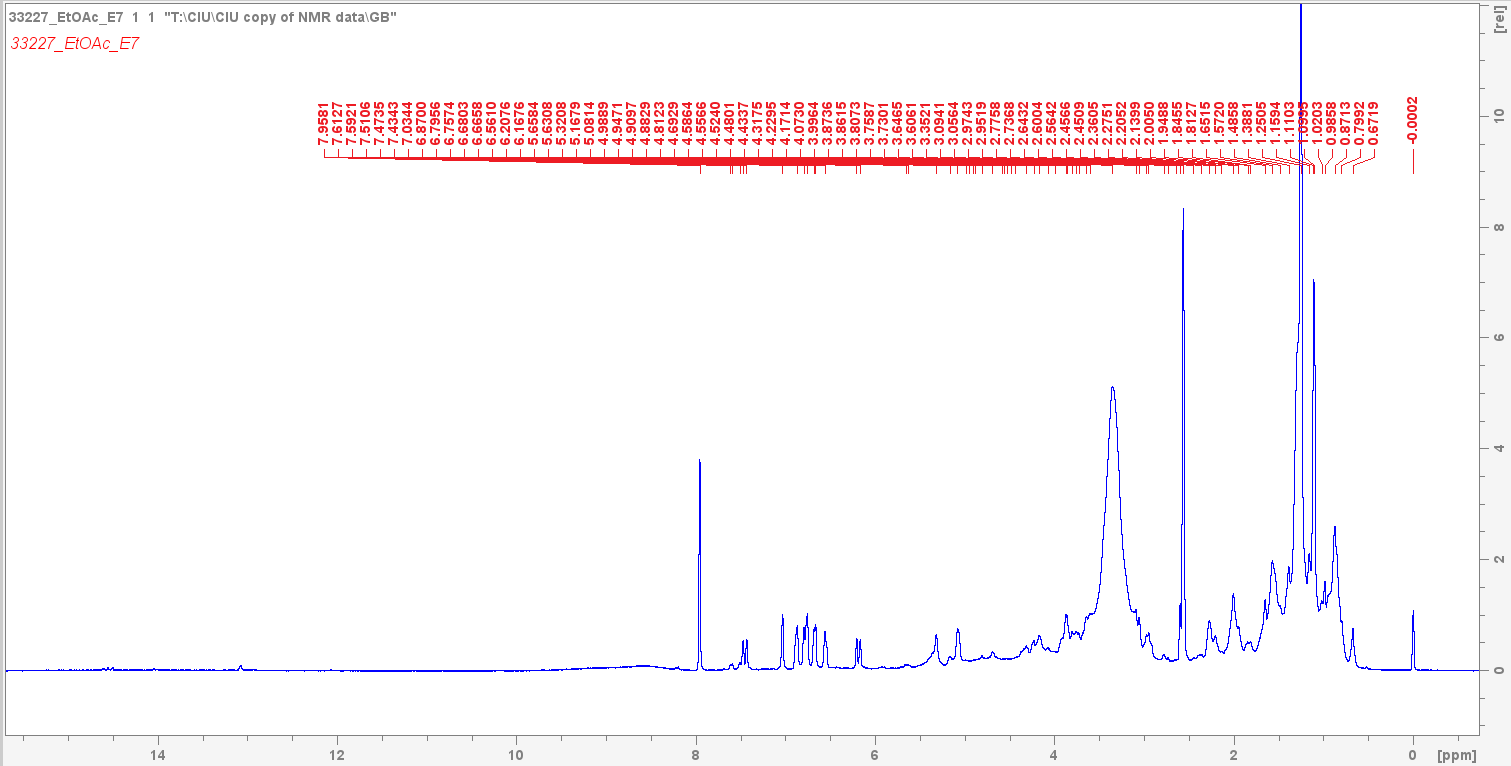


33227_E7


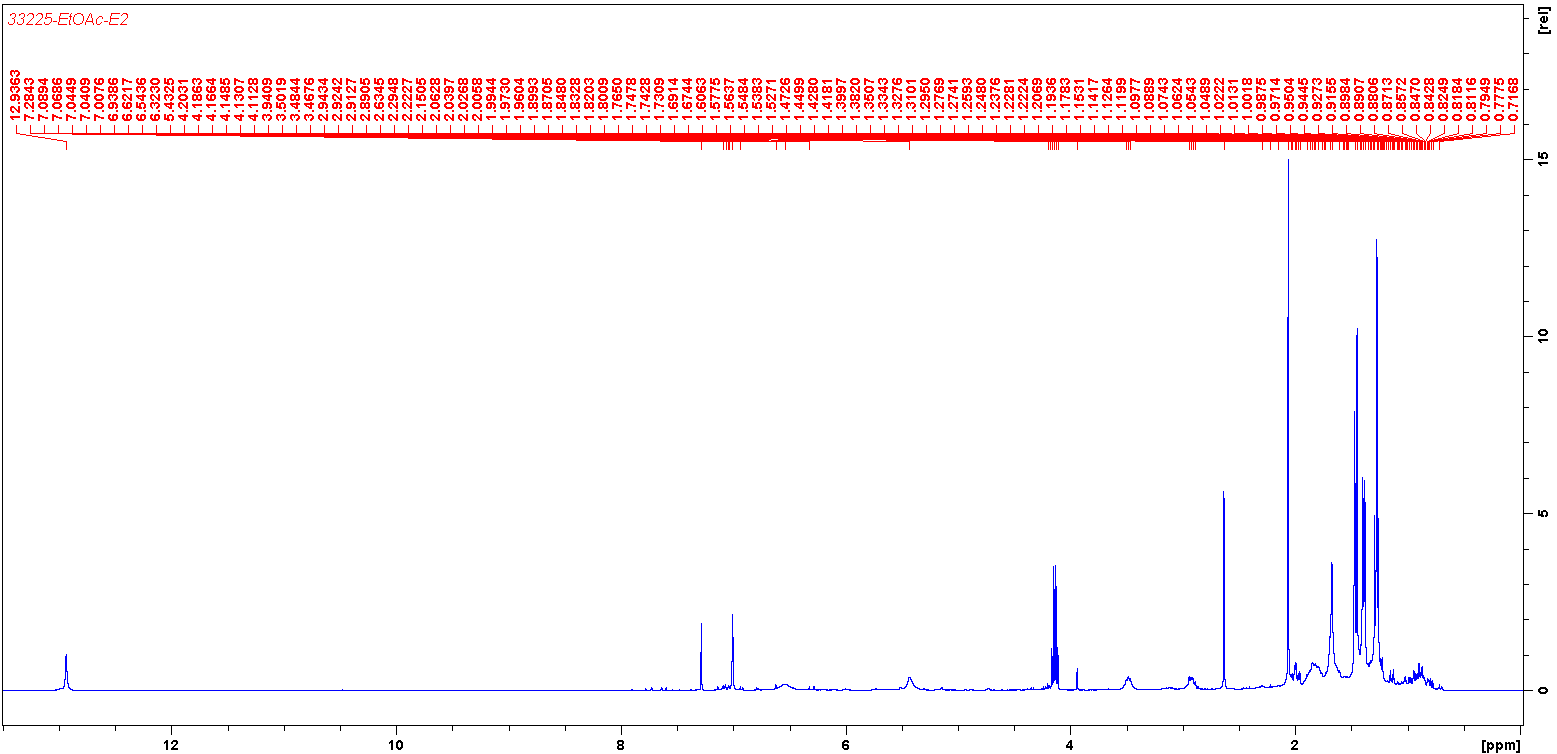


Figure S4. ^1^H NMR spectrum in CDCl_3_ of compound **1**


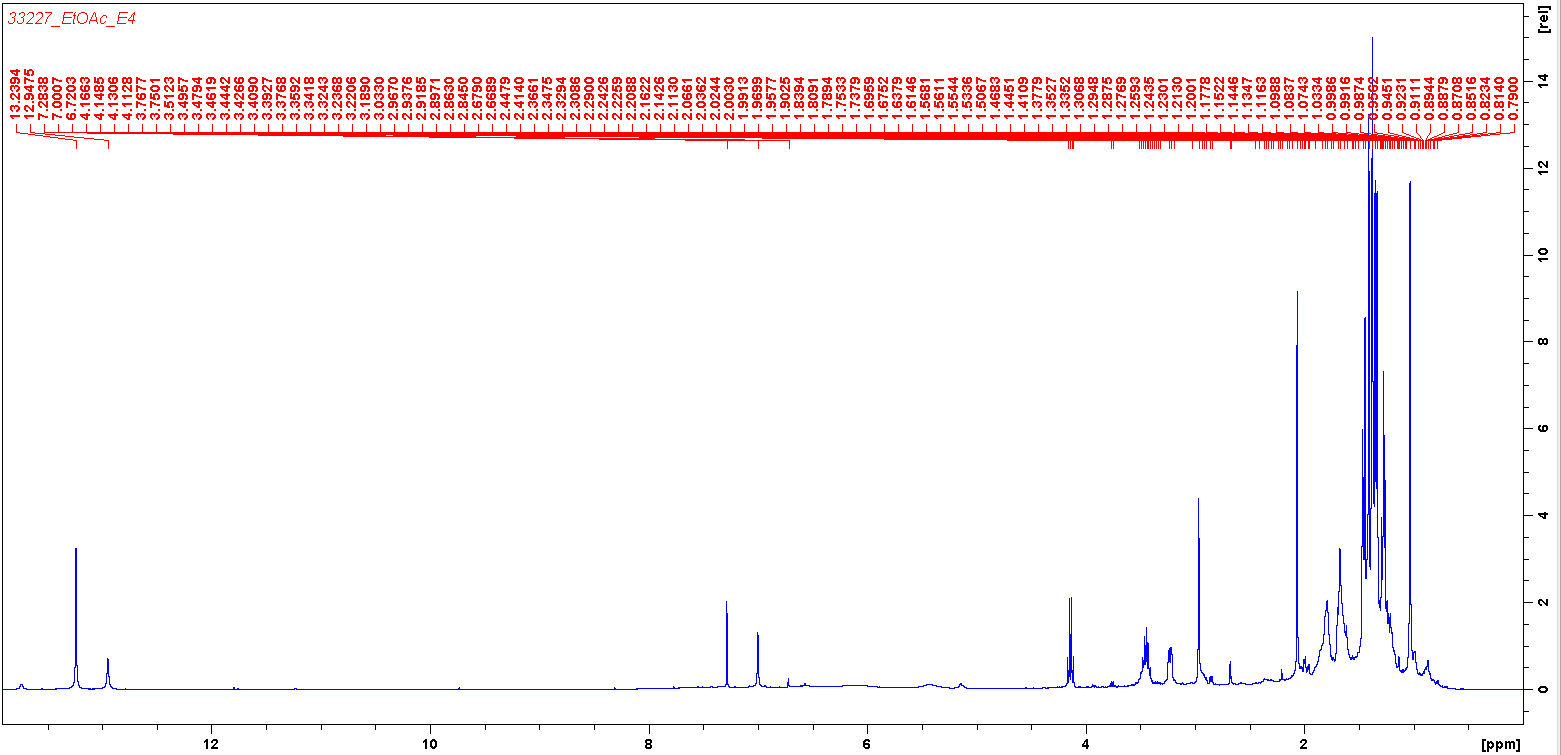


Figure S5. ^1^H NMR spectrum in CDCl_3_ of compound **2**


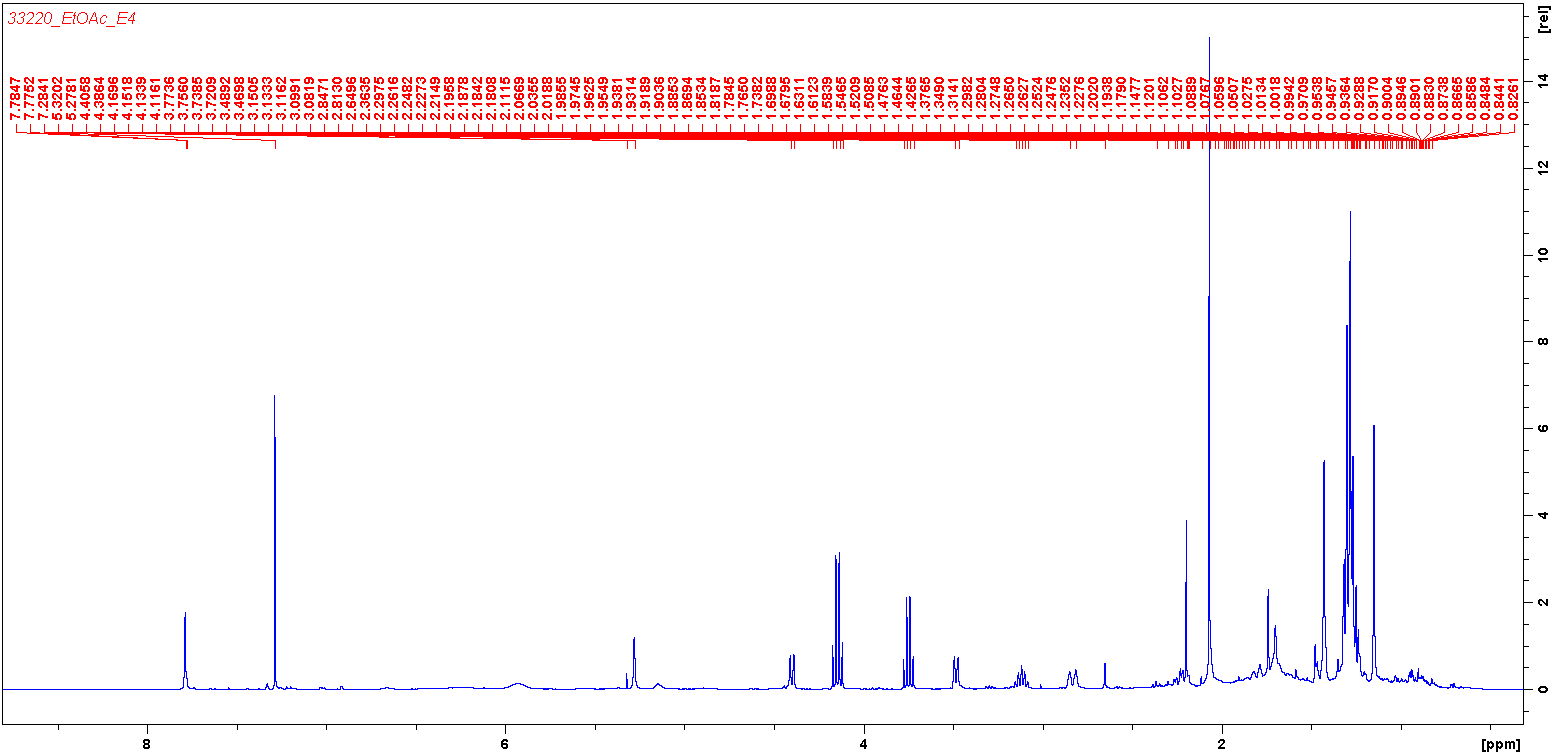


Figure S6. ^1^H NMR spectrum in CDCl_3_ of compound **6**


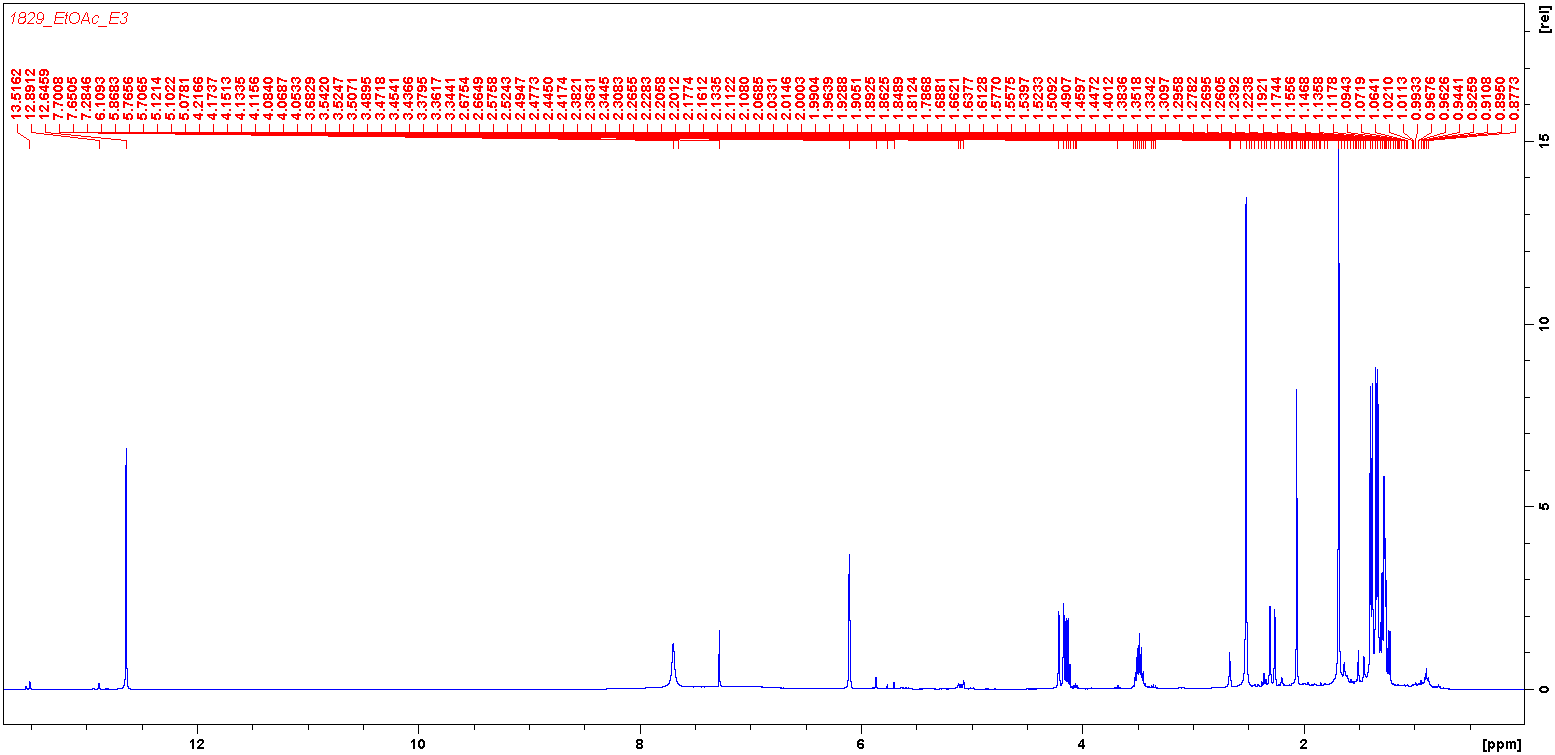


Figure S7. ^1^H NMR spectrum in CDCl_3_ of compound **8**


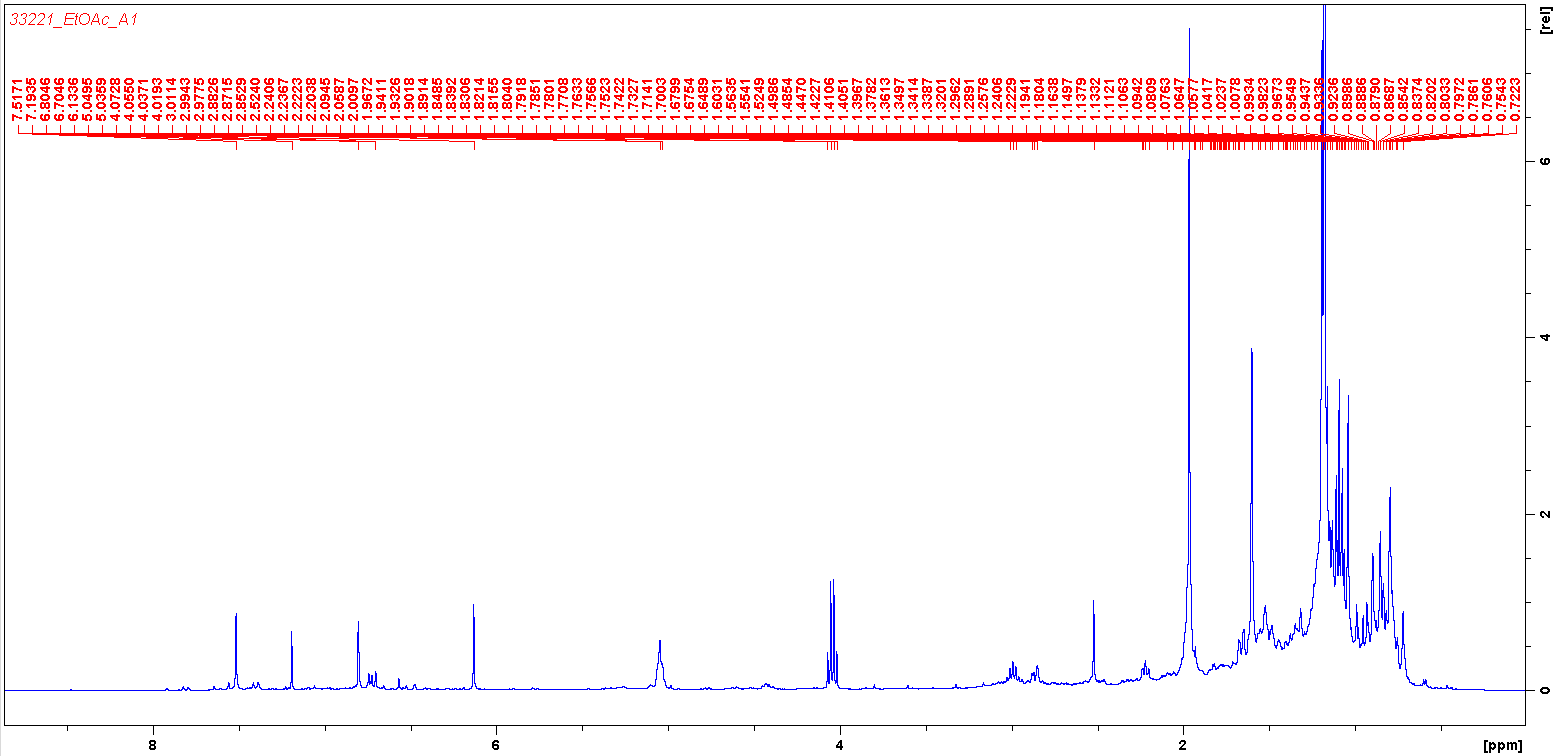


Figure S8. ^1^H NMR spectrum in CDCl_3_ of compound **11**

**
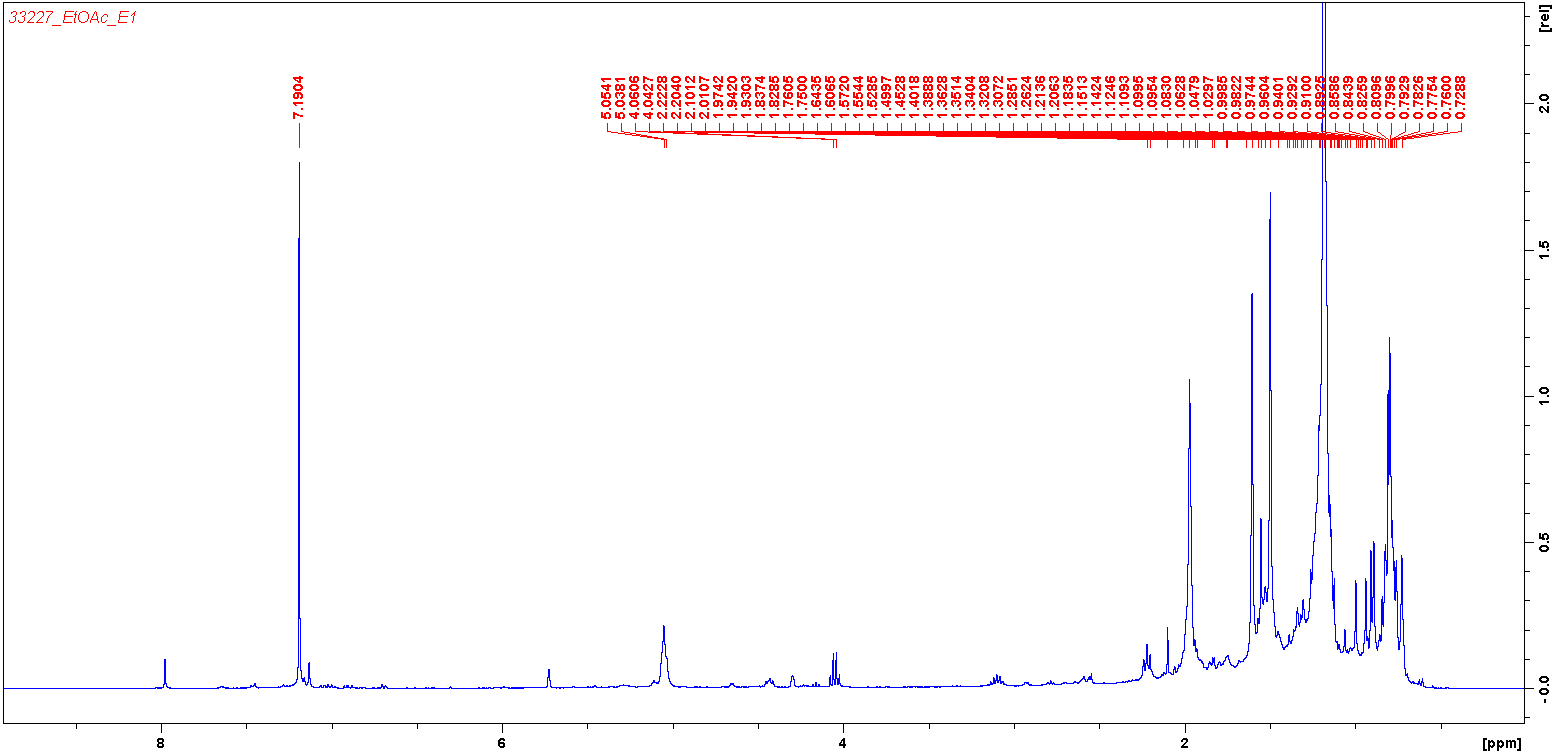
**

Figure S9. ^1^H NMR spectrum in CDCl_3_ of compound **13**


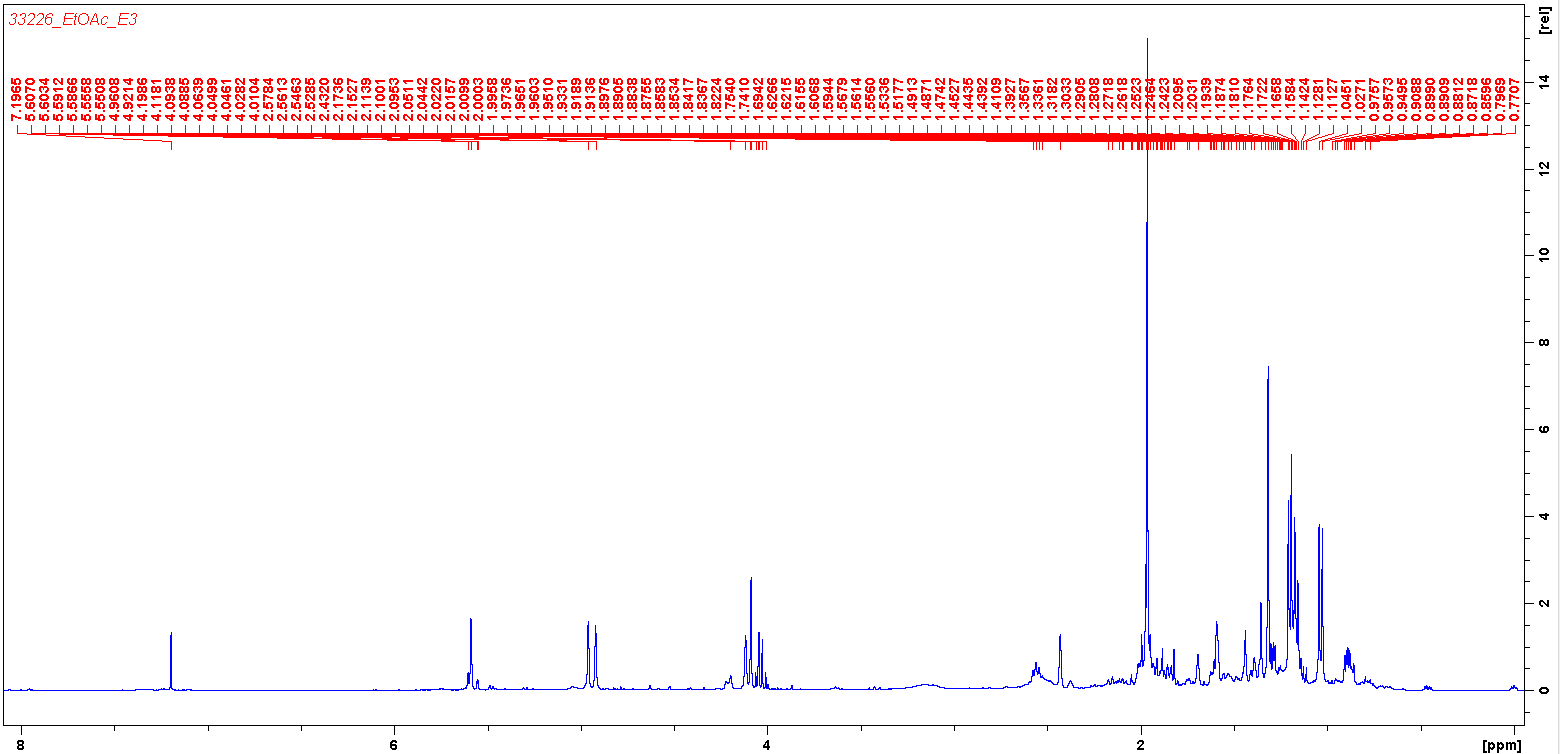


Figure S10. ^1^H NMR spectrum in CDCl_3_ of compound **24**


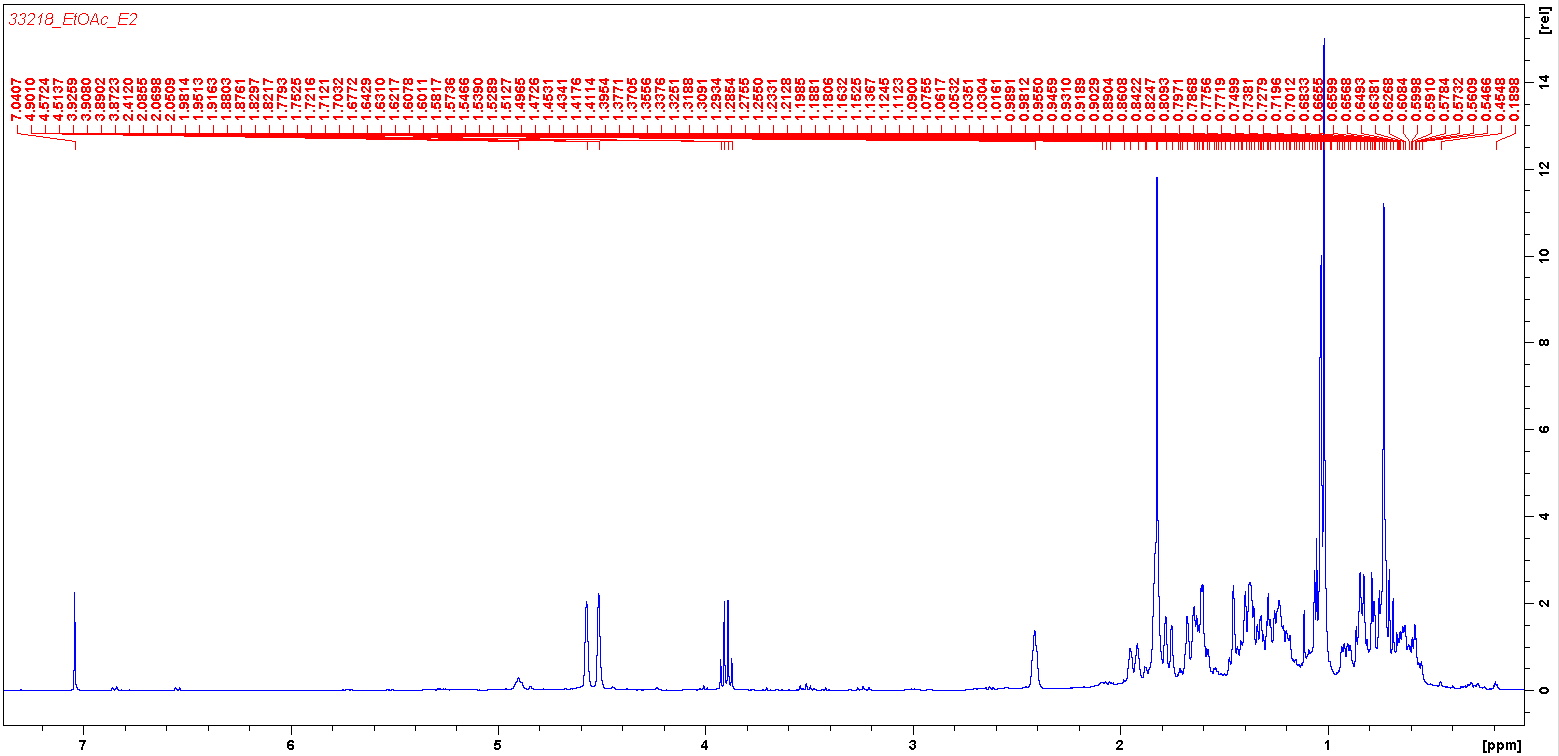


Figure S11. ^1^H NMR spectrum in CDCl_3_ of compound **31**


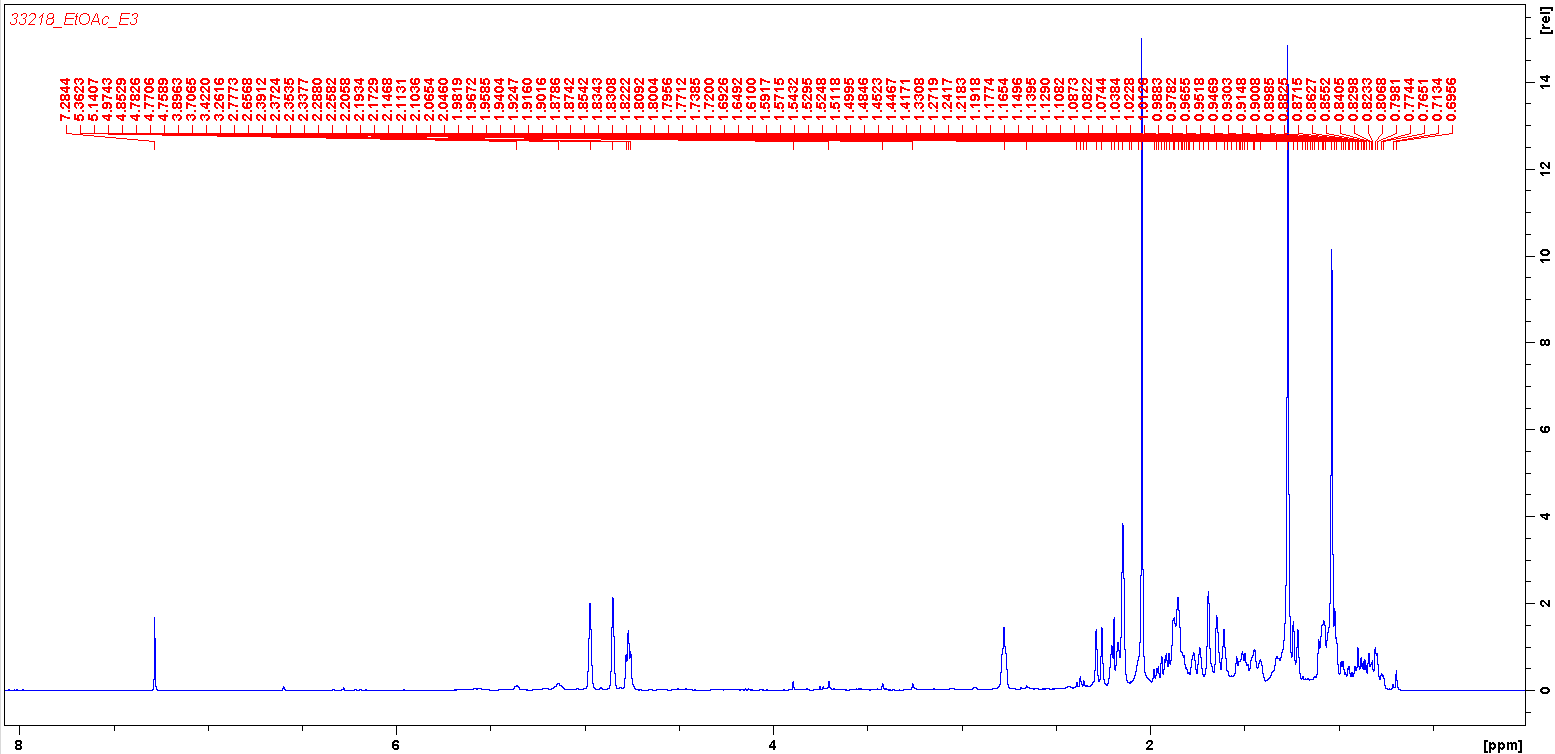


Figure S12. ^1^H NMR spectrum in CDCl_3_ of compound **32**

Figure S13. LCMS traces of the EtOAc of *P. ernstii* (33909)
